# The Molecular Basis of the Intrinsic and Acquired Resistance to Azole Antifungals in *Aspergillus fumigatus*

**DOI:** 10.3390/jof10120820

**Published:** 2024-11-26

**Authors:** Parham Hosseini, Mikhail V. Keniya, Alia A. Sagatova, Stephanie Toepfer, Christoph Müller, Joel D. A. Tyndall, Anette Klinger, Edmond Fleischer, Brian C. Monk

**Affiliations:** 1 Sir John Walsh Research Institute, Faculty of Dentistry, University of Otago, Dunedin 9016, New Zealand; parhamtak@gmail.com (P.H.); mikhail.keniya@hmh-cdi.org (M.V.K.); alia.sagatova@einsteinmed.org (A.A.S.); stephanie.toepfer@i-med.ac.at (S.T.); 2 Department of Pharmacy, Center for Drug Research, Ludwig-Maximilian University Munich, 81377 Munich, Germany; christoph.mueller@cup.uni-muenchen.de; 3 School of Pharmacy, University of Otago, Dunedin 9054, New Zealand; joel.tyndall@otago.ac.nz; 4 MicroCombiChem GmbH, iNovaParc, 56283 Halsenbach, Germany; anette.klinger@microcombichem.com (A.K.); edmond.fleischer@microcombichem.com (E.F.)

**Keywords:** *Aspergillus fumigatus* CYP51, ERG6, CPRA, eburicol, fluconazole, voriconazole, posaconazole, innate and acquired azole resistance, *Saccharomyces cerevisiae*

## Abstract

*Aspergillus fumigatus* is intrinsically resistant to the widely used antifungal fluconazole, and therapeutic failure can result from acquired resistance to voriconazole, the primary treatment for invasive aspergillosis. The molecular basis of substrate specificity and innate and acquired resistance of *A. fumigatus* to azole drugs were addressed using crystal structures, molecular models, and expression in *Saccharomyces cerevisiae* of the sterol 14α-demethylase isoforms AfCYP51A and AfCYP51B targeted by azole drugs, together with their cognate reductase AfCPRA2 and AfERG6 (sterol 24-C-methyltransferase). As predicted by molecular modelling, functional expression of CYP51A and B required eburicol and not lanosterol. A crowded conformationally sensitive region involving the BC-loop, helix I, and the heme makes AfCYP51A T289 primarily responsible for resistance to fluconazole, VT-1161, and the agrochemical difenoconazole. The Y121F T289A combination was required for higher level acquired resistance to fluconazole, VT-1161, difenoconazole, and voriconazole, and confirms posaconazole, isavuconazole and possibly ravuconazole as preferred treatments for target-based azole-resistant aspergillosis due to such a combination of mutations.

## 1. Introduction

**Figure 1:**
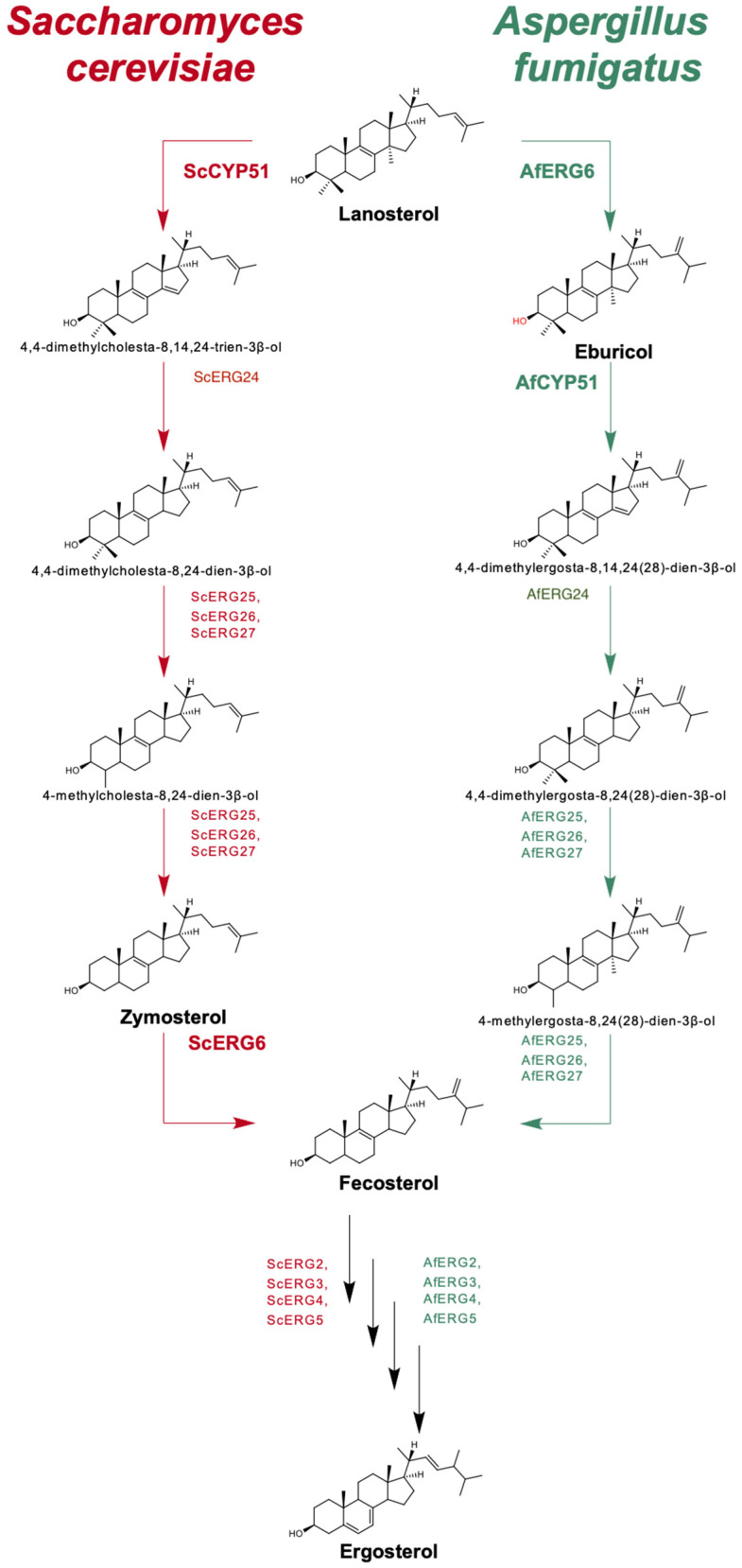
Ergosterol biosynthesis from lanosterol in the yeast *S. cerevisiae* (**left**) and the mould *A. fumigatus* (**right**).

*Aspergillus* infections affect a broad cross-section of the population and have a wide range of clinical manifestations. These include cutaneous aspergillosis of the skin, hypersensitive responses in allergic *Aspergillus* sinusitis, allergic bronchopulmonary aspergillosis, and chronic conditions such as aspergilloma and chronic pulmonary aspergillosis [1,2,3,4]. Invasive aspergillosis (IA) is considered the most important pathology caused by *Aspergillus* species in terms of disease management and mortality [5]. About 15% of allogeneic transplant patients become infected with *Aspergillus fumigatus,* and mortality rates of 40–90% are associated with IA [6]. Treatments for IA rely heavily on triazole drugs, but *A. fumigatus* is intrinsically resistant to the inexpensive, short-tailed, first-generation triazole fluconazole (FLC) [7]. The short-tailed second-generation triazole voriconazole (VCZ) has instead become the preferred antifungal used to treat *Aspergillus* infections. Despite widespread clinical application, VCZ is not completely effective as the primary treatment for IA, and the more expensive long-tailed triazole posaconazole (PCZ) is usually reserved for salvage therapy. Furthermore, exposure to azole drugs in the clinic or azole pesticides in agricultural settings have selected strains of *A. fumigatus* resistant to VCZ [8,9]. The fungal cytochrome P450 enzyme sterol 14α-demethylase (CYP51) is an essential enzyme in the ergosterol biosynthetic pathway and the target for the azole drugs (Figure 1). 

Humans and yeast, such as *Saccharomyces cerevisiae* and pathogenic *Candida* species, have a single CYP51 and can use lanosterol as the principal substrate. However, some moulds and mucoromycetes carry at least two genes that encode CYP51s with differential susceptibilities to azole drugs and may use one or more substrates such as lanosterol, eburicol, or even obtusifoliol [10]. The C-24 methyltransferase enzyme (Erg6) in *S. cerevisiae* converts zymosterol to fecosterol late in the ergosterol biosynthesis pathway and is not essential, but *A. fumigatus* ERG6 converts lanosterol into the CYP51 substrate eburicol and is essential [11]. *A. fumigatus* has two CYP51 paralogs (AfCYP51A and AfCYP51B) with about 60% identity [12]. Gene deletion experiments suggest AfCYP51A confers intrinsic resistance to FLC but not to VCZ or PCZ, while AfCYP51B is susceptible to all three drugs [12,13].

Mutations that modify the primary sequence of drug targets and alter the conformation of their drug binding sites can reduce target affinity for drugs and potentially compromise the treatment of patients. For example, mutations located in the BC-loop within the CYP51 active site, such as the Y132F mutation in *Candida albicans* and *Candida parapsilosis,* and the structurally aligned Y145F mutation in *Cryptococcus neoformans* confer acquired resistance to short-tailed azoles [14,15]. Acquired azole resistance in *A. fumigatus* can be due to single or double mutations in the *A. fumigatus* CYP51A enzyme, together with a tandem repeat in the *CYP51A* promoter region that increases the level of enzyme expression. For example, AfCYP51A TR46/Y121F/T289A and AfCYP51A TR34/L98H mutants appear to result from environmental exposure to azole agrochemicals and confer resistance in azole naive patients to VCZ [16,17,18].

The molecular basis of intrinsic resistance to FLC by AfCYP51A has yet to be elucidated. The AfCYP51A I301 residue was claimed to be responsible for the conferral of intrinsic FLC resistance based on the observation that an AfCYP51A I301T mutation increased susceptibility to FLC to a level comparable to that of a strain deleted of AfCYP51A [19]. This hypothesis requires further testing as structural analysis suggests that the equivalent residue T322 in *S. cerevisiae* CYP51 (ScCYP51) (PDB ID: 4WMZ) is too distant from FLC to directly affect its binding within the lanosterol 14α-demethylase active site and does not modify azole susceptibility (Figure S1, Table S1). By creating and characterizing functional, full-length *A. fumigatus* CYP51A and CYP51B isoforms expressed in an appropriately engineered *S. cerevisiae* host system, we have determined the following: (1) eburicol but not lanosterol is the substrate of both AfCYP51A and AfCYP51B, (2) intrinsic FLC resistance in *A. fumigatus* is conferred by AfCYP51A and not by AfCYP51B, and (3) the AfCYP51A Y121F and AfCYP51A Y121F T289A mutations confer resistance to FLC, VCZ, the agrochemical difenoconazole (DCZ) and the tetrazole VT-1161 (international non-proprietary name oteseconazole) but not the triazoles PCZ, isavuconazole (IVC) and ravuconazole (RVC). Such biochemical and structural understanding of the mechanisms responsible for intrinsic or acquired resistance to the azole drugs aids the identification and design of more effective antifungals that target fungal CYP51s.

## 2. Materials and Methods

### 2.1. Yeast Strains and Culture Conditions

The yeast strains used in this study are described in Table S2 and summarised in Table 1. The *S. cerevisiae* strain ADΔΔ (Y1857) [20] was used as a host to overexpress recombinant enzymes from *A. fumigatus* strain Af293 [21]. Recombinant yeast strains were cultured in yeast extract–peptone–dextrose (YPD) medium containing 1% (wt/vol) Bacto yeast extract (BD Difco Laboratories Inc, Franklin Lakes, NJ, USA), 2% (wt/vol) Bacto peptone (BD Difco), 2% (wt/vol) glucose, or synthetic defined (SD) medium consisting of 0.67% (wt/vol) yeast nitrogen base without amino acids (Formedium, Norfolk, UK), 2% (wt/vol) glucose, and 0.79 g/L complete supplement mixture (CSM, Formedium). The media was solidified by adding 1.8% (wt/vol) agar (BD Difco) as required. Uracil or histidine prototroph transformants were selected on complete supplement mixture without uracil (SD-URA) or without histidine (SD-HIS) agar plates by using 0.77 g/L complete supplement mixture (CSM) drop-out uracil (Formedium) or CSM drop-out histidine (Formedium).

### 2.2. Materials

Codon-optimised *A. fumigatus Cyp51A-6×His*, *AfCyp51B-6×His*, *AfCprA1-6×His*, *AfCprA2-6×His, and AfErg6-FLAG* gene sequences were purchased from ATUM (Newark, CA, USA) in plasmids. Desalted oligonucleotides (Tables S3 and S4) were obtained from Integrated DNA Technologies. PCR amplifications were carried out using Phusion green hot start II high-fidelity PCR master mix (ThermoFisher Scientific, Waltham, MA, USA). DNA transformation of *S. cerevisiae* cells was performed using an Alkali-Cation^TM^ Yeast Transformation kit (MP Biomedicals, LLC, Solon, OH, USA) according to the manufacturer’s instructions. The azole drugs FLC, VCZ, PCZ, and agrochemical DCZ were purchased from Sigma-Aldrich (Auckland, New Zealand). Micafungin (MCF) was provided by Astellas Pharma Inc. (Osaka, Japan). VT-1161 was synthesised by MicroCombiChem (Wiesbaden, Germany).

### 2.3. Construction of Recombinant Yeast Strains

Transformation cassettes were prepared by recombinant PCR. They include the codon-optimised *AfCyp51A* or *AfCyp51B* coding region, together with sequences encoding a C-terminal GRR linker and hexahistidine tag upstream of the PGK1 transcription terminator and *Ura3* selection marker (Figure S2). The transformation cassettes were bordered upstream and downstream by sequences homologous to the *PDR5* promoter and *PDR5* downstream elements. Constructs were inserted by homologous recombination at the *PDR5* locus of the *S. cerevisiae* ADΔΔ host strain. The strains transformed with *AfCyp51A-6×His* or *AfCyp51B-6×His* were named A and B, respectively. Using a similar approach, codon-optimised genes *AfCprA1-6×His* and *AfCprA2-6×His* encoding the NADPH–cytochrome P450 reductase enzymes were transformed separately into the *PDR15* locus of strains A or B (Figure S3), together with replacement of the *PDR15* promoter with the *PDR5* promoter. The constructs included downstream of the ORF the *PGK1* terminator followed by the *HIS1* selective marker flanked with *LoxP* sites. The strains co-expressing AfCPRA2-6×His with AfCYP51A-6×His or AfCYP51B-6×His and retaining the native *ScCyp51* were denoted as AR and BR, respectively. The hexahistidine tag was deleted using a modified cassette created by recombinant PCR, and the *HIS1* selective marker was removed from the *AfCprA1-6*×*His* construct at the *PDR15* locus using the Cre recombinase expressing plasmid pSH69 with galactose activation (GenBank# HQ412578; Euroscarf, Scientific Research and Development GMBH, Oberursel, Germany). The endogenous *ScCYP51* gene of the AR and BR strains was then replaced by homologous recombination with a codon-optimised *AfErg6* construct that included a C-terminal FLAG tag (Figure S4). The *AfErg6-FLAG* construct was prepared by inserting the *PDR5* promoter upstream and the T_synth27_ synthetic terminator [22] plus *HIS1-LoxP* downstream of the recombinant *AfErg6-FLAG* gene. On replacement of the native *ScCyp51*(*ScERG11*) gene, subcultured transformants were recovered on SD selective medium after 3–4 days. These strains were named ARE and BRE based on their putative expression of *AfCyp51A* or *AfCyp51B*, respectively (Figure S5).

Mutant constructs designed to express AfCYP51A-6×His Y121F, T289A, I301T, and Y121F T289A were prepared by PCR using genomic DNA from strain ARE as a template and incorporated at the *PDR5* locus in *S. cerevisiae* strain ARE (Figure S6). Transformation cassettes encoding desired single amino acid mutations were used separately to transform *S. cerevisiae* strain ARE-h (a derivative of strain ARE with the *His1* gene downstream of the *AfERG6-6×His* deleted) at the *PDR5* locus using selection on SD-HIS drop-out medium. The only differences between the mutated fragments and the extant *AfCyp51A*-*6×His* cassette in the *PDR5* locus were the nucleotide substitution(s) and the selective marker gene at the 3′-end of the cassette. Because of the similarity of the fragments and the targeted region, the majority of the transformants were expected to incorporate only the selective marker and not the mutated region of the AfCYP51 ORF. Therefore, for each transformation, numerous colonies were tested by DNA sequence analysis to identify suitable mutants.

Transformation of the double mutation CYP51A Y121F T289A was performed in two steps. In the first step, a cassette containing the T289A mutation was transformed into the ARE-h strain. In the second step, a short fragment of a PCR product encoding the CYP51A Y121F was amplified from the Y121F single mutant. This fragment was transformed into the strain carrying the T289A mutation. Solidified YPD medium containing 0.7 μM VCZ was used to select transformants (Figure S6).

To simplify their description, recombinant CYP51A mutant strains have been annotated according to the mutation carried (Table 1). The ADΔΔ host strain and strains ADLS or Y2300 that overexpress ScCYP51 with a C-terminal hexahistidine tag and with the native *ScCyp51* gene deleted were used as negative and positive controls, respectively, to estimate the relative expression of His-tagged proteins in genetically modified strains. Strain Y2411, with the *URA3* marker from plasmid pABC [23] inserted at the *PDR5* locus of strain ADΔΔ, was used as a negative control in some experiments.

### 2.4. Phenotypic Analysis of Yeast Strains

Agarose diffusion tests in SD medium containing 10 mM MES and 20 mM HEPES adjusted with Tris to pH 6.8 (SD pH 6.8) were used to visually assess the susceptibility of recombinant yeast strains to different antifungal drugs [24]. Twenty mL of SD pH 6.8 containing 0.6% agarose was poured into a rectangular OmniTray (Thermo Scientific, Waltham, MA, USA) and allowed to set. A portion (100 μL) of a 2 mL SD pH 6.8 overnight culture of the test strain was used to inoculate 3 mL of fresh SD pH 6.8. This culture was incubated for 4 to 6 h at 30 °C to generate logarithmic-phase cells. The yeast culture was added to 20 mL 0.6% agarose SD pH 6.8 at 50 °C to give OD_600nm_ = 0.0078 (~10^5^ CFU/mL) and solidified at room temperature over the solidified bottom layer. A total of 5 μL of drug solutions were added to sterile 6 mm paper discs (Becton, Dickinson, and Company (BD), Franklin Lakes, NJ, USA) that were then placed on the top agar. The plate was incubated at 30 °C for 48 h.

The susceptibilities of *S. cerevisiae* strains to antifungal drugs were determined as minimum growth inhibitory concentration (MIC) values in 200 μL SD pH 6.8 [24]. Serial 2-fold dilutions of 100 μL of azole or control drugs in SD pH 6.8 were prepared in a 96-well microtiter plate. A portion of a 2 mL SD pH 6.8 overnight culture of the test strain was inoculated into 3 mL fresh SD pH 6.8 and incubated for 4 to 6 h at 30 °C to generate logarithmic-phase cells. These cells were then diluted with fresh SD pH 6.8 to OD_600nm_ = 0.01 (3 × 10^4^ CFU), and 100 μL of this cell suspension was added to microtiter plate wells. Cell growth after 48 h incubation at 30 °C with shaking at 200 rpm was determined at 600 nm using a BioTek™ Synergy™ 2 multi-Mode microplate reader controlled using Gen5™ Data Analysis Software version 3.16.10 (BioTek Instruments, Winooski, VT, USA). MIC_80_ values (80% growth inhibition in the presence of the test drug compared with the untreated control) for each yeast construct were determined as technical triplicates repeated three times for three separate clones.

### 2.5. Confirmation of DNA Sequences in Recombinant loci in S. cerevisiae

Extraction of genomic DNA was achieved using a Yeast DNA Extraction Kit (Thermo Fisher Scientific, Waltham, MA, USA). DNA sequence analysis was carried out at the Genetic Analysis Services Facility (University of Otago, Dunedin, New Zealand). Figure S7A shows the confirmatory sequences obtained.

### 2.6. Confirmation of Recombinant Protein Expression

Crude membranes were obtained from 5 mL YPD overnight cultures of recombinant yeast cells according to a miniaturised method [23]. Larger scale crude membrane preparations were obtained from 6 L overnight YPD cultures of recombinant yeast cells using the method described for *S. cerevisiae* CYP51-6×His (ScCYP51-6×His) [25]. The protein concentration of crude membrane fractions was estimated using the Lowry method [26] according to the Bio-Rad DC Protein Assay kit protocol for microtiter plates with bovine serum albumin (BSA, Sigma Aldrich) as the standard protein. The proteins in the crude membrane were separated by SDS–polyacrylamide gel–electrophoresis (SDS-PAGE) in 8% acrylamide gels using the method of Laemmli [27]. Protein bands were visualised by staining with Coomassie blue R250. Proteins separated by SDS-PAGE were transferred onto a polyvinylidene fluoride (PVDF) membrane (Amersham Hybond ECL, GE Healthcare, Auckland, New Zealand) using the Bio-Rad Mini Trans-Blot^®^ Cell. Blots were blocked with milk and immunodecorated with a peroxidase-conjugated mouse monoclonal antibody: Anti-6×His tag antibody (Sigma Aldrich SAB2702219) or mouse Anti-FLAG M2-peroxidase antibody (Sigma Aldrich A8592). Decorated and washed PVDF membranes were incubated in freshly prepared Clarity Max Western ECL Substrate (Bio-Rad 1705062). Enhanced chemiluminescence (ECL) was detected using the ChemiDoc MP Imaging System (Bio-Rad, Hercules, CA, USA).

Mass spectrometry of tryptic digests of protein bands excised from SDS-PAGE separated crude membrane preparations was carried out using an LTQ-Orbitrap hybrid mass spectrometer at the University of Otago Centre for Protein Research. The fragments obtained were searched against the Swiss-Prot database, the sequence of interest, and *S. cerevisiae* to exclude possible background contamination (Figure S7B).

### 2.7. Purification of Recombinant Proteins

AfCYP51-6×His isoforms were purified according to the method described for ScCYP51-6×His [28]. Crude membranes were solubilised with 18 mM [10× critical micelle concentration (CMC)] of the detergent n-decyl-β-D-maltoside (DM) (Anatrace, Maumee, OH, USA) in 20 mM Tris pH 7.5, 250 mM NaCl, 10% (wt/vol) glycerol, 0.5 mM PMSF and 1 EDTA-free protease inhibitor pill (Roche, Basel, Switzerland) per 200 mL of solution. A 1 mL HisTrap^TM^ HP protein purification column (GE Healthcare Bio-Sciences AB, Uppsala, Sweden) was used to selectively bind the detergent solubilised recombinant AfCYP51-6×His proteins. Affinity column-bound His-tagged proteins were usually washed with 20 mM imidazole and eluted using 200 mM imidazole. When AfCYP51A or AfCYP51B were purified for spectrophotometric analysis of type I binding of substrates or type II binding of azole drugs, imidazole was replaced by 10 mM and 100 mM L-Histidine in the washing and elution buffers, respectively. Eluted fractions were washed and concentrated to 1 mL using Amicon^®^ Ultracel^®^-50K Centrifugal Filters (Merck Millipore Ltd., Cork, Ireland) in the presence of 7.2 mM (4×CMC) of DM in 20 mM Tris pH 7.5, 250 mM NaCl, 10% (wt/vol) glycerol, 0.5 mM PMSF and 1 EDTA-free protease inhibitor pill (Roche, Basel, Switzerland) per 200 mL of solution.

### 2.8. Biochemical Analysis of Recombinant CYP51s

#### 2.8.1. Type I and Type II Binding

The concentration of AfCYP51A and AfCYP51B, purified using Ni-NTA affinity chromatography and used for the spectrophotometric type I and type II binding assays, was calculated using an extinction co-efficient of λ_417nm_ = 117 mM^−1^ cm^−1^ applied to the absolute absorption spectrum of the preparations [10]. In addition, the amount of “active” cytochrome P450 enzyme was determined using the carbon monoxide (CO) binding spectra of these preparations [29].

Type I binding of substrates lanosterol and eburicol in the active site of the Ni-NTA affinity-purified AfCYP51A and AfCYP51B enzymes was assessed using the method of Warrilow et al. [30]. Titrations of 5 μM AfCYP51-6×His protein with lanosterol or eburicol (1 to 250 μM) were measured using a Cary 300 UV–Visible Spectrophotometer (Agilent Technologies, Santa Clara, CA, USA). Substrate saturation curves were constructed by plotting the maximal $∆A_{peak-trough}$ obtained between 350 and 500 nm, allowing for a change in the reaction volume during the titration. The substrate binding constant (*K_S_*) was calculated by nonlinear regression (Levenberg–Marquardt algorithm) applied to the Michaelis–Menten equation or the Hill equation. GraphPad Prism 9.2.0 software was used to identify the best fit for the data.

Type II binding difference spectra were obtained using 1 μM of affinity-purified CYP51 enzymes. VCZ, PCZ, VT-1161, DCZ, and MCF working solutions were prepared in DMSO, while FLC was prepared in Milli-Q water. Azole drugs were added to the sample cuvette, and corresponding amounts of DMSO or Milli-Q water were added to the reference cuvette. Drug binding curves were plotted using the difference spectrum peak and trough detected in the range of 410–425 nm. Dissociation constants (*K_d_*) were determined by nonlinear regression (Levenberg–Marquardt algorithm) of the difference in absorbance between the peak and trough against the azole concentration using a rearrangement of the Morrison equation or the Hill equation [7]. GraphPad Prism 9.2.0 software was used to identify the best fit for the data.

#### 2.8.2. Sterol Analysis of Recombinant Yeast Strains

Yeast strains grown overnight in SD medium pH 6.8 were inoculated in 20 mL fresh medium and grown to OD_600nm_ = 0.5. The cultures were then treated with VCZ at a concentration that reduced the rate of cell growth by ~50%. Untreated control cultures were harvested by centrifugation after 8 h at a cell density of ~2.5, and VCZ-treated cultures were harvested after ~16 h at a similar cell density. The harvested cells were prepared for sterol extraction and analysis using the method described by Müller et al. [31].

### 2.9. Construction and Application of the AfCYP51A Homology Model

Homology model structures of AfCYP51A were generated using either the AfCYP51B (PDB ID: 4UYM) or *Homo sapiens* CYP51 (HsCYP51, PDB ID: 6UEZ) crystal structures using Modeller 9.22 or 10.4, respectively [32]. The AfCYP51A homology models, crystal structures of AfCYP51B, and ScCYP51 in complex with azole antifungals (PDB IDs: 4WMZ, 5HS1) were superimposed using the PyMOL Molecular Graphics System, Version 2.0 (Schrödinger, LLC, New York, NY, USA). Structural alignment of the crystal structure of the human CYP51 D231A/H314A mutant (PDB ID: 6UEZ) with the AfCYP51A homology model was used to capture the comparable conformation of lanosterol in the AfCYP51A binding site. The docking of eburicol and lanosterol in AfCYP51A models was obtained using GOLD flexible ligand docking software, version 2022.2.0.

### 2.10. Synthesis and Quality Control of Compounds Prepared by MicroCombiChem

Information on the route of synthesis and compound quality control (NMR analysis and GC-MS) is provided in Figures S8 and S9.

## 3. Results

### 3.1. Bioinformatic and Structural Comparison of CYP51s

**Figure 2:**
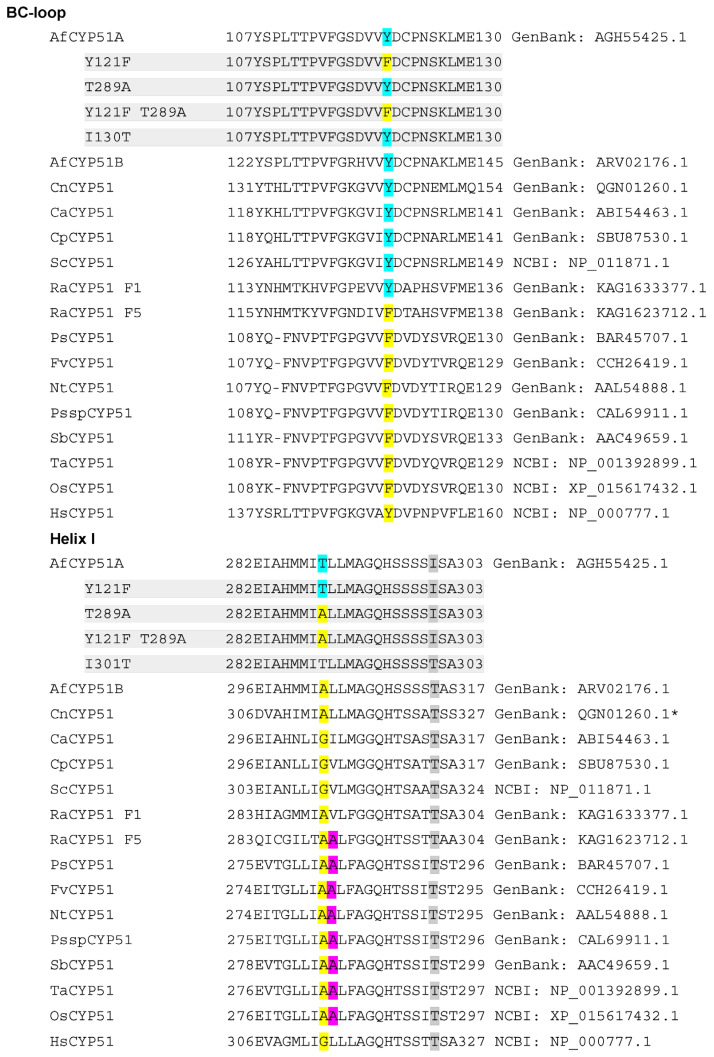
Alignment of BC-loop and helix I regions of CYP51 proteins. AfCYP51A and AfCYP51B were aligned with the AfCYP51 mutants (on grey background) and with CYP51s from *Cryptococcus neoformans* (CnCYP51), *Candida albicans* (CaCYP51), *Candida parapsilosis* (CpCYP51)*, Saccharomyces cerevisiae,* (ScCYP51), *Rhizopus arrhizus* (RaCYP51 F1 and F5 isoforms), *Pisum sativum* (garden pea, PsCYP51), *Fragaria vesca* (wild strawberry FvCYP51), *Nicotiana tabacum* (tobacco NtCYP51), Petunia hybrid ssp (PsspCYP51), *Sorghum bicolor* (Sorghum SbCYP51), *Triticum aestivum* (wheat TvCYP51), *Oryza sativa* (rice OsCYP51), and *Homo sapiens* (HsCYP51) using CLC Sequence Viewer 8.0 software (Aarhus, Denmark). The residues equivalent to Y121 and T289 (highlighted in turquoise) in AfCYP51A are intrinsically substituted with phenylalanine and alanine in plant Obtusifoliol 14α-demethylases, and the *R. arrhizus* CYP51 F5 isoforms (highlighted in yellow), respectively. RaCYP51 F5 and the plant CYP51s also substitute the residue immediately downstream of the alanine with another alanine (highlighted in magenta). The residues equivalent to AfCYP51A I303 (highlighted in dark grey) in helix I are substituted with T in CYP51 from plants, fungi, and humans.

**Figure 3:**
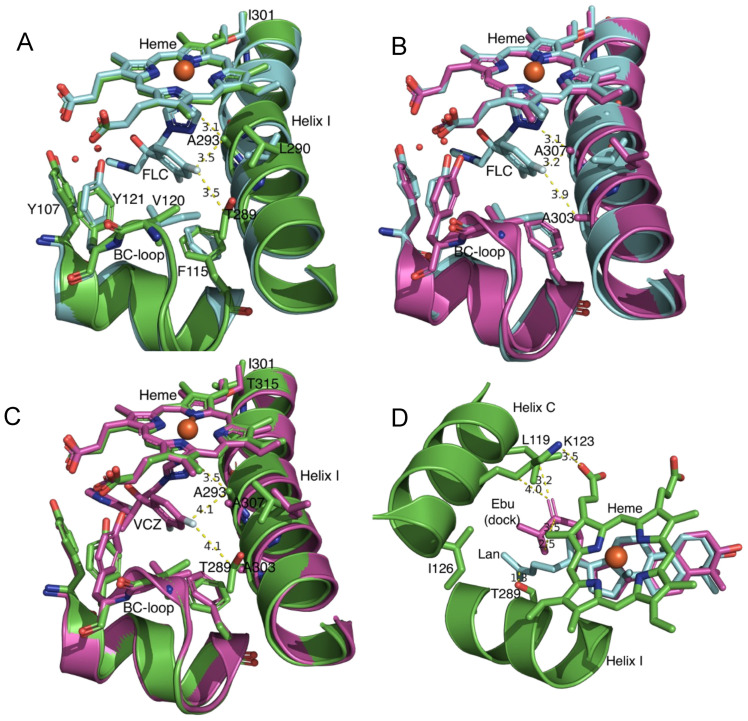
Superimposition and docking studies with the AfCYP51A (green) homology model. The AfCYP51A homology model (**A**,**C**,**D**) was obtained using the AfCYP51B crystal structure (**B**) as a template and superimposed with lanosterol in the HsCYP51 crystal structure (**D**). The heme, helix I, and BC-loop from crystal structures for ScCYP51 (turquoise) in complex with FLC (PDB ID: 4WMZ) and AfCYP51B (purple) in complex with VCZ (PDB: 4UYM) are also shown. (**A**) AfCYP51A model (green) superimposed on ScCYP51 in complex with FLC (turquoise). The 4-fluorine of the FLC difluorophenyl group is surrounded by V120/I139, F115/F134, T289/G310, and L290/V311 in AfCYP51/ScCYP51. ScCYP51 G310 aligns with the larger polar T289 in AfCYP51A. At the closest approach, FLC is 8.8 Å from L301 in the AfCYP51A helix I. AfCYP51A Y107 and Y121 align with ScCYP51 Y126 and Y140, respectively, which contribute to water-mediated hydrogen bond interactions with FLC in ScCYP51. (**B**) AfCYP51B crystal structure (purple) superimposed on the crystal structure of ScCYP51 in complex with FLC (turquoise). (**C**) AfCYP51A model (green) superimposed on the crystal structure of AfCYP51B in complex with VCZ (purple). (**D**) AfCYP51A homology model with heme, helix I, and helix C in green. Lanosterol from the HsCYP51 crystal structure (PDB ID: 6UEZ) is overlaid, and the top-scoring docked eburicol (Ebu (dock)) conformation is shown. Distances in the figures are provided in Å.

Cross-species primary sequence alignment (Figure 2), together with analyses of crystal structures and structural models (Figure 3), indicated that the CYP51A Y121F and T289A mutations and the T289A substitution within the CYP51A active site are likely to selectively affect FLC binding.

The crystal structure of ScCYP51 in complex with FLC (PDB ID: 4WMZ) identified key interactions that included its binding to the heme iron via a triazole group, a hydrogen bond network involving its tertiary alcohol, a water molecule, Y140 and heme propionates, plus a hydrogen bond network involving the second triazole, a water molecule, Y126 and the main chain carbonyl of S382 [28]. Alignment of ScCYP51, AfCYP51A, and AfCYP51B showed that the ScCYP51 aromatic residues Y126, F134, Y140, and F236 plus G315 are conserved in all three enzymes (Figure 2). ScCYP51 M509 aligns with AfCYP51A L494 and AfCYP51B L503, and these residues are therefore unlikely to confer isoform-specific FLC resistance. The residues aligned with ScCYP51 I139, V311, G314, T318, and L380 show conservation of their hydrophobicity or polarity. Of these, G314 at the centre of helix I aligns with A293 in AfCYP51A and A307 in AfCYP51B. Alignment of the ScCYP51 crystal structure with the AfCYP51A homology model based on the AfCYP51B crystal structure in complex with VCZ (PDB ID: 4UYM) places the hydrophobic AfCYP51A A293 side chain methyl carbon within 3.1 Å of the heme-bound FLC triazole and 3.5 Å from C3 of the phenyl ring of the FLC difluorophenyl group (Figure 3A). Other residues close to (<4 Å) in the same difluorophenyl group include the conserved aromatic residues F214 (3.7 Å from the FLC 2-fluorine) and F115 (3.8 Å from the phenyl C3) and the hydrophobic residue V120 (3.9 Å from the FLC 4-fluorine). Similarly, the superposition of FLC on the AfCyp51B crystal structure with VCZ placed the A307 methyl group 3.1 Å from the heme-bound FLC triazole group, and 3.2 Å from C3 of the FLC phenyl ring that parallels helix I (Figure 3B). The superposition of the FLC from 4WMZ on 4UYM shows the distance between the FLC 4-fluorine and AfCYP51B A303 β-carbon is 3.9 Å (Figure 3B). In helix I, the CYP51A T289 residue (Figure 2) is larger and more polar than the aligned ScCYP51 G310 and AfCYP51B A303 residues. Alignment of the AfCYP51A model with the crystal structure of ScCYP51 in complex with FLC (PDB ID: 4WMZ) shows the close proximity (3.5 Å) of the AfCYP51A T289 β-carbon to the FLC 4-fluorine group bordering helix I (Figure 3A). This distance is significantly greater for the 4-fluorine of the VCZ (4.1 Å) when the AfCYP51A model and the AfCYP51B structure are aligned, as illustrated in Figure 3C. The distance is slightly less (3.8 Å) to T389 when VCZ from the crystal structure with ScCYP51 (PDB: 5HS1) is superimposed instead.

While these data suggest T289 differentially affects FLC versus VCZ binding by the AfCYP51A model, different impacts (such as the alternative orientation of the triazole) due to induced fit by AfCyp51B with VCZ and by the slightly different ScCYP51 with FLC cannot be excluded (Figure 3A–C). Four turns along helix I from AfCYP51A T289 and >8.8 Å from FLC, the previously studied AfCYP51A I301 residue [19] aligns with threonine in both ScCYP51 and AfCYP51B (Figure 2). Furthermore, expression of the ScCYP51 T322I mutant in yeast did not change the susceptibility to FLC, VCZ or ITC (Table S1), alter the conformation of the enzyme, or the ligands FLC and ITC, despite the loss of hydrogen bonding capacity between the T322 hydroxyl and the S318 main chain carbonyl group (see Figure S1, and crystal structures PDB IDs: 4WMZ, 5ESM, 5EQB, 5ESL). Collectively, these data suggested that T289 in AfCYP51A is more likely than I301 to confer innate resistance to FLC but not VCZ. In contrast, the Y140F mutation in the *S. cerevisiae* BC-loop (Figure 2) and structurally aligned mutations in other fungal pathogens equivalent to AfCYP51A Y121F are known to confer acquired resistance to both FLC and VCZ [20]. While VCZ is bound tightly (1-1 with fungal CYP51 enzymes), FLC is bound with a more modest affinity, possibly indicative of competition with the enzyme’s sterol substrate.

We explored the possibility that the tight space between the helix I, BC-loop, and C-helix might differentially affect the binding of substrates such as lanosterol and eburicol and their competition with FLC. For this purpose, we used the AfCYP51A model based on the AfCYP51B crystal structure and an AfCYP51A model based on the crystal structure of HsCYP51 in complex with lanosterol (PDB ID: 6UEZ). The HsCYP51 structure includes D231A H314A mutations that enable high occupancy by lanosterol in the substrate binding site and provide insight into conformational features required for capture of electrons via interaction of the C-helix with its cognate NADPH–cytochrome P450 reductase [33]. Structural alignment of the HsCYP51 crystal structure with the AfCYP51A homology model based on AfCYP51B crystal structure (PDB ID: 4UYM) indicates that the multiring scaffolds of eburicol and lanosterol have comparable conformations in the CYP51 active sites, but the hydrophobic tail of lanosterol is likely to clash with AfCYP51A T289 and AfCYP51B A303 in helix I (Figure 3D). The top-ranked eburicol conformer docked with the AfCYP51A homology model showed the CH_2_ of the 24,25-dihydro group, found in the hydrophobic tail of eburicol but not lanosterol, was in close contact with the γ carbon of K123 and the γ carbon of L119, while a terminal methyl group was 2.5 Å from the adjacent heme methyl. Theoretical support for the view that eburicol is more compatible than lanosterol as a substrate of AfCYP51A and AfCYP51B was obtained by docking lanosterol and eburicol into a homology model of AfCYP51A obtained using HsCYP51 (6UEZ) as template (Figure S10).

To determine the isoform responsible for innate resistance to FLC, recombinant versions of isoforms AfCYP51A and AfCYP51B were separately functionally expressed in a strain of *S. cerevisiae* hypersusceptible to azole drugs, and phenotypic responses to a range of azole drugs measured. This approach using heterologous expression overcomes the background of drug efflux pumps found in *A. fumigatus*. Recombinant *S. cerevisiae* strains with the AfCYP51A engineered with single or double mutations were then used to confirm the role of AfCYP51A in innate azole resistance and to assess their effects on acquired resistance to VCZ and FLC. In particular, the mutant enzymes AfCYP51A-6×His T289A (strain Y2751), I301T (Y2752), Y121F (Y2750) and Y121F T289A (Y2753) were expressed in *S. cerevisiae* and used to test whether the I301 substitution and the Y121F and T289A mutations in AfCYP51A determine susceptibility to FLC and other antifungals used in medicine and agriculture.

It is notable that the single AfCYP51A T289A mutation has not been detected in clinical isolates or environmental strains, even though the structurally aligned residue in AfCYP51B is A303. In addition, the single Y121F mutation has been reported only once in a clinical isolate [34]. The AfCYP51A T289A mutation has exclusively been found together with Y121F in strains that overexpress CYP51A due to the TR46 promoter repeat. Although both AfCYP51A Y121F [34] and TR46/Y121F/T289A mutations [35] confer resistance to VCZ, the functional relationship between Y121F and T289A mutations has yet to be elucidated.

### 3.2. Characterisation of Recombinant Strains and Mutations

**Figure 4:**
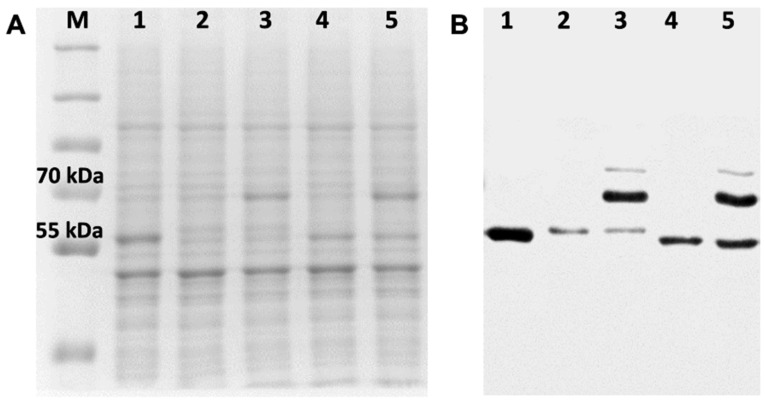
AfCPRA2-6×His but not AfCPRA1-6×His is detected in *S. cerevisiae* strains expressing AfCYP51s. (**A**) Coomassie blue-stained SDS-PAGE gel (8% acrylamide) of 15 μg crude membrane protein samples, and (**B**) Western blot for the same amount of crude membrane protein samples. Proteins tagged with 6×His were detected by ECL using a peroxidase-conjugated mouse monoclonal Anti-6×His tag antibody. Samples: (M) PageRuler™ Plus prestained protein standards (Bio-Rad Laboratories, Auckland, New Zealand), (1) ADLS, (2) AfCPRA1-6×His not detected in strain A expressing AfCYP51A, (3) AfCPRA2-6×His detected in the strain AR expressing AfCYP51A, (4) AfCPRA1-6×His not detected in the strain B expressing AfCYP51B, (5) AfCPRA2-6×His detected in the strain BR expressing AfCYP51B. The AfCPRA2-6×His protein was detected as two bands (~70 kDa and a minor band at ~80 kDa) in Western blot lanes 3 and 5.

**Figure 5:**
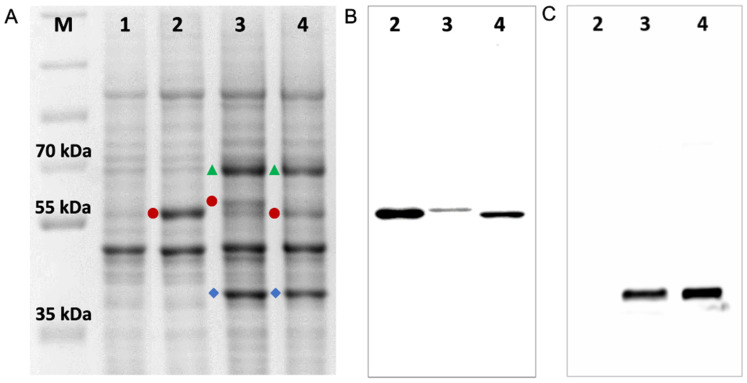
AfCYP51 isoforms are expressed in *S. cerevisiae* with their cognate NADPH–cytochrome P450 reductase (AfCPRA2) and sterol 24-C-methyltransferase (AfERG6) enzymes. (**A**) Coomassie blue-stained SDS-PAGE gel (8% acrylamide) of 30 μg crude membrane protein samples, (**B**) Western blot ECL detection of CYP51-6×His-tagged proteins by a peroxidase-conjugated mouse anti-6×His monoclonal antibody or (**C**) ECL detection of AfERG6-FLAG-tagged proteins by a peroxidase-conjugated mouse Anti-FLAG M2-peroxidase antibody. Samples: (M) PageRuler™ Plus prestained protein standards, crude membrane protein samples from strains (1) ADΔΔ, (2) ADLS, (3) ARE, and (4) BRE. (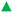) Coomassie-stained AfCPRA2 without 6×His tag expressed from *PDR15* locus of ARE and BRE samples, (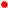) CYP51-6×His, (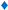) AfERG6-FLAG.

The insertion and location of recombinant genes in strains prepared for this study (Table 1) were checked by PCR of genomic DNA, and the sequences of the genes inserted in the *S. cerevisiae* genome were confirmed by DNA sequence analysis (Figure S7A). Expression of the AfCYP51-6×His isoforms, AfCPRA2-6×His and AfERG6-FLAG was detected using SDS-PAGE and Western blots (Figure 4 and Figure 5). The presence of the AfCYP51-6×His enzymes in crude membranes was confirmed using mass spectrometry of the SDS-PAGE gel bands digested with trypsin (Figure S7B). The expected sizes for AfCYP51A-6×His (with or without the mutations) and AfCYP51B-6×His are 59 kDa and 60 kDa, respectively. Despite this, the His-tagged band of AfCYP51A revealed by Coomassie blue staining of the SDS-PAGE gel and Western blot analysis migrated slightly slower than the bands for AfCYP51B or ScCYP51 (Figure 4 and Figure 5). Western blots showed that the expression of AfCYP51B-6×His was 5-fold greater than AfCYP51A-6×His (Figure 4B). Two His-tagged bands (~70 and ~80 kDa) were detected on the blots for strains expected to express the AfCPRA2 protein (Figure 4, lanes 3 and 5). A 78 kDa band was expected for AfCPRA2-6×His, and the ~70 kDa band is consistent with the N-terminal proteolytic processing of the reductase. In contrast to the expression of AfCPRA2-6×His, strains transformed with a recombinant version of the alternate isoform *AfCprA1-6×His* gave neither an overexpressed Coomassie-stained band nor a detectable His-tagged AfCprA1 band in crude membrane preparations (Figure 4, lanes 2 and 4).

### 3.3. AfErg6 Supports Growth in Recombinant Strains Deleted of Native ScCYP51

In 5 separate experiments, deletion of the host *ScCYP51* gene with the *His1* selective marker using SD-HIS drop-out medium was unsuccessful for the AR strain, while strain BR gave small colonies that were not viable on subculture. As *ScCYP51* is required for ergosterol biosynthesis in *S. cerevisiae*, this observation indicated that AfCYP51A in strain AR and AfCYP51B in strain BR failed to support ergosterol biosynthesis. Based on our modelling studies (Figure 3D and Figure S10), which supported the hypothesis that eburicol is the primary substrate for the AfCYP51s, we set out to enable eburicol synthesis from lanosterol in the recombinant yeast strains expressing AfCYP51A or AfCYP51B. To obtain synthesis of eburicol and delete *ScCYP51*, strains AR and BR were first deleted of the *HIS* marker downstream of *AfCprA2* at the *PDR15* locus. Confirmed His progeny strains were then transformed at the *ScCYP51* locus with *AfERG6*, giving rise to strains ARE and BRE. The recombinant constructs at the *ScCYP51*, *PDR5,* and *PDR15* loci were confirmed by DNA sequence analysis (Figure S7A). SDS-PAGE and Western blot analysis of crude membrane preparations from strains ARE and BRE showed, as expected, an ~70 kDa CPR protein band (Figure 5A, lanes 3 and 4 green triangles) detected using Coomassie blue stain and not with the anti-His tag antibody (Figure 5B lanes 3 and 4), while bands of co-expressed ~60 kDa AfCYP51A-6×His or AfCYP51B-6×His were detected in the same lanes using Coomassie blue stain (red circles) and the anti-His tag antibody. In addition, the expected ~40 kDa AfERG6-FLAG protein was detected as strain-specific Coomassie blue-stained protein bands (Figure 5A, blue diamonds) and with an anti-FLAG antibody (Figure 5C, compare lanes 3 and 4) in the samples from strains ARE and BRE.

**Figure 6:**
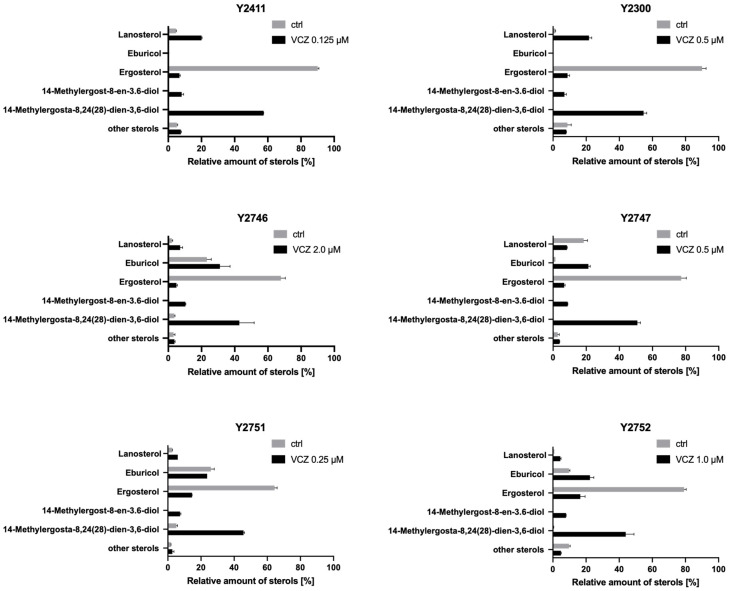
Sterol compositions of recombinant *S. cerevisiae* strains in the absence (ctrl) or presence of VCZ at a concentration that reduced growth rate by ~50%. Y2411 = ADΔΔ; Y2300 = ADLS); Y2746 = ARE, Y2747 = BRE; Y2751 = ARE T289A; Y2753 = ARE I301T.

The production of eburicol in *S. cerevisiae* due to the heterologous expression of the sterol 24-C methyl transferase AfERG6 was tested with strains Y2411 (genotype equivalent of ADΔΔ with *URA3* marker expressed from the *PDR5* locus), Y2300 (genotype equivalent of ADLS), Y2746 (ARE) and Y2747 (BRE) (Figure 6). Cells were grown to OD_600nm_ = 0.5 and then incubated ± VCZ until OD_600nm_ = 2.5. The concentrations of VCZ used were sufficient to slow the growth rate of the strains by ~50%. The sterol content of cell extracts was quantitated using suitable standards by GC-MS. All strains produced ergosterol in quantities that were reduced in the presence of VCZ due to inhibition of CYP51. As expected, each strain also produced large quantities of methyl sterols from lanosterol in the presence of VCZ. The yeast host strain and the yeast strain overexpressing ScCYP51 did not produce eburicol. In contrast, ARE and BRE strains produced eburicol, which was increased in the presence of VCZ. This confirms the introduction of a different pathway from *A. fumigatus* involving AfERG6. While the BRE strain produced small amounts of eburicol (~1% of total sterol) in the absence of VCZ, this increased several-fold (to ~20% of total sterol) in response to VCZ treatment. In contrast, the ARE strain produced substantial quantities of eburicol (20% of total sterol) in the absence of VCZ, and this increased slightly to 25% in the presence of VCZ. This result is consistent with lower (rate limiting) levels of functional CYP51 in ARE compared to BRE, perhaps reflecting the ~5-fold higher levels of recombinant AfCYP51B than AfCYP51A detected in Figure 5.

### 3.4. Drug Susceptibilities of Strains Expressing AfCYP51 Isoforms Indicate AfCYP51A Is Responsible for Innate Resistance to FLC

Drug susceptibilities were quantitatively assessed using MIC_80_ determinations for the recombinant strains overexpressing AfCYP51 isoforms, with or without its cognate reductase AfCPRA2, and AfERG6, plus for the mutant strains Y121F, T289A, Y121F T289A, and I301T (Table 2). As all recombinant strains were obtained using the host strain ADΔΔ, it was used as the reference strain to compare drug susceptibilities. Strain ADLS, which has the native *ScCyp51* deleted and overexpresses ScCYP51-6×His from the *PDR5* locus, was used as a positive control that shows an overexpression-dependent reduction in susceptibility to azole drugs but not to the CYP51-independent control echinocandin antifungal drug MCF. Susceptibilities to the short-tailed triazoles FLC and VCZ, the long-tailed triazole PCZ, the medium-tailed tetrazole VT-1161, and the triazole agrochemical DCZ shown in Table 2 were supported by agarose diffusion assays (Figure S11).

The recombinant strains A, B, AR, and BR showed comparable susceptibilities for each of the azole drugs FLC, VCZ, VT-1161, PCZ, and the agrochemical DCZ as the ADΔΔ strain, which retains ScCYP51. None of these strains showed azole resistance greater than the ScCYP51 overexpressing ADLS strain, consistent with the innate ScCYP51 dominating azole susceptibility. The BRE strain showed susceptibilities to these drugs comparable to those of the ADLS strain, apart from PCZ and DCZ, which gave susceptibilities comparable to ADΔΔ. In contrast, except for PCZ and the echinocandin MCF, strain ARE was significantly more resistant to the azoles tested than the reference ADΔΔ and ADLS strains. The MIC_80_ value of the ARE strain for FLC (227 ± 19 μM) shows that the collective expression of AfCYP51A, AfCPRA2, and AfERG6 conferred high-level resistance to FLC (Table 2). In contrast, the BRE strain, which functionally expresses AfCYP51B, AfCPRA2, and AfERG6, had an MIC_80_ for FLC of 10.0 ± 1.4 μM comparable to the ADLS strain (8.0 ± 0.2 μM). The latter value, due to ScCYP51 overexpression, was 3-fold higher than the MIC_80_ of the ADΔΔ strain [28]. Strain ARE but not BRE also conferred strong resistance to both VT-1161 (MIC_80_ = 0.43 ± 0.05 μM) and DCZ (MIC_80_ = 0.64 ± 0.04 μM), with MIC values several-fold higher than that conferred by the ADLS strain (MIC_80_ = 0.06 ± 0.00 μM and 0.04 ± 0.02 μM, respectively). In contrast, the MIC_80_ values of the ARE and BRE strains for VCZ were 0.27 ± 0.03 μM and 0.21 ± 0.02 μM, respectively. These values were 4- to 5-fold higher than those obtained with ADΔΔ (MIC_80_ = 0.06 ± 0.00 μM) and comparable to that obtained with strain ADLS (MIC_80_ = 0.17 ± 0.03 μM). This more modest increase in VCZ resistance can be attributed to functional overexpression of the recombinant AfCYP51s. Strain ARE (MIC_80_ = 0.10 ± 0.01 μM) was equally susceptible to the long-tailed azole PCZ as ADΔΔ and BRE and slightly more susceptible than strains A (MIC_80_ = 0.21 ± 0.01 μM) or AR (MIC_80_ = 0.19 ± 0.01 μM). The MIC_80_ values and agarose diffusion assays (Figure S11A) indicate that BRE was slightly more susceptible to MCF than the other strains tested, but this difference was not statistically significant (Table 2).

### 3.5. Phenotypes of Strain ARE CYP51A Mutants

**Figure 7:**
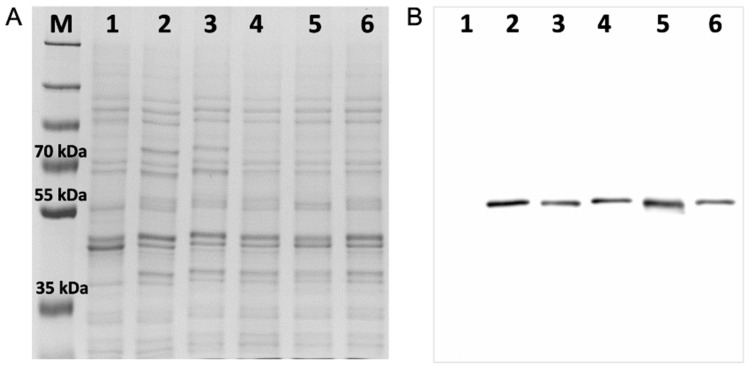
Expression of AfCYP51A-6×His mutant constructs in *S. cerevisiae*. (**A**) Coomassie blue-stained SDS-PAGE gel (8% acrylamide) of 15 μg crude membrane protein samples, and (**B**) Western blot ECL detection of 6×His-tagged proteins for equivalent loadings of each crude membrane protein sample. Proteins tagged with 6×His were detected by ECL using a peroxidase-conjugated mouse monoclonal Anti-6×His tag antibody. Samples: (M) PageRuler™ Plus prestained protein standards; crude membrane protein samples from strains (1) ADΔΔ, (2) ARE, (3) Y121F, (4) T289A, (5) Y121F T289A, and (6) I301T.

Coomassie blue-stained SDS-PAGE gels and Western blot analysis of crude membrane fractions from strains ADΔΔ, ARE, Y121F, T289A, Y121F T289A, and I301T (Figure 7) showed that the strains with single mutations produced slightly less AfCYP51A-6×His than strain ARE, while the strain with the double mutation produced slightly more. Sterol analysis confirmed the functionality of the ARE derivative strains T289A and I301T, with VCZ inhibition of ergosterol biosynthesis and their production of eburicol demonstrated (Figure 6). The relative amounts of eburicol and ergosterol detected in the untreated strains suggest that strains ARE and T289A both metabolize eburicol slowly, I301T has an intermediate level of activity, and BRE has the highest turnover rate for this substrate. VCZ inhibited ergosterol biosynthesis in both mutant strains and increased the production of toxic 14-methyl sterols.

Compared to parental strain ARE, the AfCYP51A-6×His mutants created in this study showed different susceptibilities to the azole drugs but comparable susceptibilities to the control drug MCF (Table 2). The MIC_80_ values of the mutant strains T289A (MIC_80_ = 21.3 ± 2.7 μM) and I301T (MIC_80_ = 77.2 ± 5.9 μM) for FLC showed, respectively, 11-fold and 3-fold greater susceptibility than strain ARE (MIC_80_ = 227 ± 18 μM). The MIC_80_ value of strain T289A for FLC was only two-fold higher than the value of 10 ± 1.4 μM obtained for strain BRE expressing AfCYP51B-6×His and 7-fold higher than strain ADΔΔ expressing the native ScCyp51. The MIC_80_ values obtained for strain T289A with VT-1161 and DCZ were each within 2- to 3-fold of those for the ADΔΔ and BRE strains. These 26- and 64-fold greater susceptibilities compared with the ARE strain are consistent with T289 in AfCYP51A, conferring high-level resistance to these compounds as well as FLC. In contrast, the susceptibility of strain T289A to PCZ increased by only 40% compared to strains ARE and ADΔΔ, probably reflecting the relative expression of each construct.

### 3.6. Difluorophenyl Substituents Differentially Affect the FLC Susceptibilities of AfCYP51 Isoforms and in Response to the AfCYP51A Mutations T289A and I301T

The relative importance of the 2- and 4-fluorines of the difluorophenyl head group of FLC, assessed by testing FLC derivatives with no head group fluorine(s) (MCC7915) or retaining one fluorine (MCC7916 4-fluorine, MCC7917 2-fluorine), is shown in Table 3. The MIC values obtained for the BRE strain show that the absence of both fluorines reduced susceptibility 50-fold, the absence of the 4-fluorine reduced susceptibility 10-fold, while the loss of the 2-fluorine reduced susceptibility 7-fold, consistent with each fluorine contributing independently to binding in the AfCYP51B active site. For the ARE strain, the absence of both fluorines reduced susceptibility 2.5-fold compared to FLC. This was maintained in the absence of the 2-fluorine but reduced 3.5-fold in the absence of the 4-fluorine, indicating that the 4-fluorine is the major determinant in FLC resistance. The AfCYP51A T289A mutation imparted improved susceptibility to the 4-fluorine derivative (~4-fold), and the I301T mutation conferred weaker improvement comparable to that obtained with the derivative lacking both fluorines (~2-fold). These data are consistent with the phenyl group, contributing weakly to binding in the AfCYP51A active site, while both fluorines affect FLC binding more strongly. Importantly, the 4-fluorine appears to weaken binding to AfCYP51A due to its interaction with T289.

Replacement of the 2,4-difluorophenyl group of FLC with a 2,5-difluorophenyl group was used to gauge whether a readily prepared congener of FLC would be effective against *A. fumigatus*. Unfortunately, the 2,5-difluorophenyl congener gave significantly (~3-fold) reduced susceptibility compared to FLC for strains ARE and I301T and >6-fold for strains BRE and T289A. These susceptibilities mimicked those for MCC7917 (FLC with only the 4-fluoro substituent), suggesting that the 5-fluoro group is sufficiently close to helix I for it to confer even lower susceptibility than the 4-fluoro group. RVC and IVC, which contain a 2,4-difluorophenyl and a 2,5-difluorophenyl group, respectively, have midlength tails that interact with the CYP51 substrate entry channel. The susceptibilities of CYP51A and AfCYP51B to RVC and IVC were strong (MIC_80_ < 0.0156 μM) and, like VCZ, strain ARE was 2-fold less susceptible compared to BRE, and these responses were reversed by both the AfCYP51A T289A or I301T mutations.

### 3.7. Acquired Azole Resistance Conferred by the AfCYP51A Mutations Y121F and Y121F T289A

#### 3.7.1. Phenotypes Conferred by Expression of AfCYP51A Mutants in *S. cerevisiae*

The MIC_80_ value of strain AfCYP51A Y121F for FLC (263 ± 31.6 μM) was not statistically different from strain ARE. This result is consistent with the conferral of intrinsic FLC resistance on AfCYP51A by T289 (Table 2). Strains Y121F and ARE also showed similar high-level resistance to VT-1161 and DCZ. As expected from previous phenotypic and structural studies of ScCYP51-6×His and ScCYP51-6×His Y140F strains [20], the AfCYP51A Y121F strain was also highly resistant to VCZ (MIC_80_ = 2.07 ± 0.21 μM), showing 8-fold greater resistance than ARE (MIC_80_ = 0.27 ± 0.03 μM). In contrast, the T289A and I301T strains had MIC_80_ values for VCZ of <0.12 μM that were comparable to those of the ADΔΔ and ADLS strains. The CYP51A Y121F strain also showed 3-fold and 6-fold increases in resistance to IVC and RVC compared to strain ARE (Table 3).

The expression of AfCYP51A Y121F T289A conferred extremely high resistance to FLC despite a very modest (~1.5-fold) increase in the recombinant protein expression compared to the ARE strain (Figure 7). The MIC_80_ value of 1060 ± 60 μM was 5-, 4-, 53-, and 106-fold greater than the corresponding values for the ARE, Y121F, T289A, and BRE strains. The Y121F T289A strain barely modified VCZ resistance compared to the Y121F strain and only showed a 4-fold increase in resistance compared to the ARE, T289A, and BRE strains. The double mutation gave a several-fold increase in resistance to VT-1161 and DCZ compared to the ARE, Y121F, T289A, and BRE strains. In contrast, the double mutation did not confer resistance to PCZ, while the resistance to IVC and RVC due to the CYP51A Y121F mutation was unaffected by the addition of the T289A mutation. These data indicate that FLC, VT-1161, and DCZ have similar T289-dependent interactions with the AfCYP51 active site. VCZ binding is most strongly affected by Y121 and more modestly by T289, while PCZ binding is essentially unaffected by either residue.

#### 3.7.2. Properties of Affinity-Purified AfCYP51A and AfCYP51B

**Figure 8:**
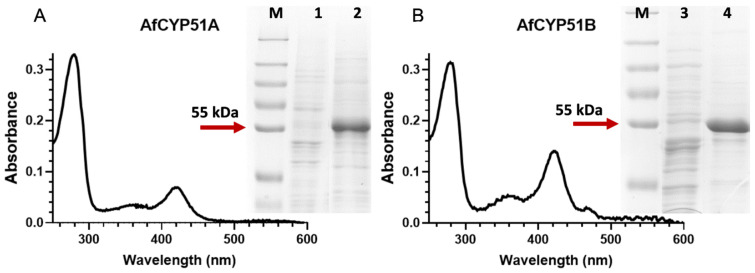
Absolute absorbance spectrum and Coomassie blue-stained SDS-PAGE gel (8% acrylamide) of Ni-NTA-purified AfCYP51A-6×His (**A**) and AfCYP51B-6×His (**B**). Protein fractions (25 μg) were separated by SDS-PAGE to assess the quality of each sample. (M) PageRuler™ Plus prestained protein standards, (1) crude membrane protein from strain A, (2) AfCYP51A-6×His-purified protein, (3) crude membrane protein from strain B, (4) AfCYP51B-6×His-purified protein.

AfCYP51-6×His proteins were partially purified from crude membrane preparations using DM solubilisation and Ni-NTA affinity chromatography (Figure 8). The absolute absorbance spectrum showed a heme peak at 419 nm for AfCYP51A-6×His and a heme peak at 422 nm for AfCYP51B-6×His (Figure 8). The ratios of A_419nm_/A_280nm_ for AfCYP51A and A_422nm_/_A280nm_ for AfCYP51B proteins were 0.21 and 0.45, respectively, consistent with the SDS-PAGE analysis. SDS-PAGE of the affinity-purified fractions confirmed that the AfCYP51B was recovered at about twice the purity of AfCYP51A.

**Figure 9:**
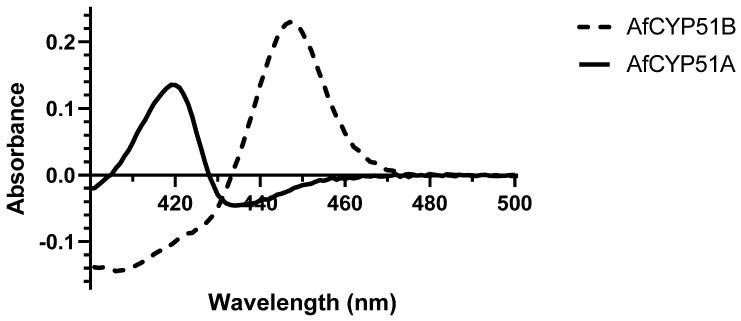
Carbon monoxide difference spectrum of Ni affinity-purified AfCYP51A-6×His (continuous line) and AfCYP51B-6×His (dash line).

The difference spectrum for carbon monoxide binding to dithionite-reduced AfCYP51B-6×His gave a peak at 447 nm and a trough at 406 nm (Figure 9). The absence of a peak at 420 nm indicated that the preparation was fully active. In contrast, the difference spectrum for AfCYP51A-6×His gave a peak at 419 nm and a trough at 436 nm. This result, replicated in three further experiments, may indicate significant misfolding of AfCYP51A-6×His in this preparation in the absence of a ligand.

#### 3.7.3. Type I Binding of Lanosterol and Eburicol by AfCYP51A and AfCYP51B

**Figure 10:**
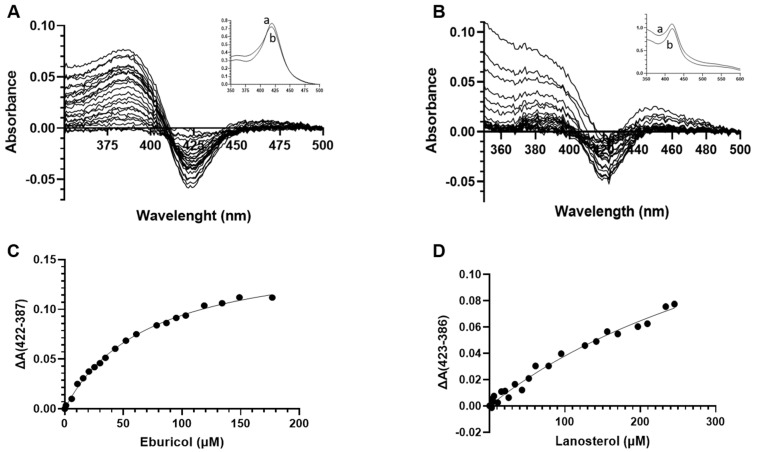
Type I binding of (**A**) eburicol and (**B**) lanosterol by AfCYP51B-6×His (5 μM) and related saturation curves for (**C**) eburicol and (**D**) lanosterol. Absolute absorbance spectra are shown in the thumbnails; (a) reference, (b) complex with substrate.

Type I binding of substrates lanosterol and eburicol was carried out for Ni-NTA affinity-purified AfCYP51-6×His proteins. Displacement of the heme-coordinated water molecule during type I substrate binding causes a low to high spin shift of the ferric P450 heme iron and, consequently, a blue shift in the Soret band maximum. A blue shift spin state change was recorded for AfCYP51B substrates. The peak characteristic of type I binding spectra was obtained at 386 and 387nm for lanosterol and eburicol, respectively (Figure 10). A corresponding trough was observed at 423 nm for lanosterol binding and 422 nm for eburicol. Saturation curves (Figure 10C,D) were graphed, and binding parameters were evaluated by calculating the difference in absorbance between the peak and trough for each substrate. Michaelis–Menten binding parameters were calculated using GraphPad Prism 9.2.0 Software (GraphPad Software, Boston, MA, USA), with the binding titrated against substrate concentration. The *K_s_* value for eburicol binding was 71.02 μM, and that for lanosterol was 476.7 μM, indicating lanosterol bound to AfCYP51B with approximately 6-fold lower affinity than eburicol (Table 4). The binding maximum absorbance values estimated for eburicol and lanosterol were 0.16 and 0.22, respectively.

AfCYP51A-6×His preparations did not give a type I binding response at concentrations up to 300 μM of lanosterol and eburicol (Figure S12).

#### 3.7.4. Type II Binding of Azole Drugs by AfCYP51A and AfCYP51B

**Figure 11:**
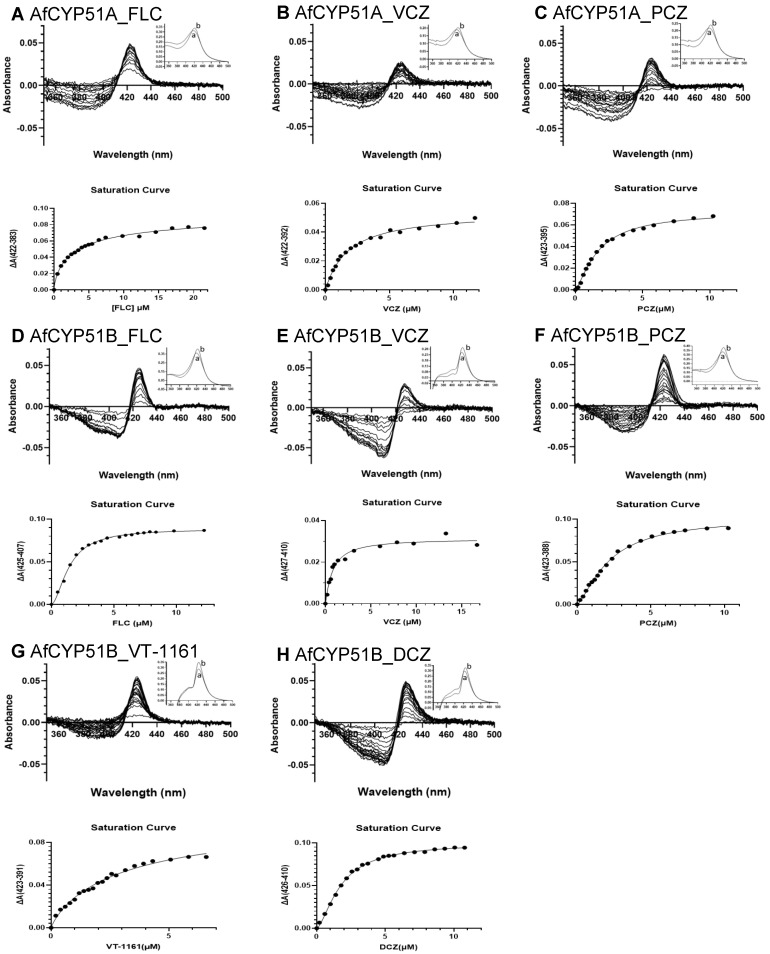
Type II binding of azole drugs to AfCYP51A-6×His and AfCYP51B-6×His. Each azole drug was titrated against 1 μM enzyme. The saturation curve for each azole drug was fitted using the Hill equation. The type II difference spectra and saturation curve of AfCYP51A were determined for the binding of FLC (**A**), VCZ (**B**), and PCZ (**C**). The type II difference spectra and saturation curve of AfCYP51B were determined for the binding of FLC (**D**), VCZ (**E**), PCZ (**F**), VT-1161 (**G**), and DCZ (**H**). The binding parameters obtained are presented in Table 5. Absolute absorbance spectra are shown in the thumbnails. (a) Reference, (b) complex with inhibitor.

Type II binding of FLC, VCZ, and PCZ were determined for AfCYP51A-6×His and AfCYP51B-6×His. Type II binding of VT-1161 and DCZ was also determined for AfCYP51B-6×His. The binding of each triazole to the AfCYP51-6×His preparations gave a red shift in the Soret peak. The resultant difference spectra and azole saturation curves are shown in Figure 11. The ΔAmax and *K_d_* values obtained from these data are presented in Table 5.

Type II binding by AfCYP51A and AfCYP51B showed narrow peaks for FLC, VCZ, and PCZ, but AfCYP51A gave troughs that were wider and more blue-shifted for VCZ and especially for FLC. The ΔAmax value of FLC with AfCYP51A (ΔAmax = 0.10) or AfCYP51B (ΔAmax = 0.09) were very similar. In contrast, the AfCYP51A *K_d_* (3.23 μM) for FLC was 2.26-fold higher than for AfCYP51B (1.43 μM). These data show that Ni-NTA-purified AfCYP51A had a significantly lower affinity for FLC than AfCYP51B.

The type II ΔAmax values for VCZ binding were 0.05 and 0.03 for AfCYP51A and AfCYP51B, respectively. The binding of VCZ by AfCYP51A gave a peak at 422 nm and a trough minimum at 392 nm. The binding of VCZ by AfCYP51B gave a slightly more red-shifted profile, with a peak at 427 nm and a narrow trough with a minimum of 410 nm. PCZ binding by AfCYP51A and AfCYP51B gave ΔAmax values of 0.07 and 0.10, respectively. A peak at 423 nm was observed for both enzymes with PCZ, but AfCYP51A and AfCYP51B gave different trough minima at 395 nm and 388 nm, respectively.

Although all the azole drugs tested (including VT-1161 and DCZ tested with AfCYP51B only) bound strongly to both AfCYP51A and AfCYP51B (*K_d_* < 3.3 μM), only the binding of VCZ to AfCYP51B showed tight 1-1 binding indicated by a dissociation constant (*K_d_* = 0.8 μM) lower than the 1 μM enzyme concentration assayed [36]. While VCZ showed weaker binding to AfCYP51A (*K_d_* = 1.5 μM), PCZ showed comparable affinities (*K_d_* = 1.7 and 2.1 μM for AfCYP51A and AfCYP51B, respectively). The respective Hill coefficients of 0.87 and 1.75 for FLC binding to AfCYP51A and AfCYP51B were consistent with significantly different binding modes. However, we cannot exclude the possibility that the “significant misfolding” of affinity-purified AfCYP51A affected the measurement of azole binding significantly.

## 4. Discussion

### 4.1. Constructs Needed for Functional Expression of AfCYP51s in S. cerevisiae

The functional expression of full-length recombinant *A. fumigatus* CYP51A and CYP51B in *S. cerevisiae* strain ADΔΔ was achieved by constructing the ARE and BRE strains, respectively. Full-length codon-optimised AfCYP51A-6×His and AfCYP51B-6×His were constitutively co-expressed together with a cognate NADPH–cytochrome P450 reductase (AfCPRA2) to generate appropriate redox capacity plus a cognate sterol C24-methyltransferase (AfERG6) enzyme to provide the substrate eburicol. In contrast to the expression of AfCPRA2-6×His, no overexpressed protein band was detected in crude membrane preparations obtained from strains transformed with *AfCPRA1-6*×*His*. A fragment inside the ORF of *AfCprA1-6*×*His* was successfully amplified from cDNA preparations obtained by reverse transcriptase PCR from these transformants. Collectively, the results show that the *AfCprA1-6*×*His* construct was transcribed but not translated.

Both eburicol and lanosterol have been proposed as substrates for *A. fumigatus* and other moulds [37]. We have demonstrated (Figure 6) that the *S. cerevisiae* host used in the present study did not produce detectable amounts of eburicol, even when treated with the azole drug VCZ [36]. The ScErg6 enzyme does not convert lanosterol to eburicol and instead catalyses the conversion of zymosterol to fecosterol (Figure 1). We hypothesised that functional expression of the *AfErg6* gene in *S. cerevisiae* would result in the synthesis from lanosterol of the eburicol required for AfCYP51A and AfCYP51B function. This idea was validated by constructing the viable ARE and BRE strains in which the native *ScCyp51* gene of the AR and BR strains was replaced with recombinant *AfErg6-FLAG* (Figure S5). Deletion of the native *ScCyp51* gene was not sustainable, but its substitution by *AfERG6*, and hence the eburicol-dependent growth of the ARE and BRE strains, demonstrated that the AfERG6, AfCYP51A, and AfCYP51B enzymes are functional and that both ARE and BRE use eburicol. Of the yeast-based strains tested, the detection of eburicol in ARE and BRE but not ADΔΔ and Y2300 (which both express *ScCyp51*), plus its enhanced levels in response to growth rate inhibiting concentrations VCZ confirmed our prediction from modelling studies that eburicol is the substrate of AfCYP51A and AfCYP51B. Furthermore, CYP51B was found to bind eburicol with an affinity at least 6-fold greater than for lanosterol.

The importance of a cognate NADPH–cytochrome P450 reductase in the overexpression of functional CYP51 enzymes from a range of fungal species in *S. cerevisiae* has been demonstrated previously for the *C. albicans* and *C. glabrata* CYP51s [24]. However, it is unclear whether the AfCYP51-dependent viability of strains ARE and BRE requires just AfERG6 or a combination of AfERG6 and AfCPRA2 overexpression. The essentially identical azole susceptibility phenotypes of the A and AR strains, and the B and BR strains, are consistent with the native yeast enzyme rather than the recombinant enzymes providing the biosynthetic activity required for ergosterol biosynthesis and may indicate AfCPRA2 is not a major player. However, its inclusion may assist in vitro assays of AfCYP51s using the NADPH–cytochrome P450 reductase substrate NADPH and the artificial CYP51 substrate 7-benzloxymethyloxy-3-cynanocoumarian (BOMCC) [38]. These ideas can be tested in the future by deleting *AfCprA2* from the ARE and BRE strains.

### 4.2. Innate Resistance to FLC in A. fumigatus Is Primarily Due to AfCYP51A T289

Azole susceptibility determinations showed that both AfCYP51A-6×His and AfCYP51B-6×His are functional in strains ARE and BRE and hence complement deletion of the endogenous *ScCyp51* gene once eburicol is provided. The phenotypes of these strains and the AfCYP51A T289A mutant demonstrated that AfCYP51A but not AfCYP51B confers high-level resistance to FLC, both fluorines of the FLC difluorophenyl head group contribute to the susceptibility of AfCYP51B, but the 4-fluorine dominates the FLC resistance of AfCYP51A. Despite strain ARE being only 2-fold more resistant to IVC than strain BRE, resistance to FLC was not circumvented by the 2,5-difluorine derivative of FLC. Furthermore, susceptibility was substantially restored by the AfCYP51A T289A substitution and less so by the AfCYP51B I301T mutation. These results demonstrate that although the 2-fluorine contributes to susceptibility, the susceptibilities conferred by 4- and 5-fluorine substituents are both strongly affected by AfCYP51A T289. Furthermore, in comparison to AFCYP51B and ScCYP51, AfCYP51A conferred high-level resistance to DCZ and VT-1161, weak resistance to VCZ, and did not confer resistance to PCZ or the control echinocandin antifungal MCF. Consistent with the intrinsic resistance of AfCYP51A to FLC, Type II binding showed AfCYP51B (*K_d_* = 1.43 μM) has a significantly higher affinity for FLC than AfCYP51A (*K_d_* = 3.23 μM). AfCYP51B also showed a significantly higher affinity for VCZ (*K_d_* = 0.80 μM) than AfCYP51A (*K_d_* = 1.52 μM), while the affinities of both preparations for PCZ (*K_d_* = 2.15 and 1.72 μM, respectively) are almost equivalent. Hill coefficients in the range of 1–2 for VCZ and PCZ with the AfCYP51 enzymes suggest weak positive co-operative allosteric binding of these drugs. In our experience, affinity-purified CYP51 preparations from *S. cerevisiae* and a range of fungal pathogens behave as monomers on size exclusion chromatography, and the AfCYP51 isoforms behave similarly. This implies that the positive cooperativity indicated by Hill coefficients of 1–2 involves multiple drug–enzyme interactions rather than enzyme–enzyme interactions. The Hill coefficients of 0.72 and 1.75 for FLC binding to AfCYP51A and AfCYP51B, respectively, appear consistent with significantly different binding modes, i.e., the binding of FLC in the AfCYP51A active site involves fewer productive drug–enzyme interactions than with AfCYP51B. In addition to the direct interaction of their azole groups with the heme, FLC, DCZ, VT-1161, VCZ, and PCZ should all bind in the sterol 14α-demethylase active site coordinated to the heme in a region that is also occupied by the substrate. The FLC difluorophenyl group and the even more bulky DCZ diclorophenoxyphenyl head group are expected to have intimate interactions almost adjacent to the kink in helix I (at AfCYP51A T289 and AfCYP51B A303) and with phenylalanine (the conserved F115 in CYP51A and F130 in CYP51B) in the adjacent BC-loop. In addition, FLC, VT-1161, and VCZ each interact via a water-mediated hydrogen bond network with Y121 in the AfCYP51A BC-loop. FLC and VCZ may also interact via compound-specific, water-mediated hydrogen bond networks with the internal loop extending from helix KK’, with VCZ likely to interact more strongly than FLC. However, VT-1161 is not involved in a similar stabilising interaction and, in particular, appears unlikely to form a strong hydrogen bond network with the conserved H365 imidazole in AfCYP51A that is equivalent to H381 in *S. cerevisiae* CYP51 [39]. The triazole and difluorophenyl head group of PCZ occupies a similar space to the head groups of VCZ and FLC. Its oxolane linker prevents interaction with Y121, while the long tail of PCZ makes numerous potentially stabilising contacts with the enzyme as it extends to the mouth of the substrate entry channel.

Successful overexpression of functional recombinant AfCYP51A T289A and AfCYP51 I301T mutants derived from the ARE strain provides further insight into azole binding and confirms the role of the CYP51A T289 substitution in the conferral of FLC resistance on *A. fumigatus*. Modelling of AfCYP51A indicated that the size and polarity of T289 in helix I are likely to interfere with the binding of FLC in the active site, while the I301 is too distant from FLC to interact with it directly. Our data help explain the even lower affinity of AfCYP51A for DCZ, the reduced affinity for VT-1161, and the slightly reduced affinity for VCZ in comparison with AfCYP51B. As predicted, the substitution of threonine with the significantly smaller, non-polar amino acid alanine in AfCYP51A T289A to match structurally aligned AfCYP51B resulted in increased susceptibility to these azoles. Also, as expected, the AfCYP51A T289A mutation has almost no effect on PCZ susceptibility, with the susceptibilities of strains ARE and BRE equivalent to those of the parental ADΔΔ strain and the ADLS strain. The AfCYP51A I301T mutant showed a modest increase in susceptibility to FLC (3-fold) compared to its ARE parental strain, and this was much less than that found with the AfCYP51A T289A mutant (11-fold). The increased susceptibility due to the AfCYP51 I301T mutation may be due to reduced helix stability conferred by the threonine (helix propensity of I = 0.41, T = 0.66) as well as the threonine hydroxyl group interacting with a peptide amide a turn of helix I away (compare PDB: 4WZM and 5ESM). In contrast, the AfCYP51A T289A mutation will increase helix stability significantly (helix propensity of A = 0) and potentially reduce flexibility one turn away from the conserved glycine, marking the central kink in helix I. Measurements of sterol composition, particularly lanosterol and eburicol contents, are consistent with the I301T mutation increasing AfCYP51A catalytic efficiency, while the T289A mutation does not.

The susceptibilities of ARE and BRE to PCZ are greater than for the ADLS strain, which expresses significantly higher levels of CYP51. It is suggested that the levels of PCZ susceptibility detected are consistent with the relative expression of the AfCYP51 and ScCYP51 proteins in these constructs. The resistance to VCZ of the ARE and BRE strains is presumably due to improved functional expression of both AfCYP51A and AfCYP51B. We suggest that VCZ binding to AfCYP51A and AfCYP51B is of sufficient high affinity to eliminate competition with eburicol, whereas FLC, DCZ, and VT-1161 are less effective in this regard, with ARE showing several-fold higher levels of eburicol than BRE. Our findings also support the continued use of VCZ as a first-line treatment for invasive aspergillosis, with PCZ and possibly IVC and RVC best kept as reliable salvage therapy for VCZ-resistant infections unless drug interactions limit their use.

### 4.3. Acquired Resistance of AfCYP51A to Azole Drugs

*S. cerevisiae* CYP51 Y140, which structurally aligns with AfCYP51A Y121, is a key amino acid in the conferral of resistance to the short-tailed azoles FLC and VCZ and the medium-tailed tetrazole VT-1161 [28,39]. The present study has shown that the AfCYP51A Y121F and Y121F T289A mutations confer 8- and 11-fold increases in resistance to VCZ, respectively. Resistance also increased to VT-1161 and FLC despite the AfCYP51A T289A mutation alone conferring increased susceptibility to FLC (11-fold), VCZ (2.5-fold), and VT-1161 (21-fold). The lack of the tyrosine hydroxyl in AfCYP51A Y121F means that the water-mediated hydrogen bond network involving the tertiary alcohol of FLC, VCZ, or VT-1161 is not formed, and the affinity of the enzyme for these drugs is significantly reduced [28]. As expected, the AfCYP51A Y121F mutation had no effect on PCZ susceptibility because this drug lacks the tertiary alcohol, and its long tail has multiple interactions with the substrate entry channel. In contrast to the findings with ScCYP51 Y140F [40], the AfCYP51A Y121F mutation increased susceptibility to DCZ 2-fold, the AfCYP51A Y121F T289A combination reduced susceptibility 1.5-fold, while the AFCYP51A T289A mutation increased susceptibility 64-fold. Although DCZ lacks tertiary alcohol, the Y121F mutation in the BC-loop also prevents the formation of a hydrogen bond with its neighbouring heme ring propionate. We speculate that this modification allows the BC-loop to adjust its conformation and make more space for the dichlorophenyloxyphenyl group of DCZ adjacent to T289 [41]. While the T289A mutation is expected to increase active site volume and reduce flexibility at helix I that enables improved binding of the DCZ in the AfCYP51A active site, additional conformational flexibility imparted on the BC-loop and the heme by the Y121F mutation might also confer preferential binding of eburicol that more effectively excludes DCZ. The AfCYP51A Y121F and AfCYP51A Y121F T289A mutations confer significant resistance to VCZ (>8-fold) and a lesser (3- to 5-fold) increase in resistance to the extremely potent and midlength-tailed drugs RVC and IVC, but neither mutation confers significant resistance to the longer-tailed PCZ. RVC and IVC may therefore be expected to be active against both wild-type and the increasingly clinically important TR36/Y121F/T289A mutants of *A. fumigatus* when delivered using prodrugs (e.g., IVC as isavuconazolium sulphate and RVC as fosravuconazole) and might provide better results during salvage therapy than PCZ. High-resolution crystal structures of the AfCYP51A enzyme and its mutants in complex with azole drugs are now needed to complement our existing structures of ScCYP51 in complex with therapeutic azoles and DCZ stereoisomers [40]. These will provide a better understanding of the conformational responses in the BC-loop and helix I to the Y121F and T289A mutations and more fully probe the molecular basis of the susceptibility of AfCYP51A to azole drugs and agrochemicals. In this context, it is relevant to note that the CYP51A Y121F mutant has yet to be detected in the clinic.

Alignment with obtusifoliol 14α-demethylases from dicotyledonous plants (e.g., tobacco; UniPortKB_Q8GVD5, garden pea; UniPortKB_A0A0E3VN01, wild strawberry; UniPortKB_X5J4V7, and petunia; UniPortKB_A0PFU3) and monocotyledon plants (e.g., wheat; UniPortKB_P93596 and sorghum UniPortKB_P93846) shows that the residues equivalent to Y121 and T289 in AfCYP51A are intrinsically substituted with phenylalanine and alanine, respectively, and another alanine resides immediately downstream of that alanine (Figure 2). As plants are not expected to be affected by azole agrochemicals such as DCZ, the similarity of AfCYP51A Y121F T289A to plant CYP51s is consistent with the ability of AfCYP51A Y121F T289A to confer high-level resistance to FLC and DCZ. Compared with its CYP51 F1 isoform, the *Rhizopus arrhizus* CYP51 F5 isoform also contains a plant-like set of structurally aligned substitutions (Y129F V291A) that give rise to innate resistance to FLC plus VCZ, IVC, and DCZ. However, other features must be responsible for the innate resistance of human CYP51 to the triazole antifungal drugs.

## 5. Conclusions

This report demonstrates the eburicol-dependence of AfCYP51 isoforms and that the AfCYP51A and not the AfCYP51B isoform is responsible for the innate FLC resistance of *A. fumigatus*. Mutational studies show that the AfCYP51A T289 residue in helix I is primarily responsible for the FLC resistance, with a minor contribution due to I301. This molecular-level insight explains why VCZ and PCZ, but not FLC, provide effective antifungal treatments for *A. fumigatus* infections. When AfCYP51A TR46/Y121F/T289A mutations are acquired, PCZ and efficiently delivered IVC and RVC can be expected to provide effective salvage therapy if drug interactions can be avoided.

## Figures and Tables

**Table 1:** The strains prepared for this study.

Strain Name	Location of Recombinant Gene(s) in Host Cell (ADΔΔ)
**A**	***PDR5***::*AfCyp51A*-*6×His*
**B**	***PDR5***::*AfCyp51B*-*6×His*
**AR**	***PDR5***::*AfCyp51A-6×His*, ***PDR15***::*AfCprA2*-*6×His*
**BR**	***PDR5***::*AfCyp51B*-*6×His*, ***PDR15***::*AfCprA2*-*6×His*
**ARE**	***PDR5***::*AfCyp51A*-*6×His*, ***PDR15:***:*AfCprA2*, ***ΔScCyp51***::*AfErg6-FLAG*
**Y121F**	***PDR5***::*AfCyp51A*-*6×His Y121F*, ***PDR15***::*AfCprA2*, ***ΔScCyp51***::*AfErg6-FLAG*
**T289A**	***PDR5***::*AfCyp51A*-*6×His T289A*, ***PDR15***::*AfCprA2*, ***ΔScCyp51***::*AfErg6-FLAG*
**Y121F T289A**	***PDR5***::*AfCyp51A*-*6×His Y121F T289A*, ***PDR15***::*AfCprA2*, ***ΔScCyp51***::*AfErg6-FLAG*
**I301T**	***PDR5***::*AfCyp51A*-*6×His I301T*, ***PDR15***::*AfCprA2*, ***ΔScCyp51***::*AfErg6-FLAG*
**BRE**	***PDR5***::*AfCyp51B*-*6×His*, ***PDR15***::*AfCprA2*, ***ΔScCyp51***::*AfErg6-FLAG*
**ADLS**	***PDR5***::*ScCyp51*-*6×His*, ***ΔScCyp51***::*His1*

**Table 2:** MIC_80_ values for control strains ADΔΔ and ADLS and recombinant strains overexpressing AfCYP51 isoforms.

	ScCYP51		AfCYP51A			AfCYP51A Mutation				AfCYP51B		
	ADΔΔ	ADLS	A	AR	ARE	Y121F	T289A	Y121F T289A	I301T	B	BR	BRE
**FLC**	3.3 ± 0.2	8.0 ± 0.2	3.8 ± 0.2	6.5 ± 0.7	227 ± 18.5	263 ± 31.6	21.3 ± 2.7	1060 ± 60	77.2 ± 5.9	3.8 ± 0.2	2.9 ± 0.4	10.0 ± 1.4
**VCZ**	0.06 ± 0.00	0.17 ± 0.03	0.08 ± 0.01	0.11 ± 0.02	0.27 ± 0.03	2.07 ± 0.21	0.11 ± 0.00	2.86 ± 0.22	0.11 ± 0.00	0.08 ± 0.01	0.07 ± 0.01	0.21 ± 0.02
**PCZ**	0.10 ± 0.01	0.29 ± 0.04	0.21 ± 0.01	0.19 ± 0.02	0.10 ± 0.01	0.06 ± 0.01	0.06 ± 0.00	0.11 ± 0.01	0.09 ± 0.02	0.25 ± 0.03	0.17 ± 0.02	0.10 ± 0.01
**VT-1161**	0.01 ± 0.00	0.06 ± 0.00	0.03 ± 0.00	0.06 ± 0.01	0.43 ± 0.05	0.53 ± 0.02	0.02 ± 0.00	1.07 ± 0.05	0.14 ± 0.04	0.05 ± 0.01	0.03 ± 0.00	0.06 ± 0.01
**DCZ**	<0.01	0.04 ± 0.01	0.01 ± 0.00	.07 ± 0.01	0.64 ± 0.04	0.37 ± 0.02	<0.01	0.92 ± 0.07	0.35 ± 0.06	0.01 ± 0.00	<0.01	<0.01
**MCF**	0.22 ± 0.02	0.22 ± 0.01	0.19 ± 0.03	0.17 ± 0.03	0.22 ± 0.02	0.24 ± 0.02	0.27 ± 0.00	0.21 ± 0.01	0.28 ± 0.01	0.18 ± 0.02	0.17 ± 0.03	0.14 ± 0.01

MIC values are in μM. Errors are provided as SD values.

**Table 3:** MIC_80_ values for CYP51A mutants against FLC, FLC derivatives, and azole drugs.

Compound	Strains					
	BRE	ARE	ARE T289A	ARE I301T	ARE Y121F	ARE Y121F T289A
**FLC** **2,4-difluorophenyl**	3.8 ± 0.9	151 ± 66	12 ± 2	65 ± 14	157 ± 33	725 ± 65
**MCC7915 FLC** **Phenyl**	223 ± 79	427 ± 23	248 ± 23	278 ± 45	180 ± 11	219 ± 29
**MCC7916 FLC** **2-fluorophenyl**	21 ± 3	120 ± 40	52 ± 6	51 ± 6	80 ± 17	198 ± 41
**MCC7917 FLC** **4-fluorophenyl**	38 ± 11	411 ± 30	103 ± 11	257 ± 37	179 ± 9	242 ± 41
**MCC8768 FLC** **2,5-difluorophenyl**	25 ± 8	354 ± 76	106 ± 28	211 ± 21	324 ± 109	1025 ± 148
**RVC** **2,4-difluorophenyl**	0.0038 ± 0.0008	0.0088 ± 0.0023	0.0032 ± 0.0015	0.0034 ± 0.0015	0.047 ± 0.005	0.033 ± 0.006
**IVC** **2,5-difluorophenyl**	0.0109 ± 0.003	0.021 ± 0.007	0.0102 ± 0.0057	0.0119 ± 0.0028	0.071 ± 0.014	0.110 ± 0.011
**PCZ** **2,4-difluorophenyl**	0.060 ± 0.037	0.050 ± 0.017	0.043 ± 0.019	0.055 ± 0.001	0.025 ± 0.003	0.101 ± 0.017

MIC_80_ values are in μM. Errors are provided as SD values.

**Table 4:** Substrate binding parameters for AfCYP51B-6×His type I binding of eburicol and lanosterol.

	Eburicol	Lanosterol
ΔAmax	0.161	0.219
*Ks* (μM)	71.02	476.7

Type I binding paramters were calculated using the Michaelis–Menten equation.

**Table 5:** Azole binding properties of affinity-purified AfCYP51A and AfCYP51B.

	Azole	ΔAmax	λ_peak_	λ_trough_	Hill Number	*K_d_* (μM)
AfCYP51A	FLC	0.10	422	383	0.72	3.23 ± 1.13
	VCZ	0.05	422	392	1.10	1.52 ± 0.30
	PCZ	0.07	423	395	1.35	1.72 ± 0.19
AfCYP51B	FLC	0.09	425	407	1.75	1.43 ± 0.07
	VCZ	0.03	427	410	1.12	0.80 ± 0.31
	PCZ	0.10	423	388	1.41	2.15 ± 0.20
	VT-1161	0.11	423	391	0.87	3.23 ± 2.50
	DCZ	0.10	426	410	1.55	1.76 ± 0.08

Type II binding parameters were calculated using the Hill equation.

## Data Availability

The original contributions presented in the study are included in the article/Supplementary Materials, further inquiries can be directed to the corresponding author.

## Supplementary Materials

The following supporting information can be downloaded at https://www.mdpi.com/article/10.3390/jof10120820/s1, Table S1: Azole susceptibilities of ScCYP51 and ScCYP51 T322I expressed in *S. cerevisiae*; Table S2: Yeast strains used in this study; Table S3: DNA oligonucleotide primers used in this study; Table S4: Mutagenic primers used to prepare modified AfCYP51 isoforms; Figure S1: Impact of ScCYP51 T322 and the T322I mutation on helix I; Figure S2: Strategies for preparation of recombinant yeast strains; Figure S3: Hexahistidine tag removal from *AfCprA2-6×His* in the *S. cerevisiae PDR15* locus; Figure S4: Scheme for replacement of the native *S. cerevisiae Erg11* (*ScErg11*) with codon-optimised *AfErg6* with a C-terminal *FLAG* tag; Figure S5: Strategy used to create recombinant *S. cerevisiae* constructs expressing AfCYP51A or AfCYP51B, AfCPR, and AfERG6; Figure S6: Recombinant PCR-based introduction of single or double amino acid mutations into AfCYP51A; Figure S7: Validation of recombinant *S. cerevisiae* strains. A. DNA sequence analysis of recombinant *AfCyp51* genes. B. Mass spectrometry analysis of tryptic fragments from SDS-PAGE separated bands containing putative recombinant AfCYP51A or AfCYP51B; Figure S8: Synthesis schemes for congeners of fluconazole (FLC); Figure S9: FPLC, mass spectrometry, and NMR analysis of congeners of FLC; Figure S10: Superposition of lanosterol and docking of lanosterol and eburicol in a homology model of AfCYP51A obtained using HsCYP51 (PDB ID: 6UEZ) as a template; Figure S11: Response of host and recombinant *S. cerevisiae* strains to azole drugs and MCF in agarose diffusion assays; Figure S12: Type I difference spectra for the binding of eburicol (A) and lanosterol (B) to Ni-NTA affinity-purified AfCYP51A-6×His. References [15,23,28] are cited in the Supplementary Materials.

## References

[1] Chakrabarti A., Kaur H. (2016). Allergic aspergillus rhinosinusitis. J. Fungi.

[2] Greenberger P.A. (2002). Allergic bronchopulmonary aspergillosis. J. Allergy Clin. Immunol..

[3] Tatara A.M., Mikos A.G., Kontoyiannis D.P. (2016). Factors affecting patient outcome in primary cutaneous aspergillosis. Medicine.

[4] Kosmidis C., Denning D.W. (2015). The clinical spectrum of pulmonary aspergillosis. Thorax.

[5] Kanaujia R., Singh S., Rudramurthy S.M. (2023). Aspergillosis: An Update on Clinical Spectrum, Diagnostic Schemes, and Management. Curr. Fungal Infect. Rep..

[6] Singh N., Paterson D.L. (2005). Aspergillus infections in transplant recipients. Clin. Microbiol. Rev..

[7] Warrilow A.G., Melo N., Martel C.M., Parker J.E., Nes W.D., Kelly S.L., Kelly D.E. (2010). Expression, purification, and characterization of *Aspergillus fumigatus* sterol 14-α demethylase (CYP51) isoenzymes A and B. Antimicrob. Agents Chemother..

[8] Snelders E., Camps S.M., Karawajczyk A., Schaftenaar G., Kema G.H., van der Lee H.A., Klassen C.H., Melchers W.J.G., Verweij P.E. (2012). Triazole fungicides can induce cross-resistance to medical triazoles in *Aspergillus fumigatus*. PLoS ONE.

[9] Warrilow A.G., Parker J.E., Price C.L., Rolley N.J., Nes W.D., Kelly D.E., Kelly S.L. (2019). Isavuconazole and voriconazole inhibition of sterol 14α-demethylases (CYP51) from *Aspergillus fumigatus* and *Homo sapiens*. Int. J. Antimicrob. Agents.

[10] Hargrove T.Y., Wawrzak Z., Liu J., Nes W.D., Waterman M.R., Lepesheva G.I. (2011). Substrate preferences and catalytic parameters determined by structural characteristics of sterol 14α-demethylase (CYP51) from *Leishmania infantum*. J. Biol. Chem..

[11] Xie J., Rybak J.M., Martin-Vicente A., Guruceaga X., Thorn H.I., Nywening A.V., Ge W., Parker J.E., Kelly S.L., Rogers P.D. (2024). The sterol C-24 methyltransferase encoding gene, erg6, is essential for viability of Aspergillus species. Nat. Commun..

[12] Monk B.C., Sagatova A.A., Hosseini P., Ruma Y.N., Wilson R.K., Keniya M.V. (2020). Fungal Lanosterol 14α-demethylase: A target for next-generation antifungal design. Biochim. Biophys. Acta Proteins Proteom..

[13] Caramalho R., Tyndall J.D., Monk B.C., Larentis T., Lass-Flörl C., Lackner M. (2017). Intrinsic short-tailed azole resistance in mucormycetes is due to an evolutionary conserved aminoacid substitution of the lanosterol 14α-demethylase. Sci. Rep..

[14] Rosam K., Monk B.C., Lackner M. (2021). Sterol 14α-Demethylase Ligand-Binding Pocket-Mediated Acquired and Intrinsic Azole Resistance in Fungal Pathogens. J. Fungi.

[15] Ruma Y.N., Keniya M.V., Monk B.C. (2022). Exploring *Cryptococcus neoformans* CYP51 and Its Cognate Reductase as a Drug Target. J. Fungi.

[16] van der Linden J.W., Snelders E., Kampinga G.A., Rijnders B.J., Mattsson E., Debets-Ossenkopp Y.J. (2011). Clinical implications of azole resistance in *Aspergillus fumigatus*, The Netherlands, 2007–2009. Emerg. Infect. Dis..

[17] Stensvold C.R., Jørgensen L.N., Arendrup M.C. (2012). Azole-resistant invasive aspergillosis: Relationship to agriculture. Curr. Fungal Infect. Rep..

[18] Snelders E., van der Lee H.A., Kuijpers J., Rijs A.J.M., Varga J., Samson R.A., Medallo E., Donders A.R.T., Melchers W.J., Verweij P.E. (2008). Emergence of azole resistance in *Aspergillus fumigatus* and spread of a single resistance mechanism. PLoS Med..

[19] Leonardelli F., Macedo D., Dudiuk C., Cabeza M.S., Gamarra S., Garcia-Effron G. (2016). *Aspergillus fumigatus* intrinsic fluconazole resistance is due to the naturally occurring T301I substitution in Cyp51Ap. Antimicrob. Agents Chemother..

[20] Sagatova A.A., Keniya M.V., Wilson R.K., Sabherwal M., Tyndall J.D., Monk B.C. (2016). Triazole resistance mediated by mutations of a conserved active site tyrosine in fungal lanosterol 14α-demethylase. Sci. Rep..

[21] Nierman W.C., Pain A., Anderson M.J., Wortman J.R., Kim H.S., Arroyo J., Berriman M., Abe K., Archer D.B., Bermejo C. (2005). Genomic sequence of the pathogenic and allergenic filamentous fungus *Aspergillus fumigatus*. Nature.

[22] Curran K.A., Morse N.J., Markham K.A., Wagman A.M., Gupta A., Alper H.S. (2015). Short synthetic terminators for improved heterologous gene expression in yeast. ACS Synth. Biol..

[23] Lamping E., Monk B.C., Niimi K., Holmes A.R., Tsao S., Tanabe K., Niimi K., Uehara Y., Cannon R.D. (2007). Characterization of three classes of membrane proteins involved in fungal azole resistance by functional hyperexpression in *Saccharomyces cerevisiae*. Eukaryot. Cell.

[24] Keniya M.V., Ruma Y.N., Tyndall J.D., Monk B.C. (2018). Heterologous expression of full-length lanosterol 14α-demethylases of prominent fungal pathogens *Candida albicans* and *Candida glabrata* provides tools for antifungal discovery. Antimicrob. Agents Chemother..

[25] Monk B.C., Tomasiak T.M., Keniya M.V., Huschmann F.U., Tyndall J.D., O’Connell J.D., Cannon R.D., McDonald J.G., Rodriguez A., Finer-Moore J.S. (2014). Architecture of a single membrane spanning cytochrome P450 suggests constraints that orient the catalytic domain relative to a bilayer. Proc. Natl. Acad. Sci. USA.

[26] Lowry O.H., Rosebrough N.J., Farr A.L., Randall R.J. (1951). Protein measurement with the Folin phenol reagent. J. Biol. Chem..

[27] Laemmli U.K. (1970). Cleavage of structural proteins during the assembly of the head of bacteriophage T4. Nature.

[28] Sagatova A.A., Keniya M.V., Wilson R.K., Monk B.C., Tyndall J.D. (2015). Structural insights into binding of the antifungal drug fluconazole to *Saccharomyces cerevisiae* lanosterol 14α-demethylase. Antimicrob. Agents Chemother..

[29] Guengerich F.P., Martin M.V., Sohl C.D., Cheng Q. (2009). Measurement of cytochrome P450 and NADPH–cytochrome P450 reductase. Nat. Protoc..

[30] Warrilow A.G., Martel C.M., Parker J.E., Melo N., Lamb D.C., Nes W.D., Kelly D.E., Kelly S.L. (2010). Azole binding properties of *Candida albicans* sterol 14-α demethylase (CaCYP51). Antimicrob. Agents Chemother..

[31] Muller C., Junker J., Bracher F., Giera M. (2019). A gas chromatography-mass spectrometry-based whole-cell screening assay for target identification in distal cholesterol biosynthesis. Nat. Protoc..

[32] Webb B., Sali A. (2016). Comparative Protein Structure Modeling Using MODELLER. Curr. Protoc. Bioinform..

[33] Hargrove T.Y., Wawrzak Z., Guengerich F.P., Lepesheva G.I. (2020). A requirement for an active proton delivery network supports a compound I-mediated C-C bond cleavage in CYP51 catalysis. J. Biol. Chem..

[34] Lescar J., Meyer I., Akshita K., Srinivasaraghavan K., Verma C., Palous M., Mazier D., Darty A., Fekkar A. (2014). *Aspergillus fumigatus* harbouring the sole Y121F mutation shows decreased susceptibility to voriconazole but maintained susceptibility to itraconazole and posaconazole. J. Antimicrob. Chemother..

[35] van der Linden J.W., Camps S.M., Kampinga G.A., Arends J.P., Debets-Ossenkopp Y.J., Haas P.J., Rijnders B.J.A., Kuijper E.J., van Tiel F.H., Varga J. (2013). Aspergillosis due to voriconazole highly resistant *Aspergillus fumigatus* and recovery of genetically related resistant isolates from domiciles. Clin. Infect. Dis..

[36] Alcazar-Fuoli L., Mellado E., Garcia-Effron G., Lopez J.F., Grimalt J.O., Cuenca-Estrella J.M., Rodriguez-Tudela J.L. (2008). Ergosterol biosynthesis pathway in *Aspergillus fumigatus*. Steroids.

[37] Monk B.C., Keniya M.V. (2021). Roles for Structural Biology in the Discovery of Drugs and Agrochemicals Targeting Sterol 14α-Demethylases. J. Fungi.

[38] Riley J., Brand S., Voice M., Caballero I., Calvo D., Read K.D. (2015). Development of a Fluorescence-based *Trypanosoma cruzi* CYP51 Inhibition Assay for Effective Compound Triaging in Drug Discovery Programmes for Chagas Disease. PLoS Neglected Trop. Dis..

[39] Monk B.C., Keniya M.V., Sabherwal M., Wilson R.K., Graham D.O., Hassan H.F., Chen D., Tyndall J.D.A. (2019). Azole resistance reduces susceptibility to the tetrazole antifungal VT-1161. Antimicrob. Agents Chemother..

[40] Tyndall J.D., Sabherwal M., Sagatova A.A., Keniya M.V., Negroni J., Wilson R.K., Woods M.A., Tietjen K., Monk B.C. (2016). Structural and functional elucidation of yeast lanosterol 14α-demethylase in complex with agrochemical antifungals. PLoS ONE.

[41] Snelders E., Camps S.M., Karawajczyk A., Rijs A.J., Zoll J., Verweij P.E., Melchers W.J. (2015). Genotype–phenotype complexity of the TR 46/Y121F/T289A *cyp51A* azole resistance mechanism in *Aspergillus fumigatus*. Fungal Genet. Biol..

