# Supplementary material for: The Molecular Basis of the Intrinsic and Acquired Resistance to Azole Antifungals in Aspergillus fumigatus
Source: J Fungi (Basel). 2024 Nov 26;10(12):820. doi: 10.3390/jof10120820 (PMC11678245; doi:10.3390/jof10120820)
Supplement: Supplementary file 1 [file jof-10-00820-s001.zip › jof-3178519-supplementary.pdf]

Supplementary Materials

The molecular basis of the intrinsic and acquired resistance to azole antifungals in *Aspergillus fumigatus*.

Parham Hosseini[1], Mikhail V. Keniya[1], Alia A. Sagatova[1], Stephanie Toepfer[1], Christoph Müller[2], Joel D. A. Tyndall[3], Anette Klinger[4], Edmond Fleischer[4], and Brian C. Monk[1]*

[1] Sir John Walsh Research Institute, Faculty of Dentistry, University of Otago, Dunedin 9016, New Zealand.

[2] Department of Pharmacy, Center for Drug Research, Ludwig-Maximilian University Munich, 81377 Munich, Germany

[3] School of Pharmacy, University of Otago, Dunedin 9054, New Zealand

[4] MicroCombiChem GmbH, iNovaParc, 56283, Germany

*Correspondence: Brian C. Monk. Email: brian.monk@otago.ac.nz
Telephone: +64 3 479 7099

Keywords: *Aspergillus fumigatus* CYP51, ERG6, CPRA, eburicol, fluconazole, voriconazole, posaconazole, innate and acquired azole resistance, *Saccharomyces cerevisiae*

Table S1. Azole susceptibilities of ScCYP51 and ScCYP51 T322I expressed in *S. cerevisiae*

	MIC_{80} (μM)		
Strain	FLC	VCZ	ITC
Y2300 ADΔΔ CYP51	6.2±0.3	0.71±0.03	0.15±0.01
Y2305 ADΔΔ CYP51 T322I	6.6±0.2	0.80±0.03	0.13±0.01

Data for each strain was obtained for two technical replicates in three biological replicates.

Table S2. Yeast strains used in this study.

Strain MBL Yeast Collection #	Strain name	Genotype	Source
Y1857	ADΔΔ	ADΔ, *Δhis1::dpl200*	Sagatova et al, 2015 [28]
Y2411	Y2411	ADΔΔ,*Δpdr5::pABC3 URA3*	Sagatova *et al* 2016 [15]
Y2300	Y2300	ADΔΔ *Δpdr5*::*ScCyp51-6xHis PGKter URA3, ΔScCyp51*::*His1*	Sagatova *et al* 2016 [15]
Y2305	Y2305	ADΔΔ *Δpdr5*::*ScCyp51-6xHis T322I PGKter URA3, ΔScCyp51*::*His1*	Sagatova *et al 2016* [15]
Y2754	ADLS	ADΔΔ *Δpdr5*::*ScCyp51-6xHis PGKter URA3, ΔScCyp51*::*His1*	This study
	A	ADΔΔ *Δpdr5*::*AfCyp51A-6xHis PGKter URA3*	This study
	B	ADΔΔ *Δpdr5*::*AfCyp51B-6xHis PGKter URA3*	This study
	AR	ADΔΔ *Δpdr5*::*AfCyp51A-6xHis PGKter URA3, Δpdr15*::*AfCprA2-6xHis PGKter LoxPHis*	This study
	BR	ADΔΔ *Δpdr5*::*AfCyp51B-6xHis PGKter URA3, Δpdr15*::*AfCprA2-6xHis PGKter LoxPHis*	This study
Y2746	ARE	ADΔΔ *Δpdr5*::*AfCyp51A-6xHis PGKter URA3, Δpdr15*::*AfCprA2 PGKter, ΔScCyp51*::*AfErg6-FLAG SYNter LoxPHis*	This study
Y2750	Y121F	ADΔΔ *Δpdr5*::*AfCyp51A-6xHis Y121F PGKter His1, Δpdr15*::*AfCprA2 PGKter LoxP, ΔScCyp51*::*AfErg6-FLAG SYNter LoxPHis*	This study
Y2751	T289A	ADΔΔ *Δpdr5*::*AfCyp51A-6xHis T289A PGKter His1, Δpdr15*::*AfCprA2 PGKter LoxP, ΔScCyp51*::*AfErg6-FLAG SYNter LoxPHis*	This study
Y2752	I301T	ADΔΔ *Δpdr5*::*AfCyp51A-6xHis I301T PGKter His1, Δpdr15*::*AfCprA2 PGKter LoxP, ΔScCyp51*::*AfErg6-FLAG SYNter LoxPHis*	This study
Y2753	Y121F T289A	ADΔΔ *Δpdr5*::*AfCyp51A-6xHis Y121F T289A PGKter URA3, Δpdr15*::*AfCprA2 PGKter LoxP, ΔScCyp51*::*AfErg6-FLAG SYNter LoxPHis*	This study
Y2747	BRE	ADΔΔ *Δpdr*::*AfCyp51B-6xHis PGKter URA3 ,Δpdr15*::*AfCprA2 PGKter LoxP, ΔScCyp51*::*AfErg6-FLAG SYNter LoxPHis*	This study
Y663	ADΔ	*MAT alpha, PDR1-3, Δura3::dpl200, his1, Δyor1::hisG, Δsnq2::hisG, Δpdr10::hisG, Δpdr11::hisG, Δycf1::hisG, Δpdr3::hisG, Δpdr15::hisG*	Lamping et al 2007 [22]

MBL: Molecular Biosciences Laboratory, Sir John Walsh Research Institute, University of Otago

Journal of Fungi

MDPI

Table S3. DNA oligonucleotide primers used in this study.

Purpose	Name	Sequence (5’→ 3’)
PDR5	PDR5up	GCATAAAACAGAGAGGCGATATAGG
	PDR5Fv2	GCAGTCCCTTACATAGTACACAAC
	PDR5Fv3	TCGCATTCTGCGCCTTCGAGCAC
	PDR5_186DS_R	TTCGGACATTGAACTTTGATTTATC
	PDR5_288DS_R	CCGTAAGGCACAGTTAAGAAATAATG
	PDR5down	TATGAGAAGACGGTTCGCCATTCGGACAG
PDR15	PDR15_957USF	CAGACGCCAACAAACCCGGCACAGC
	PDR15_924USF	GAGTCGTGGGGCCTTACGAACGG
	PDR15USF_m	GTCACGCCGCCGAACTGCAGCGCGC
	PDR15DSR_m	GATGGAATAATCCAGTTCGACTCTG
ERG11	ScErg11_Up801	GCCGCCTGTCCCGTACAGACGAAC
	ScErg11_Up773	GCAACAATGGGCGGTTGTTTAGAG
	ScErg11DS346R	GACTGCTTTATTTCCGCTTGGCCTG
	ScErg11-down	ATGGTTCAATTCACTCATCATTTGG
pJ201 plasmid	pABC3_PacI_F	CCGCTCGTTCGAAAGACTTAATTAAAAAATG
	pABC3_PacI_R	CATTTTTTAATTAAGTCTTTCGAACGAGCGG
	Not1-6xHis_F	GGCGGCCGCCATCATCACCATCATCATTAAATTC
	Not1-6xHis_R	GAATTTAATGATGATGGTGATGATGGCGGCCGCC
PGK ter.	PGK1_reverse	TCGGATAAGAAAGCAACACCTGG
	PGK352_rev	TCCAACGAACGCAGAATTTTCGAG
6xHis Deletion	*AfCprA2-6xHis_F	ATCTTACCAAGAG-GATGTATGGTCCTAAATTCGGCCGCTTCTTTGGAATT
	*AfCprA2-6xHis_R	AATTCCAAAGAAGCGGCCGAATTTAGGAC-CATACATCCTCTTGGTAAGAT
Open reading frame	*AfCyp51A-599F	GCTAGAGCTTTACAAGGTCAAGAGG
	*AfCyp51B-597F	CACCGCTTCTAGATCTCTACAAGGT
	*AfCprA1_F	GGGGAACCTGATATTCGTAGAGAC
	*AfCprA2_F	GTGTACCTCGGTGAACCTACTCAAG
	*AfC24-535_F	GGGTGTGTACAAAGAGATTTTCAGGG
	ScErg11ORF157_F	AAGGACCGTCCACCTCTAGTGTTTTAC
	ScErg11ORF804_R	CAAAGACATGTAAGTACCGGAGATAG
	ScHis1_ORF0_F	ATGGATTTGGTGAACCATCTAACCG
	ScHis1ORF-27_R	ATCGGTTAGATGGTTCACCAAATCC
Fusion primers	LoxP_PDR15DSr	CAGTAGAGAGAATAGAATATAATAAAAAGATAA-TATAACTAAAAAAAAGGAAAATAAC-GTCACCGCATAGGCCACTAGTGGATCTG
	*F.Erg11-PDR5R	GTGCTCGAAGGCGCAGAATGCGATCGTGGAT-TATCGGCCTTTAAGATTG

Purpose Name	Sequence (5’→ 3’)
*F.tagC24_SynTer_R	CTTTATTTCTAGACAG-TTATATATATATATATATATATATATATACCACCCATTACTT-GTCGTCATCGTCTTTGTAGTCGCGGCCGCCCTCAGGCTTTCTAC
*F.SynTer_LoxP_F	CTGTCTAGAAATAAAGAGTATCATCTTTCAAAGAA-GCTTCGTACGCTGCAGGTCGAC

* Primers designed for this study.

Table S4. Mutagenic primers used to prepare modified AfCYP51 isoforms.

Name	Sequence (5’ → 3’)	Substitution
**AMu.Y121F_Cyp51A_F**	CGGCTCCGATGTTGTT**TTC**GATTGCCCTAACTC	TAC → TTC
**AMu.Y121F_Cyp51A_R**	GAGTTAGGGCAATCGAAAACAACATCGGAGCCG	
**AMu.T289A_Cyp51A_F**	GCACATATGATGATC**GCT**CTTTTGATGGC	ACA →GCT
**AMu.T289A_Cyp51A_R**	GCCATCAAAAGAGCGATCATCATATGTGC	
**AMu.I301T_Cyp51A_F**	CATTCTTCTTCCTCT**ACA**TCAGCATGG	ATA → ACA
**AMu.I301T_Cyp51A_R**	CCATGCTGATGTAGAGGAAGAAGAATG	
**BMu.A303T_Cyp51B_F**	GCCCATATGATGATC**ACA**CTTTTGATGGCGGG	GCA → ACA
**BMu.A303T_Cyp51B_R**	CCCGCCATCAAAAGTGTGATCATCATATGGGC	
**BMu.T315I_Cyp51B_F**	CACTCATCTAGCAGT**ATA**GCATCTTGG	ACT→ATA
**BMu.T315I_Cyp51B_R**	CCAAGATGCTATACTGCTAGATGAGTG	

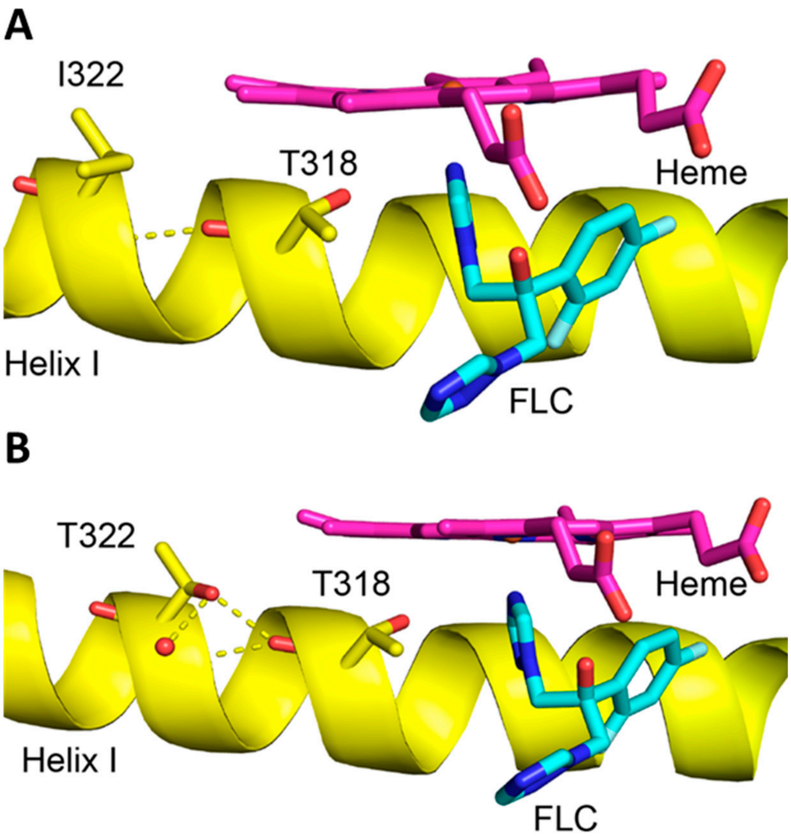

Figure S1. Impact of ScCYP51 T322 and the T322I mutation on helix I. Residue 322 interactions in (A) ScErg11-6×His T322I and (B) wild type ScErg11-6×His crystal structures in complex with FLC (PDB: 5ESM and 4WMZ, respectively). Carbon atoms are shown in yellow for I322, T322 and T318 and in cyan for FLC. Helix I is presented as yellow cartoon and hydrogen bonds as yellow dashed lines.

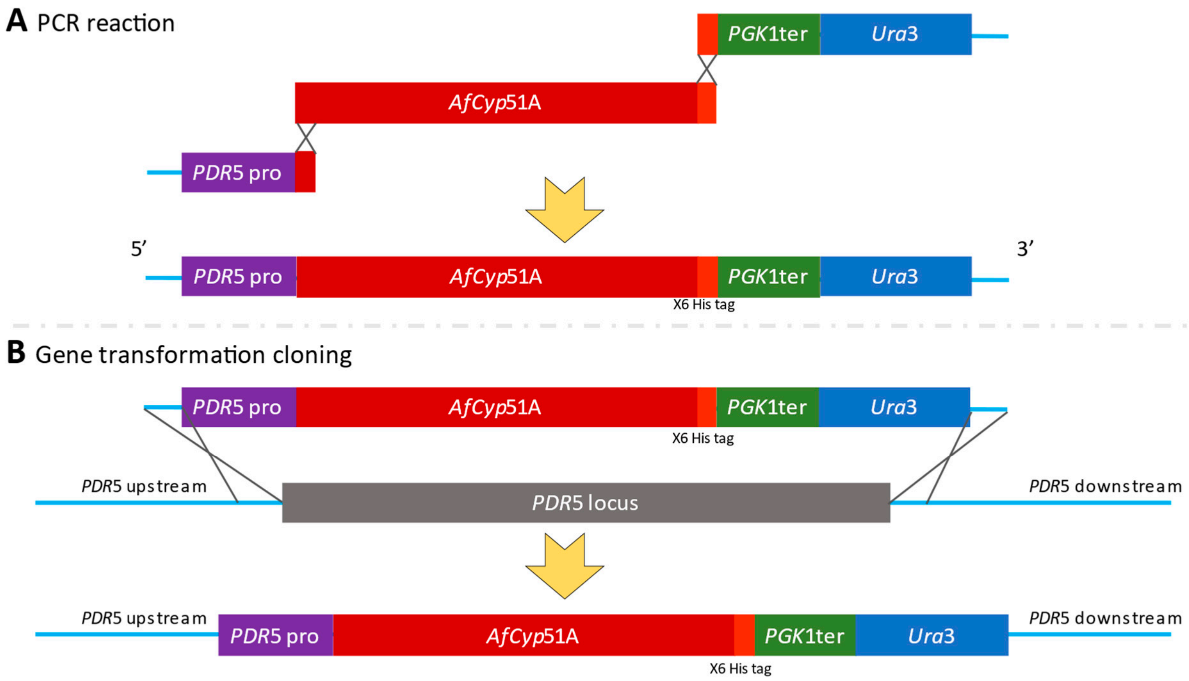

Figure S2. Strategies for preparation of recombinant yeast strains**.** Homologous recombination was used to insert a codon optimized and PCR-generated gene of interest at the *PDR5* locus of the yeast genome. A single PCR fragment recombination transformation strategy was used to create ADΔΔ-*AfCyp51-6×His* strains. (A) Specific primers were used to amplify PCR fragments comprising the gene of interest (such as *AfCyp*51A, from pJ201-*AfCyp*51A-6xHis), and the *PDR5* promotor, the *PGK1* terminator plus the *Ura3* selective marker from MBL strain Y2294. PCR fragments were fused using recombinant PCR. The *PDR5* promoter and upstream/downstream sequences, the *PGK1* terminator and the *URA3* selection marker are in purple, light blue, green and blue, respectively. (B) The transformation cassette was cloned by homologous recombination at the *PDR5* locus (grey colour) of strain ADΔΔ.

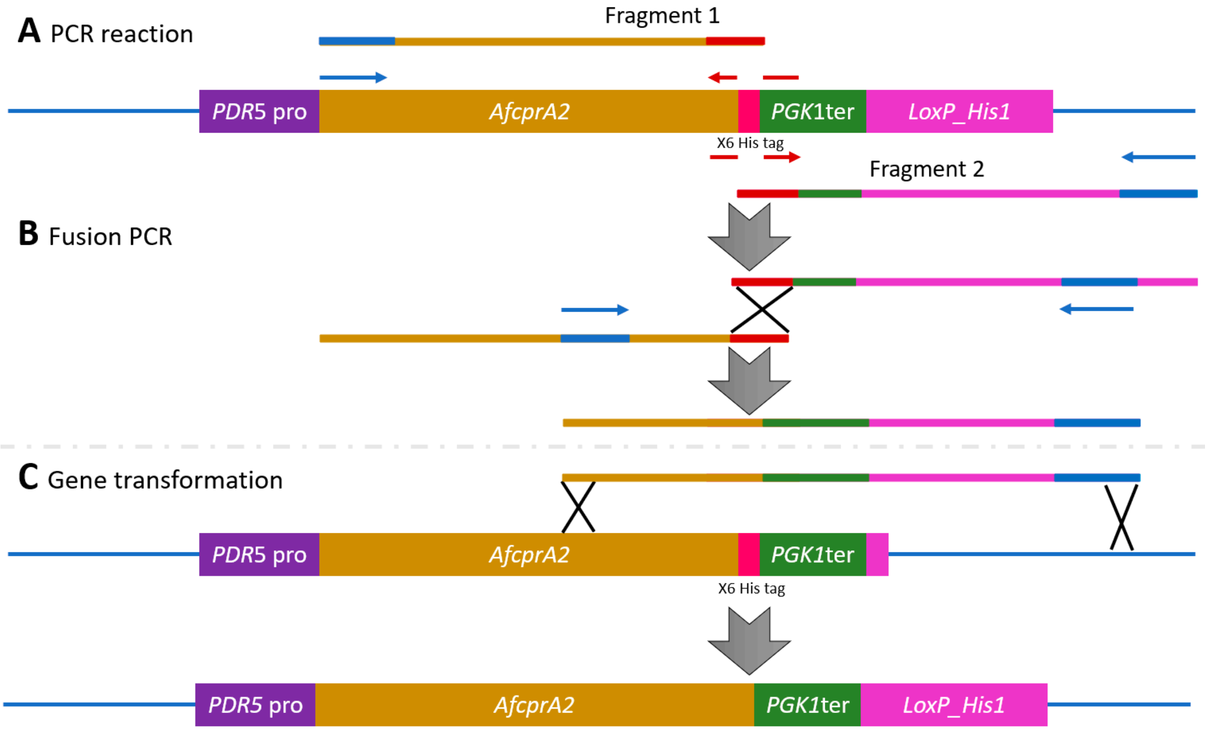

Figure S3. Hexahistidine tag removal from *AfCprA2-6×His* in the *S. cerevisiae PDR15* locus. (A) Deletion of the hexahistidine tag from the *AfCprA2-6×His* gene was achieved using recombinant PCR. Two PCR fragments, one including sequences upstream and the other including sequences downstream of the target gene stop codon were amplified using overlapping primers designed to delete the hexahistidine tag (red interrupted arrows). (B) PCR fragments 1 and 2 were joined using a second PCR reaction. (C) The new fragment was transformed into the DNA of competent cells lacking *His1* selective marker by homologous recombination.

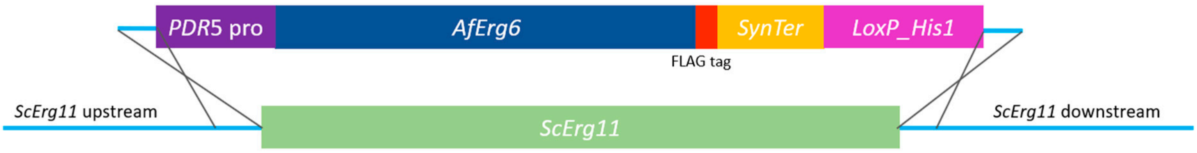

Figure S4. Scheme for replacement of the native *S. cerevisiae Erg11* (*ScErg11*) with codon optimised *AfErg6* with a C-terminal *FLAG* tag.

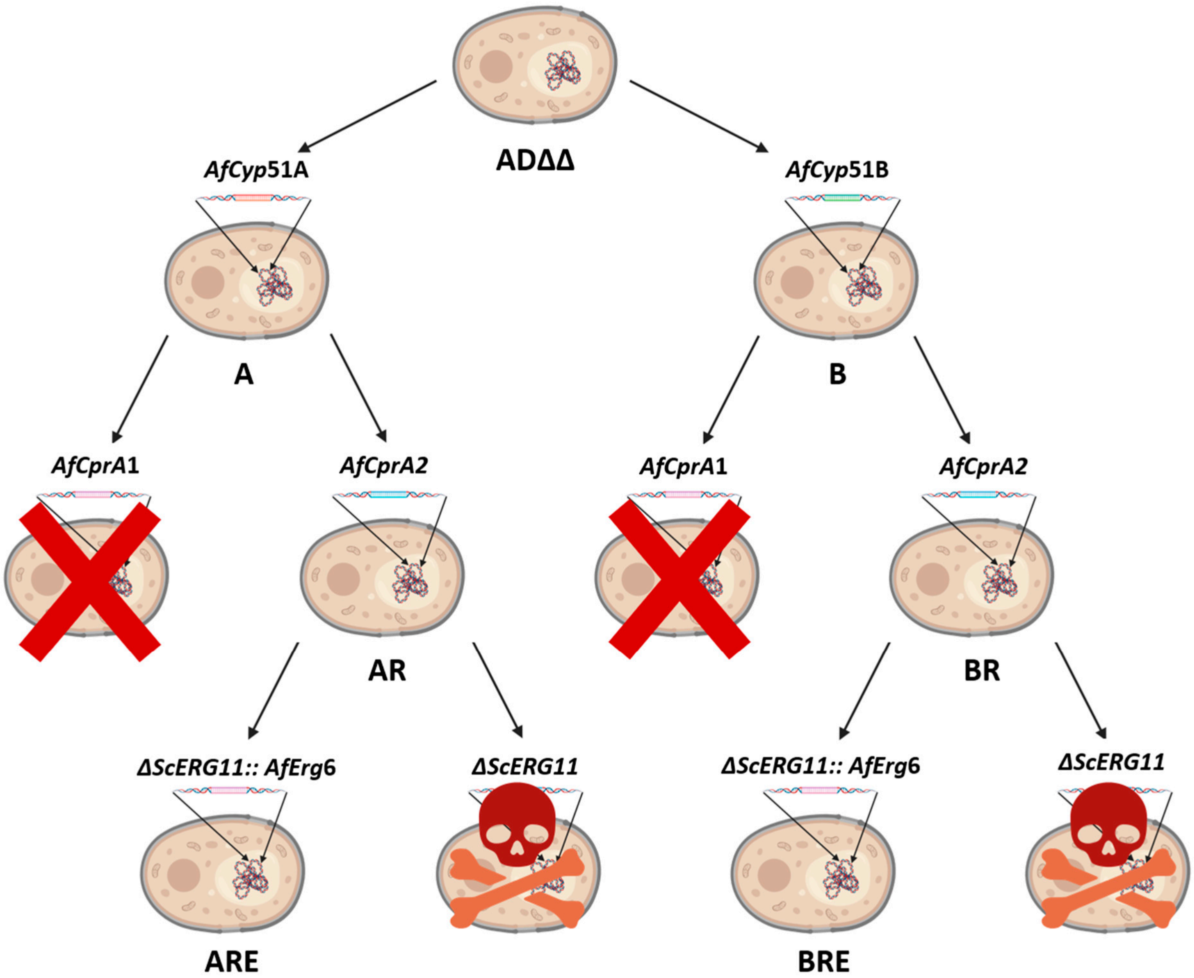

Figure S5. Strategy used to create recombinant *S. cerevisiae* constructs expressing AfCYP51A or AfCYP51B, AfCPR and AfERG6. The recombinant genes *AfCyp51A* and *AfCyp51B* were inserted at the *S. cerevisiae PDR5* locus, *AfCprA2* at the *PDR15* locus and *AfErg6* at the *ERG11* locus. The expression of each recombinant construct was regulated by the pdr1-3 transcriptional regulator acting on the *PDR5* promoter upstream of the introduced gene. *AfCprA2* but not *AfCprA1* (indicated by **X**) could be successfully integrated at the *PDR15* locus in strains A and B. *ScERG11* was deleted in the AR and BR strains when it was replaced with *AfErg6*. Deleting *ScERG11* without introducing *AfErg6* resulted in the death of isolates, as indicated by ☠. The image was created with BioRender.com.

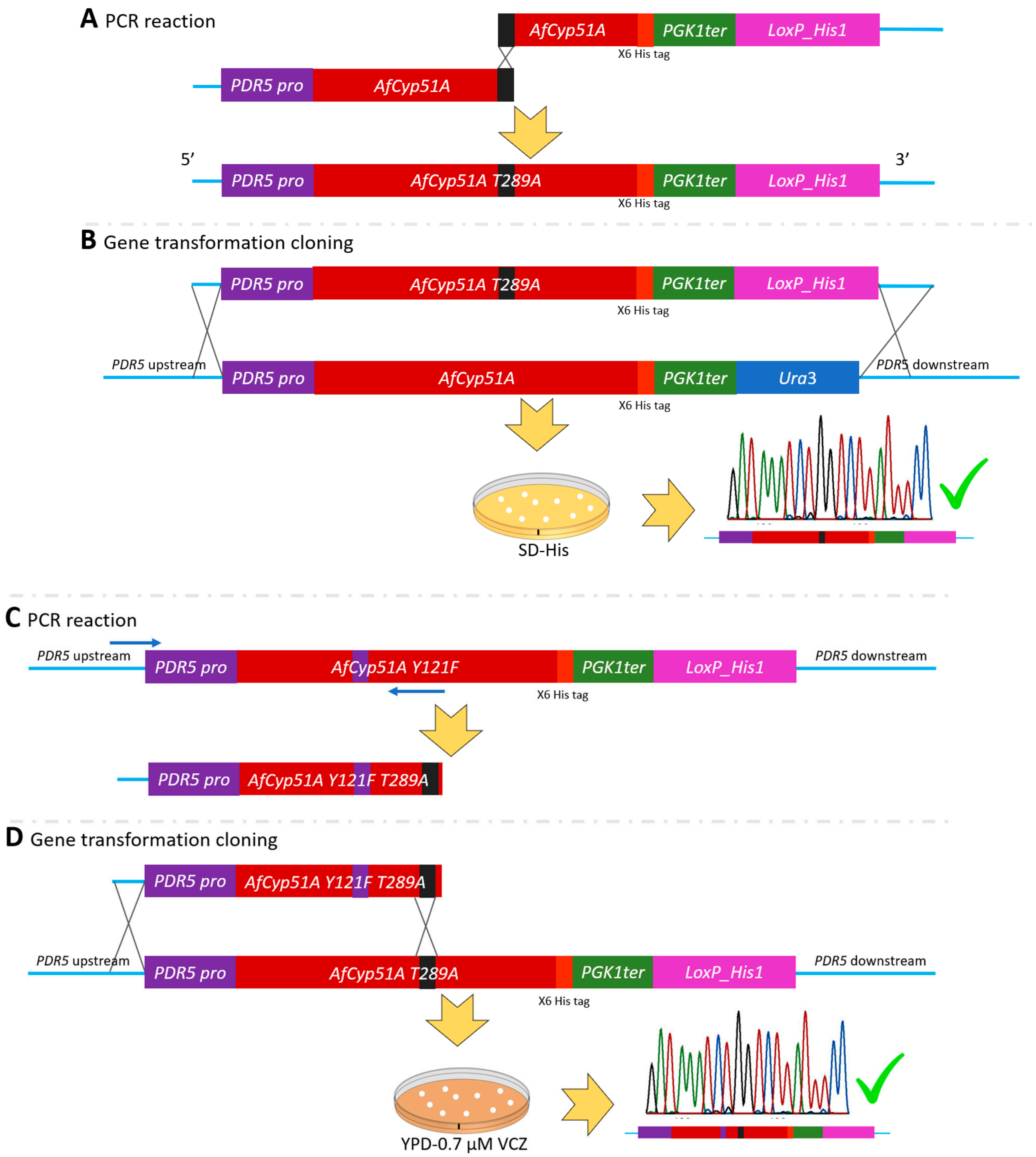

Figure S6. Recombinant PCR-based introduction of single or double amino acid mutations into AfCYP51A. (A, B) Creation of AfCYP51A-6×His T289A. (A) Two overlapping PCR fragments, one including sequences upstream of the T289A mutation in *AfCyp51A,* amplified using primers PDR5Fv2/AMu.T289A_Cyp51A_R, and the other including sequences downstream of T289A mutation amplified using primers AMu.T289A_Cyp51A_F/PDR5_288DS_R, enabled the placement of the target mutation ACA → GCT needed to generate the open reading frame encoding AfCYP51A T289. The overlapping PCR fragments were fused using primers PDR5Fv3/ PDR5_186DS_R in a

second PCR reaction. (B) The transformation cassette was cloned by homologous recombination at the *PDR5* locus of the ARE-h strain. *LoxP_His1* downstream within the cassette allowed the selection of His+ transformants using SD-His dropout medium. DNA sequence analysis was used to confirm the sequence of the mutated gene inserted in the *S. cerevisiae* genome at the *PDR5* locus. (C,D) Creation of AfCYP51A-6×His Y121F T289A. (C) A PCR fragment containing the target TAC → TTC mutation that encodes Y121F in AfCYP51A were amplified from gDNA of a Y121F strain using primers PDR5Fv3/AMu.T289A_Cyp51A_R. (D) The DNA fragment encoding the Y121F mutation was cloned by homologous recombination at the *PDR5* locus of the T289A strain created in B. Selective YPD medium containing 0.7 μM VCZ was used to identify the Y121F T289A transformants. DNA sequence analysis confirmed both mutations in the *AfCyp51A-6×His* gene inserted at the *PDR5* locus of the Y121F T289A strain.

Figure S7. Validation of recombinant *S. cerevisiae* strains

A. DNA sequence analysis of recombinant *AfCyp51* genes

Sequence of *AfCyp51A-6×His* cassette in *Saccharomyces cerevisiae PDR5* locus:*PDR5::AfCyp51A-6×His-URA3* (PDR5Fv2/ PDR5_186DS_R)

The forward PDR5Fv2 priming site at the 5’ end of the DNA sequence and the reverse PDR5_186DS_R priming site at the 3’ end of the DNA sequence is highlighted in grey. The codon optimised *AfCyp51A-6×His* open reading frame (ORF) is highlighted in yellow. The *Ura3* selective marker is in bold.

GCAGTCCCTTACATAGTACACAACATTTATCACTTCACACAATCAGGAGTGGAACTCAATGGAAAACAACACCACACGTATGATCTT

ACTAATAAAAGAACATGAACGTTCCTCAGCGCGAACGTTCGCATTCTGCGCCTTCGAGCACAGGATAAGTTGCAGGAAGCCATCACA

TCTATGCAACGATTATCACGACACAACCTTGCCGCCGAGAAAACGTCCGTGGAGAACCATTCGGTCGATTGCTTCCCACGGAACGAG

TGGACTGAAACTTAAGACTGCCCCTCTCTTTCCGCGGAATCGCTCATGCCGCGGTGCCACAACATTTTCAGATTTACTAAGACTCCG

GTGAGTGTGGGCTCACCCGCGGGTCGTGATCACGATTCAGCACCCTTTGGACTCGTGATTCCGTGGAAAGGTCAGATCTGTATTCCT

ACTTATGGTAATGTGCTAAAAAAAGAGAAATGTCTCCGCGGAACTCTTCTACGCCGTGGTACGATATCTGTTGAACGTAATCTGAGC

AATACAAACAAGGCCTCTCCTATACATATATAATTGTGATGTGCATAACCTTATGGCTGTTCGCTTTTATTACCATACCTTAGAATG

AAATCCAAAAGAAAAAAGTCACGCAAAGTTGCAAACATATAACAACTGTGTTAGTTATCACTCGACTTTGTTATTCTAATTATAAAT

AAATTGGCAACTAGGAACTTTCGAAAAAGAAATTAAAGACCCTTTTAAGTTTTCGTATCCGCTCGTTCGAAAGACTTAATTAAAAAA

TGGTCCCTATGCTCTGGTTGACCGCATACATGGCAGTTGCAGTTCTCACAGCCATCTTACTAAACGTAGTTTATCAGTTGTTTTTCC

GTTTGTGGAACAGAACAGAACCTCCAATGGTGTTCCATTGGGTGCCATACCTGGGCTCAACCATTTCATACGGTATTGATCCATACA

AGTTCTTCTTCGCTTGTAGAGAGAAATACGGTGACATTTTCACATTCATTCTACTGGGACAAAAGACAACAGTTTACCTGGGTGTTC

AAGGTAATGAATTCATACTGAATGGCAAGTTAAAAGACGTCAATGCCGAAGAGGTTTATTCTCCATTGACAACACCAGTTTTCGGCT

CCGATGTTGTTTACGATTGCCCTAACTCTAAGCTTATGGAACAGAAAAAGTTTATCAAGTACGGTCTTACCCAATCAGCCTTGGAAT

CACATGTCCCTTTAATCGAGAAGGAAGTGCTTGACTACCTAAGAGATAGCCCTAACTTTCAAGGTTCTAGTGGTAGAGTCGATATTT

CTGCAGCAATGGCAGAAATCACAATCTTTACTGCGGCTAGAGCTTTACAAGGTCAAGAGGTAAGATCTAAATTGACTGCAGAATTTG

CTGACTTGTACCACGATTTGGATAAAGGTTTCACTCCAATCAACTTTATGCTACCATGGGCCCCATTACCACACAACAAAAAGAGAG

ATGCTGCTCATGCTAGAATGAGATCAATCTATGTTGACATTATCACCCAGAGAAGATTAGACGGGGAAAAAGACAGCCAAAAGTCTG

ATATGATTTGGAATTTGATGAATTGTACATACAAAAATGGTCAACAGGTACCTGATAAGGAGATTGCACATATGATGATCACACTTT

TGATGGCCGGACAACATTCTTCTTCCTCTATATCAGCATGGATTATGTTGAGATTAGCTTCTCAACCAAAGGTATTAGAAGAGTTGT

ACCAAGAGCAACTCGCGAACTTAGGTCCTGCGGGTCCAGATGGATCATTACCACCATTGCAATACAAGGACCTTGATAAGCTTCCTT

TTCATCAACATGTCATTAGAGAAACTTTGAGAATTCATAGTAGTATCCACTCTATAATGAGAAAGGTCAAATCCCCATTACCTGTCC

CAGGTACTCCATACATGATACCACCAGGCAGAGTTCTTCTAGCATCACCTGGTGTAACTGCCCTGTCAGATGAGCATTTCCCAAACG

CTGGCTGTTGGGATCCACATAGATGGGAAAACCAGGCCACCAAGGAACAGGAAAATGATAAGGTGGTTGATTACGGATATGGAGCTG

TGTCAAAGGGTACTTCCAGCCCATACCTCCCATTTGGGGCAGGACGTCACAGATGCATCGGAGAAAAGTTCGCTTACGTCAACTTAG

GTGTTATCTTGGCTACAATAGTGAGACATTTGCGTCTATTCAACGTTGATGGAAAAAGGGTGTTCCAGAAACAGATTACTCTTCTT

TGTTCAGTGGTCCTATGAAGCCTTCAATTATCGGCTGGAAAAGAGATCTAAAAACACTTCCAAAGGCGGCCGCCATCATCACCATC

ATCATTAAATTCGGCCGCTTCTTTGGAATTATTGGAAGGTAAGGAATTGCCAGGTGTTGCTTTCTTATCCGAAAAGAAATAAATTGA

ATTGAATTGAAATCGATAGATCAATTTTTTTCTTTTCTCTTTCCCCATCCTTTACGCTAAAATAATAGTTTATTTTATTTTTTGAAT

ATTTTTTATTTATATACGTATATATAGACTATTATTTATCTTTTAATGATTATTAAGATTTTTATTAAAAAAAAATTCGCTCCTCTT

TTAATGCCTTTATGCAGTTTTTTTTTCCCATTCGATATTTCTATGTTCGGGTTCAGCGTATTTTAAGTTTAATAACTCGAAAATTCT

GCGTTCGTTGGATCCGGTGATTGATTGAGCAAGCTAGCTTTTCAATTCATCATTTTTTTTTTATTCTTTTTTTTGATTTCGGTTTCC

TTGAAATTTTTTTGATTCGGTAATCTCCGAACAGAAGGAAGAACGAAGGAAGGAGCACAGACTTAGATTGGTATATATACGCATATG

TAGTGTTGAAGAAACATGAAATTGCCCAGTATTCTTAACCCAACTGCACAGAACAAAAACCGGAAACGAAGATAAATC**ATGTCGAAAGCTACATATAAGGAACGTGCTGCTACTCATCCTAGTCCTGTTGCTGCCAAGCTATTTAATATCATGCACGAAAAGCAAACAAACTTGTGTGCTTCATTGGATGTTCGTACCACCAAGGAATTACTGGAGTTAGTTGAAGCATTAGGTCCCAAAATTTGTTTACTAAAAACACATGTGGATATCTTGACTGATTTTTCCATGGAGGGCACAGTTAAGCCGCTAAAGGCATTATCCGCCAAGTACAATTTTTTACTCTTCGAAGACAGAAAATTTGCTGACATTGGTAATACAGTCAAATTGCAGTACTCTGCGGGTGTATACAGAATAGCAGAATGGGCAGACATTACGAATGCACACGGTGTGGTGGGCCCAGGTATTGTTAGCGGTTTGAAGCAGGCGGCAGAAGAAGTAACAAAGGAACCTAGAGGCCTTTTGATGTTAGCAGAATTGTCATGCAAGGGCTCCCTATCCACTGGAGAATATACTAAGGGTACTGTTGACATTGCGAAGAGCGACAAAGATTTTGTTATCGGCTTTATTGCTCAAAGAGACATGGGTGGAAGAGATGAAGGTTACGATTGGTTGATTATGACACCCGGTGTGGGTTTAGATGACAAGGGAGACGCATTGGGTCAACAGTATAGAACCGTGGATGATGTGGTCTCTACAGGATCTGACATTATTATTGTTGGAAGAGGACTATTTGCAAAGGGAAGGGATGCTAAGGTAGAGGGTGAACGTTACAGAAAAGCAGGCTGGGAAGCATATTTGAGAAGATGCGGCCAGCAAAACTAA**TAGAATTTTGAATTTGGTTAAGAAAAGAAACTTACCAAGATGGACTTTTTTAATAAACATACATAATCACTACATATAGGTGCGTAATAATAAGTTTTTATTTTTTTTTTCTTAATTCAGCGAGCTTTCTACATTTCAATTTTCTTGAATTTACCAGCTCTGATAAATCAAAGTTCAATGTCCGAA

Sequence of *AfCyp51B-6×His* cassette in *S. cerevisiae PDR5* locus:
***PDR5::AfCyp51B-6×His-URA3* (PDR5Fv2/ PDR5_186DS_R)**

The forward PDR5Fv2 priming site at the 5’ end of the DNA sequence and the reverse PDR5_186DS_R priming site at the 3’ end of the DNA sequence is highlighted in grey. The codon optimised *AfCyp51B-6×His* ORF is highlighted in yellow. The *Ura3* selective marker is in bold.

GCAGTCCCTTACATAGTACACAACATTTATCACTTCACACAATCAGGAGTGGAACTCAATGGAAAACAACACCACACGTATGATCTT
ACTAATAAAAGAACATGAACGTTCCTCAGCGCGAACGTTCGCATTCTGCGCCTTCGAGCACAGGATAAGTTGCAGGAAGCCATCACA
TCTATGCAACGATTATCACGACACAACCTTGCCGCCGAGAAAACGTCCGTGGAGAACCATTCGGTCGATTGCTTCCCACGGAACGAG
TGGACTGAAACTTAAGACTGCCCCTCTCTTTCCGCGGAATCGCTCATGCCGCGGTGCCACAACATTTTCAGATTTACTAAGACTCCG
GTGAGTGTGGGCTCACCCGCGGGTCGTGATCACGATTCAGCACCCTTTGGACTCGTGATTCCGTGGAAAGGTCAGATCTGTATTCCT
ACTTATGGTAATGTGCTAAAAAAAGAGAAATGTCTCCGCGGAACTCTTCTACGCCGTGGTACGATATCTGTTGAACGTAATCTGAGC
AATACAAACAAGGCCTCTCCTATACATATATAATTGTGATGTGCATAACCTTATGGCTGTTCGCTTTTATTACCATACCTTAGAATG
AAATCCAAAAGAAAAAAGTCACGCAAAGTTGCAAACATATAACAACTGTGTTAGTTATCACTCGACTTTGTTATTCTAATTATAAAT
AAATTGGCAACTAGGAACTTTCGAAAAAGAAATTAAAGACCCTTTTAAGTTTTCGTATCCGCTCGTTCGAAAGACTTAATTAAAAAA
TGGGTCTGATAGCATTTATCTTAGATGGGATATGTAAGCACTGTTCAACACAAAGCACATGGGTTCTAGTTGGTATTGGCTTACTAT
CCATATTGGCCGTTTCAGTCATCATCAACGTATTGCAGCAATTGTTATTCAAAAACCCACACGAGCCTCCAGTCGTGTTTCATTGGT
TTCCATTCATCGGATCAACAATTTCTTACGGAATTGACCCATACAAGTTCTTCTTCGACTGCAGAGCCAAGTATGGCGACATTTTCA
CTTTCATCCTCTTGGGTAAAAAGACAACTGTGTACTTGGGTACAAAAGGAAATGATTTCATCCTCAATGGGAAGCTCAGAGATGTTT
GTGCAGAAGAGGTCTATTCACCTCTAACTACTCCAGTTTTTGGTAGACATGTAGTATACGATTGTCCTAACGCTAAGTTAATGGAAC
AGAAAAAGTTTGTAAAGTACGGCCTTACCTCCGATGCTCTCAGATCTTACGTCCCTCTAATAACCGACGAAGTAGAATCATTTGTGA
AAAACTCCCCAGCATTCCAAGGACATAAAGGTGTGTTTGATGTTTGCAAAACAATAGCGGAAATCACCATTTACACCGCTTCTAGAT
CTCTACAAGGTAAGGAAGTTAGATCTAAATTCGATTCCACATTTGCTGAACTATACCATAACTTAGATATGGGATTCGCTCCAATCA
ACTTCATGTTGCCTTGGGCACCACTTCCACATAATAGAAAGAGAGATGCAGCCCAACGTAAGCTTACAGAGACTTACATGGAAATCA
TAAAGGCTCGTAGACAAGCAGGATCTAAAAAGGACTCCGAGGACATGGTTTGGAACCTAATGAGTTGCGTCTACAAAAATGGGACTC
CAGTTCCAGATGAAGAGATTGCCCATATGATGATCGCACTTTTGATGGCGGGTCAGCACTCATCTAGCAGTACAGCATCTTGGATTG
TTTTGAGATTAGCCACAAGACCAGACATTATGGAGGAACTTTACCAAGAGCAAATTCGTGTACTGGGTTCAGACTTACCTCCACTTA
CTTACGATAACTTGCAAAAGTTGGATCTGCATGCTAAGGTCATTAAGGAAACCTTGAGATTGCACGCGCCAATTCATTCTATCATCA
GAGCTGTCAAAAATCCAATGGCTGTTGATGGTACTTCATATGTTATCCCAACATCTCATAATGTTTTATCTTCACCTGGCGTGACAG
CCAGAAGTGAGGAACACTTCCCTAATCCATTAGAATGGAACCCACATAGATGGGATGAAAACATTGCAGCATCAGCCGAAGATGATG
AAAAGGTTGATTATGGTTACGGGTTGGTTAGTAAGGGTACCAACTCCCCTTACTTACCTTTCGGTGCTGGTAGACATAGATGTATTG
GTGAACAGTTTGCTTACTTGCAATTAGGTACAATCACTGCAGTGTTAGTCAGATTGTTCAGATTCAGAAACCTTCCAGGTGTTGATG
GAATTCCTGATACAGATTACTCTAGCCTGTTTTCAAAGCCTTTGGGCAGATCTTTCGTTGAATTCGAGAAAAGAGAATCTGCTACTA
AGGCAGGCGGCCGCCATCATCACCATCATCATTAAATTCGGCCGCTTCTTTGGAATTATTGGAAGGTAAGGAATTGCCAGGTGTTGC
TTTCTTATCCGAAAAGAAATAAATTGAATTGAATTGAAATCGATAGATCAATTTTTTTCTTTTCTCTTTCCCCATCCTTTACGCTAA
AATAATAGTTTATTTTATTTTTTGAATATTTTTTATTTATATACGTATATATAGACTATTATTTATCTTTTAATGATTATTAAGATT
TTTATTAAAAAAAAATTCGCTCCTCTTTTAATGCCTTTATGCAGTTTTTTTTTCCCATTCGATATTTCTATGTTCGGGTTCAGCGTA
TTTTAAGTTTAATAACTCGAAAATTCTGCGTTCGTTGGATCCGGTGATTGATTGAGCAAGCTAGCTTTTCAATTCATCATTTTTTTT
TTATTCTTTTTTTTGATTTCGGTTTCCTTGAAATTTTTTTGATTCGGTAATCTCCGAACAGAAGGAAGAACGAAGGAAGGAGCACAG
ACTTAGATTGGTATATATACGCATATGTAGTGTTGAAGAAACATGAAATTGCCCAGTATTCTTAACCCAACTGCACAGAACAAAAAC
CGGAAACGAAGATAAATC**ATGTCGAAAGCTACATATAAGGAACGTGCTGCTACTCATCCTAGTCCTGTTGCTGCCAAGCTATTTAAT**
ATCATGCACGAAAAGCAAACAAACTTGTGTGCTTCATTGGATGTTCGTACCACCAAGGAATTACTGGAGTTAGTTGAAGCATTAGGT
CCCAAAATTTGTTTACTAAAAACACATGTGGATATCTTGACTGATTTTTCCATGGAGGGCACAGTTAAGCCGCTAAAGGCATTATCC

GCCAAGTACAATTTTTTACTCTTCGAAGACAGAAAATTTGCTGACATTGGTAATACAGTCAAATTGCAGTACTCTGCGGGTGTATAC
AGAATAGCAGAATGGGCAGACATTACGAATGCACACGGTGTGGTGGGCCCAGGTATTGTTAGCGGTTTGAAGCAGGCGGCAGAAGAA
GTAACAAAGGAACCTAGAGGCCTTTTGATGTTAGCAGAATTGTCATGCAAGGGCTCCCTATCCACTGGAGAATATACTAAGGGTACT
GTTGACATTGCGAAGAGCGACAAAGATTTTGTTATCGGCTTTATTGCTCAAAGAGACATGGGTGGAAGAGATGAAGGTTACGATTGG
TTGATTATGACACCCGGTGTGGGTTTAGATGACAAGGGAGACGCATTGGGTCAACAGTATAGAACCGTGGATGATGTGGTCTCTACA
GGATCTGACATTATTATTGTTGGAAGAGGACTATTTGCAAAGGGAAGGGATGCTAAGGTAGAGGGTGAACGTTACAGAAAAGCAGGC
TGGGAAGCATATTTGAGAAGATGCGGCCAGCAAAACTAATAGAATTTTGAATTTGGTTAAGAAAAGAAACTTACCAAGATGGACTTT
TTTAATAAACATACATAATCACTACATATAGGTGCGTAATAATAAGTTTTTATTTTTTTTTTCTTAATTCAGCGAGCTTTCTACATT
TCAATTTTCTTGAATTTACCAGCTCTGATAAATCAAAGTTCAATGTCCGAA

Sequence of *AfCprA1-6×His* cassette in *S. cerevisiae PDR5* locus:

***PDR15::AfCprA1-6×His-LoxP_His1* (PDR15USF_m/PDR15DSR_m)**

The forward PDR15USF_m priming site at the 5' end of the DNA sequence and the reverse PDR15DSR_m priming site at the 3' end of the DNA sequence is highlighted in grey. The codon optimised *AfCprA1* ORF is highlighted in yellow. The *His1* selective marker is in bold between LoxP sites highlighted in red.

GTCACGCCGCCGAACTGCAGCGCGCAGATAAACTAATTGCCCTGAACAGGTACATAGAAGATCTACATTAGAAGGTTTCCCATTCCA
CCCCTTCGTTAACCCACACGCAAATACCCAGATAAGCATCTTGCACCCCTCACACTTGTATATACGGAGCAAACTACATAATCTCTA
AACAGAAAAAAAAAAAAAGAAAGAAAATTAAGAACATTACTTTTTCCGAGCAACAAACGCAAAAATATATGAAGTGGCTGTAGAACA
TGCCTTACGGAAGAAAAGAATGATGATGGCATCCCCTTCCCCTGCCCTGGAAGGTGGCCTGTATTTCTGTTCTTTTTTTATTTTTGT
TCGCGCAAAGAGCAAGAACATTGGTTGCTTTACTTATTATTTTCAGGGTCGCTTCGCATTCTGCGCCTTCGAGCACAGGATAAGTTG
CAGGAAGCCATCACATCTATGCAACGATTATCACGACACAACCTTGCCGCCGAGAAAACGTCCGTGGAGAACCATTCGGTCGATTGC
TTCCCACGGAACGAGTGGACTGAAACTTAAGACTGCCCCTCTCTTTCCGCGGAATCGCTCATGCCGCGGTGCCACAACATTTTCAGA
TTTACTAAGACTCCGGTGAGTGTGGGCTCACCCGCGGGTCGTGATCACGATTCAGCACCCTTTGGACTCGTGATTCCGTGGAAAGGT
CAGATCTGTATTCCTACTTATGGTAATGTGCTAAAAAAAGAGAAATGTCTCCGCGGAACTCTTCTACGCCGTGGTACGATATCTGTT
GAACGTAATCTGAGCAATACAAACAAGGCCTCTCCTATACATATATAATTGTGATGTGCATAACCTTATGGCTGTTCGCTTTTATTA
CCATACCTTAGAATGAAATCCAAAAGAAAAAAGTCACGCAAAGTTGCAAACATATAACAACTGTGTTAGTTATCACTCGACTTTGTT
ATTCTAATTATAAATAAATTGGCAACTAGGAACTTTCGAAAAAGAAATTAAAGACCCTTTTAAGTTTTCGTATCCGCTCGTTCGAAA
GACTTAATTAAAAAATGTCACTTTTCTCACAATGGTCCAGCCACCCAGAAATCCTTAGAGATTTATTTAAGGTCCCTTCCCCAGCTT
CCGTTGCTGATTACGCCGCTCTAACCGTTGTGTCTATCATCGCTGGCACATACGTTTCTAGAGGAATCTTGTGGGACAGACCTGACC
CATACTTACACCTCGTTTATGAACGTCCACAATTGAAAAACGGTGGTGCCGCGGGTTCTAATGCTAACAAACAGACCAGAGATGTAG
CACAAAAAATGGACGAAACTGGTAAGACAATCGTAGTCTTTTGGGGTTCACAATCTGGAACAGCTGAAGGCTTCGCACACAGGTTGG
CACGTGAAGTAAGCCTTAGATTCGGACAGGAAGCAATGACAGCCGATTTGTCAGATTACGATCCTGACTCAGTATCCAGAATACCAA
ATAGTAAGCTTGCCATTTTCATCTTATCTACTTATGGTGAGGGTGATCCATCTGATAACACCGCGGAATTCTGGGACTGGATCCATA
AGAGTCAAAATGTATCACTCTCTAACTTGCGATACGCAGCATTCGGCCTGGGAAATAGTAACTACAAGTTCTATAACAGAGTTGTTG
ACGTCGTCGTAGAAGCGCTAGATAGATTTGGTGCTAAGTCATTAATGCCTGTTGGTAAGGCTAACGATGCCGAGGGGACCACTCAAG
AGGACTTTATGTCGTGGAAAGAGAACTTGTTCGCAACCTTCAAGCAACAGTTGGGTTTCCAAGAGGCTGAAGTGAAGTACATGCCAA
CACTGTCAGTTCAAGAGGATGAATCTCTAGCGCCAATCGATCTACATCACGGGGAACCTGATATTCGTAGAGACTCTGTCAAATCAG
CCGCTCAGTGTTCTCCAGTCAGAGCCGTCGATATATCTTCTAGCAGAGAGTTGTTTGCATCTTCCGATAGACACTGTTTGCATATAG
AATTAGATTTATCCTCTCAACCAGAATTCACCTACAAGACTGGCGATCATCTGGCCGTTTGGGCAGGAAATCCTGACGCTGAAGTCG
ATATCCTTGTTAACGCACTTGGTTTGCCTCCATCTAGACATGAAATTCCAATTTCTATTACATCTTTAGATCCAGCAACAAAAGTTA
GAGTGCCAACACCTACTACACCAATCGCCCTATTCAGATACTACCTTGAAATTTGCGCTCCCGTGAACAGAGATACACTTTTGGGCT
TAGCTCAGTTTGCTCCAACTCCAGCGGCCAAAGACTTCTTGTTACAACTCGGACAGGATAAGACAGCATACGCTTCATTTCTTAATC
ATACTCATTTGACATTGGGTAGACTCTTGCAATTGGCATGTCCTGATACACCATGGACAGCATTACCATTATCCTACTTGATCGAGA
CTCTAACACCAATTCAACCAAGATACTACAGTATTTCATCTTCTAGTGTTTTGGCCCCTAGAAAACCATCTATTACAGTTTTGGTAT
CTTGTACACCATTACCAGCAGACCCAACGCAAAGCATCCACGGTCTAACTTCCAACTATCTACTAGCACTCAGTGCTTCTCTTGCTT
CATCAAGACATCCTCATGGTCTGACTTACCCTCTTGCTGGTCCTAACGCCTCTTTGACCGGTGGTAAGATCCATGCACATCTTAGAA
GATCCaGaTTCAAATTACCACTGATGTCTAAGTGTCCATTAGTGATGGTTGCTGCTGGAACTGGCTTAGCTCCTTTCAGAGCATTCA

TAGCGGAACGTAGACAACTGTCATTGATTGGCAAAGAGATTGGTGAAATGATCTTGTTTTTCGGATGCAGAGCACCTACACAAGACT
TTATTTACAAGGAAGAGTTAGAGGAACTACAGGCAGCCTTGGGTGATAAGTTGAGAGTTATTACAGCATTCTCTAGAGAGGTTGGTT
CACAAAAAGTTTACGTTCAAGATAGAATCGCAGAACACGCAGCTGAAGTCATAAGACTCATAGATGAAGGTGCATCAGTTTACATTT
GCGGTAGAGCCGGGATGGCTAGAGAAGTGGAAAAAACTGTGGGGGAAGCGATGAGAGCTGCAAAGCGTTGGTCAGAAACAGAGTTAA
ACGAATGGTCCAGAGCCATTAAGAGAAAAAATAAGTGGCAGGAAGATGTCTGGGGcGGCGGCCGCCATCATCACCATCATCATTAAA
TTCGGCCGCTTCTTTGGAATTATTGGAAGGTAAGGAATTGCCAGGTGTTGCTTTCTTATCCGAAAAGAAATAAATTGAATTGAATTG
AAATCGATAGATCAATTTTTTTCTTTTCTCTTTCCCCATCCTTTACGCTAAAATAATAGTTTATTTTATTTTTTGAATATTTTTTAT
TTATATACGTATATATAGACTATTATTTATCTTTTAATGATTATTAAGATTTTTATTAAAAAAAAATTCGCTCCTCTTTTAATGCCT
TTATGCAGTTTTTTTTTCCCATTCGATATTTCTATGTTCGGGTTCAGCGTATTTTAAGTTTAATAACTCGAAAATTCTGCGTTCGTT
GGAGAAGCTTCGTACGCTGCAGGTCGACAACCCTTAATATAACTTCGTATAatgtatgcTATACGAAGTTATTAGGTCTAGAGATCT
GTTTAGCTTGCCTCGTCCCCGCCGGGTCACCCGGCCAGCGACATGGAGGCCCAGAATACCCTCCTTGACAGTCTTGACGTGCGCAGC
TCAGGGGCATGATGTGACTGTCGCCCGTACATTTAGCCCATACATCCCCATGTATAATCATTTGCATCCATACATTTTGATGGCCGC
ACGGCGCGAAGCAAAAATTACGGCTCCTCGCTGCAGACCTGCGAGCAGGGAAACGCTCCCCTCACAGACGCGTTGAATTGTCCCCAC
GCCGCGCCCCTGTAGAGAAATATAAAAGGTTAGGATTTGCCACTGAGGTTCTTCTTTCATATACTTCCTTTTAAAATCTTGCTAGGA
TACAGTTCTCACATCACATCCGAACATAAACAACC**ATGGATTTGGTGAACCATCTAACCGATAGACTACTGTTTGCAATCCCAAAGA**
AAGGTCGTTTATATTCTAAAAGTGTTTCTATTTTGAATGGTGCTGATATTACCTTTCACCGCTCTCAAAGATTAGACATTGCACTAA
GCACAAGCTTACCTGTAGCGTTGGTCTTTCTGCCCGCTGCAGATATTCCAACTTTTGTTGGTGAAGGTAAATGTGATCTTGGTATAA
CTGGTGTTGACCAAGTTCGTGAATCTAACGTCGACGTAGACTTAGCAATCGATTTGCAATTTGGTAACTGTAAATTGCAGGTACAAG
TCCCCGTAAATGGCGAGTATAAAAAGCCAGAACAGTTAATTGGCAAAACCATTGTTACCAGTTTCGTGAAACTTGCTGAAAAATACT
TTGCCGATTTGGAAGGTACTACTGTTGAAAAAATGACCACAAGGATAAAGTTTGTCAGTGGTTCCGTGGAGGCATCATGTGCTCTGG
GAATTGGTGATGCTATTGTAGATCTTGTAGAGAGTGGTGAGACAATGAGGGCAGCAGGTTTAGTTGATATTGCCACCGTCCTAAGCA
CAAGTGCCTACCTAATAGAATCAAAGAACCCAAAGAGCGATAAGAGTTTGATTGCTACTATCAAATCAAGAATTGAAGGTGTCATGA
CCGCTCAAAGGTTCGTTTCATGTATTTATAACGCACCTGAAGACAAGCTGCCTGAACTGTTGAAGGTGACGCCTGGCCGTAGAGCAC
CAACCATTTCCAAAATTGACGATGAAGGATGGGTTGCTGTTAGTTCCATGATTGAGAGAAAAACGAAGGGTGTTGTTTTAGATGAAT
TGAAAAGACTCGGCGCATCTGATATCATGGTTTTCGAAATTTCTAATTGTCGTGTATAATCAGTACTGACAATAAAAAGATTCTTGT
TTTCAAGAACTTGTCATTTGTATAGTTTTTTTATATTGTAGTTGTTCTATTTTAATCAAATGTTAGCGTGATTTATATTTTTTTTCG
CCTCGACATCATCTGCCCAGATGCGAAGTTAAGTGCGCAGAAAGTAATATCATGCGTCAATCGTATGTGAATGCTGGTCGCTATACT
GCTGTCGATTCGATACTAACGCCGCCATCCAGTGTCGAAAACGAGCTCTCGAGAACCCTTAATATAACTTCGTATAatgtatgcTAT
ACGAAGTTATTAGGTGATATCAGATCCACTAGTGGCCTATGCGGTGACGTTATTTTCCTTTTTTTTAGTTATATTATCTTTTTATTA
TATTCTATTCTCTCTACTGGGGAGAAATTACTTAATTTTCATTTATATTAAAATAATACCTACTGTTTCATACTTTCACTTTAACCA
CCATTTCTTTAAGCTAAAGTTTAGGAACGCTAAACATATCATTGTTTACTTTTCGAGGAAACATAACTTTCAACATCAGGCTCTTTT
CGTTTTATATTATCTATATTCTATATAGTAGTTTATAGAAATGCATTTAACACTAAATAACAGACAAGATAACGTTTTCAGAGTCGA
ACTGGATTATTCCATC

Sequence of *AfCprA2-6×His* cassette in *S. cerevisiae PDR15* locus:
***PDR15::AfCprA2-6×His-LoxP_His1* (PDR15USF_m/PDR15DSR_m)**

The forward PDR15USF_m priming site at the 5' end of the DNA sequence and the reverse PDR15DSR_m priming site at the 3' end of the DNA sequence is highlighted in grey. The codon optimised *AfCprA2-6×His* ORF is highlighted in yellow. The *His1* selective marker is in bold between LoxP sites highlighted in red.

GTCACGCCGCCGAACTGCAGCGCGCAGATAAACTAATTGCCCTGAACAGGTACATAGAAGATCTACATTAGAAGGTTTCCCATTCCA
CCCCTTCGTTAACCCACACGCAAATACCCAGATAAGCATCTTGCACCCCTCACACTTGTATATACGGAGCAAACTACATAATCTCTA
AACAGAAAAAAAAAAAAAGAAAGAAAATTAAGAACATTACTTTTTCCGAGCAACAAACGCAAAAATATATGAAGTGGCTGTAGAACA
TGCCTTACGGAAGAAAAGAATGATGATGGCATCCCCTTCCCCTGCCCTGGAAGGTGGCCTGTATTTCTGTTCTTTTTTTATTTTTGT
TCGCGCAAAGAGCAAGAACATTGGTTGCTTTACTTATTATTTTCAGGGTCGCTTCGCATTCTGCGCCTTCGAGCACAGGATAAGTTG
CAGGAAGCCATCACATCTATGCAACGATTATCACGACACAACCTTGCCGCCGAGAAAACGTCCGTGGAGAACCATTCGGTCGATTGC
TTCCCACGGAACGAGTGGACTGAAACTTAAGACTGCCCCTCTCTTTCCGCGGAATCGCTCATGCCGCGGTGCCACAACATTTTCAGA
TTTACTAAGACTCCGGTGAGTGTGGGCTCACCCGCGGGTCGTGATCACGATTCAGCACCCTTTGGACTCGTGATTCCGTGGAAAGGT
CAGATCTGTATTCCTACTTATGGTAATGTGCTAAAAAAAGAGAAATGTCTCCGCGGAACTCTTCTACGCCGTGGTACGATATCTGTT
GAACGTAATCTGAGCAATACAAACAAGGCCTCTCCTATACATATATAATTGTGATGTGCATAACCTTATGGCTGTTCGCTTTTATTA
CCATACCTTAGAATGAAATCCAAAAGAAAAAAGTCACGCAAAGTTGCAAACATATAACAACTGTGTTAGTTATCACTCGACTTTGTT
ATTCTAATTATAAATAAATTGGCAACTAGGAACTTTCGAAAAAGAAATTAAAGACCCTTTTAAGTTTTCGTATCCGCTCGTTCGAAA
GACTTAATTAAAAAATGGCACAATTAGATACACTCGATATCGTGGTTTTGGTAGTTCTTCTTGTCGGTTCTGTCGCTTACTTTACTA
AGGGTTCCTATTGGGCTGTACCAAAGGACCCATACGCTGCAGCAAACTCCGCCATGAATGGTGCAGCTAAGACAGGTAAGACAAGAG
ACATTATCCAAAAAATGGAAGAGACTGGTAAAAACTGCGTGATATTCTACGGTTCACAGACAGGAACAGCCGAAGATTACGCTTCAA
GACTGGCCAAAGAGGGCTCACAGAGATTTGGCCTTAAGACTATGGTCGCGGACCTTGAGGATTACGATTACGAGAACTTAGACAAGT
TTCCTGAGGATAAGATTGCATTTTTCGTCCTGGCTACCTACGGCGAGGGTGAACCAACCGACAATGCAGTCGAATTCTACCAATTCA
TTACTGGAGAGGATGTAGCTTTTGAATCTGGAGCTTCAGCAGAAGAGAAGCCACTGTCCTCCTTGAAATACGTTGCCTTCGGCCTCG
GCAATAACACTTATGAACACTACAACGCAATGGTAAGACATGTTGATGCGGCACTAACCAAGTTGGGGGCACAACGTATCGGAACTG
CCGGTGAGGGCGACGATGGCGCTGGAACAATGGAAGAGGATTTTCTAGCTTGGAAGGAACCAATGTGGGCAGCTTTAAGCGAATCTA
TGAATCTACAAGAGAGAGAAGCCGTGTACGAACCAGTATTCAGTGTCATCGAAGATGAATCTCTTAGTCCAGAAGATGATTCTGTGT
ACCTCGGTGAACCTACTCAAGGGCATTTATCTGGTTCACCAAAGGGCCCTTACTCAGCTCATAATCCTTACATAGCACCAATTGTCG
AATCTAGAGAATTGTTCACTGCCAAGGACAGAAATTGTTTGCATATGGAAATTGGAATTGCTGGTTCTAACTTGACTTATCAGACAG
GTGACCACATCGCAATTTGGCCAACCAATGCCGGTGTTGAAGTTGACAGATTCTTGGAAGTGTTTGGGATCGAAAAgAAGAGACATA
CTGTCATTAACATAAAGGGATTAGATGTTACTGCGAAAGTTCCTATTCCAACACCAACAACATACGACGCTGCAGTACGATTCTACA
TGGAAATATGTGCTCCTGTGTCTAGACAATTTGTGTCATCACTTGTTCCATTCGCACCAGATGAAGAGTCCAAAGCCGAAATTGTTA
GATTGGGGAACGATAAAGATTACTTCCACGAAAAGATATCAAATCAGTGTTTTAACATCGCCCAAGCCCTGCAAAACATTACGTCTA
AGCCATTCAGTGCAGTTCCTTTTTCATTATTGATCGAAGGTTTAAACAGATTGCAACCAAGATATTACTCTATTAGTTCTAGCTCAC
TAGTTCAGAAAGATAAGATTTCAATTACAGCGGTTGTTGAGTCAGTTAGATTACCAGGCGCAAGTCATATTGTTAAGGGTGTTACAA
CCAACTACCTATTAGCATTGAAGCAAAAGCAAAACGGTGATCCTTCTCCAGATCCACATGGATTAACATACGCTATCACAGGGCCAC
GTAACAAATACGATGGTATCCACGTCCCTGTGCATGTTAGACATTCTAACTTCAAGCTTCCATCTGACCCATCGAAGCCAATCATAA
TGGTCGGGCCTGGTACTGGCGTAGCACCTTTTAGAGGTTTCATTCAAGAGAGAGCTGCGCTAGCAGAATCTGGTAAGGATGTTGGTC

CTACAATCTTATTCTTCGGTTGCAGAAATAGAAATGAAGATTTCCTATACAAAGAGGAATGGAAAGTTTACCAGGAAAAGCTTGGTG
ATAAGTTGAAGATTATCACAGCCTTCTCCaGaGAAACCGCTAAAAAAGTTTACGTTCAACATAGGTTGCAGGAACACGCTGACTTGG
TGTCAGACCTACTCAAACAAAAAGCAACCTTCTACGTTTGCGGTGATGCTGCTAACATGGCAAGAGAAGTGAATTTAGTTTTGGGTC
AAATAATCGCTAAATCTAGAGGCTTGCCAGCAGAAAAGGGTGAAGAGATGGTCAAGCACATGCGTTCAAGTGGATCTTACCAAGAGG
ATGTATGGTCCGGCGGCCGCCATCATCACCATCATCATTAAATTCGGCCGCTTCTTTGGAATTATTGGAAGGTAAGGAATTGCCAGG
TGTTGCTTTCTTATCCGAAAAGAAATAAATTGAATTGAATTGAAATCGATAGATCAATTTTTTTCTTTTCTCTTTCCCCATCCTTTA
CGCTAAAATAATAGTTTATTTTATTTTTTGAATATTTTTTATTTATATACGTATATATAGACTATTATTTATCTTTTAATGATTATT
AAGATTTTTATTAAAAAAAAATTCGCTCCTCTTTTAATGCCTTTATGCAGTTTTTTTTTCCCATTCGATATTTCTATGTTCGGGTTC
AGCGTATTTTAAGTTTAATAACTCGAAAATTCTGCGTTCGTTGGAGAAGCTTCGTACGCTGCAGGTCGACAACCCTTAATATAACTT
CGTATAatgtatgcTATACGAAGTTATTAGGTCTAGAGATCTGTTTAGCTTGCCTCGTCCCCGCCGGGTCACCCGGCCAGCGACATG
GAGGCCCAGAATACCCTCCTTGACAGTCTTGACGTGCGCAGCTCAGGGGCATGATGTGACTGTCGCCCGTACATTTAGCCCATACAT
CCCCATGTATAATCATTTGCATCCATACATTTTGATGGCCGCACGGCGCGAAGCAAAAATTACGGCTCCTCGCTGCAGACCTGCGAG
CAGGGAAACGCTCCCCTCACAGACGCGTTGAATTGTCCCCACGCCGCGCCCCTGTAGAGAAATATAAAAGGTTAGGATTTGCCACTG
AGGTTCTTCTTTCATATACTTCCTTTTAAAATCTTGCTAGGATACAGTTCTCACATCACATCCGAACATAAACAACC**ATGGATTTGG**
TGAACCATCTAACCGATAGACTACTGTTTGCAATCCCAAAGAAAGGTCGTTTATATTCTAAAAGTGTTTCTATTTTGAATGGTGCTG
ATATTACCTTTCACCGCTCTCAAAGATTAGACATTGCACTAAGCACAAGCTTACCTGTAGCGTTGGTCTTTCTGCCCGCTGCAGATA
TTCCAACTTTTGTTGGTGAAGGTAAATGTGATCTTGGTATAACTGGTGTTGACCAAGTTCGTGAATCTAACGTCGACGTAGACTTAG
CAATCGATTTGCAATTTGGTAACTGTAAATTGCAGGTACAAGTCCCCGTAAATGGCGAGTATAAAAAGCCAGAACAGTTAATTGGCA
AAACCATTGTTACCAGTTTCGTGAAACTTGCTGAAAAATACTTTGCCGATTTGGAAGGTACTACTGTTGAAAAAATGACCACAAGGA
TAAAGTTTGTCAGTGGTTCCGTGGAGGCATCATGTGCTCTGGGAATTGGTGATGCTATTGTAGATCTTGTAGAGAGTGGTGAGACAA
TGAGGGCAGCAGGTTTAGTTGATATTGCCACCGTCCTAAGCACAAGTGCCTACCTAATAGAATCAAAGAACCCAAAGAGCGATAAGA
GTTTGATTGCTACTATCAAATCAAGAATTGAAGGTGTCATGACCGCTCAAAGGTTCGTTTCATGTATTTATAACGCACCTGAAGACA
AGCTGCCTGAACTGTTGAAGGTGACGCCTGGCCGTAGAGCACCAACCATTTCCAAAATTGACGATGAAGGATGGGTTGCTGTTAGTT
CCATGATTGAGAGAAAAACGAAGGGTGTTGTTTTAGATGAATTGAAAAGACTCGGCGCATCTGATATCATGGTTTTCGAAATTTCTA
ATTGTCGTGTATAATCAGTACTGACAATAAAAAGATTCTTGTTTTCAAGAACTTGTCATTTGTATAGTTTTTTTATATTGTAGTTGT
TCTATTTTAATCAAATGTTAGCGTGATTTATATTTTTTTTCGCCTCGACATCATCTGCCCAGATGCGAAGTTAAGTGCGCAGAAAGT
AATATCATGCGTCAATCGTATGTGAATGCTGGTCGCTATACTGCTGTCGATTCGATACTAACGCCGCCATCCAGTGTCGAAAACGAG
CTCTCGAGAACCCTTAATATAACTTCGTATAatgtatgcTATACGAAGTTATTAGGTGATATCAGATCCACTAGTGGCCTATGCGGT
GACGTTATTTTCCTTTTTTTTAGTTATATTATCTTTTTATTATATTCTATTCTCTCTACTGGGGAGAAATTACTTAATTTTCATTTA
TATTAAAATAATACCTACTGTTTCATACTTTCACTTTAACCACCATTTCTTTAAGCTAAAGTTTAGGAACGCTAAACATATCATTGT
TTACTTTTCGAGGAAACATAACTTTCAACATCAGGCTCTTTTCGTTTTATATTATCTATATTCTATATAGTAGTTTATAGAAATGCA
TTTAACACTAAATAACAGACAAGATAACGTTTTCAGAGTCGAACTGGATTATTCCATC

Sequence of *AfErg6xFLAG* cassette in *S. cerevisiae ERG11* locus:

***ERG11::AfErg6xFLAG-LoxP_His1* (ScErg11_Up773/ScErg11-down)**

The forward ScErg11_Up773 priming site at the 5' end of the DNA sequence and the reverse ScErg11-down priming site at the 3' end of the DNA sequence is highlighted in grey. The codon optimised *AfErg6xFLAG* ORF is highlighted in yellow. The *His1* selective marker is in bold between LoxP sites highlighted in red.

GCAACAATGGGCGGTTGTTTAGAGAGGTTTTGTGCCGCGCCCGGGAATTACCGGGGCACAGCAAAATACGAAATTTCCGGCAAAAT
GTCTCTGTACGAAAATGAGGCCAGCCTTTATTCCCGACTAAGCCGTACGATTATAGTAATGTCACACGAAGTGGATACTATACGAGC
AGCGCACATACAATGTGCGTGCAAGATTTGCCGGGTTGGACAATCTTAAAGGCCGATAATCCACGATCGCATTCTGCGCCTTCGAGC
ACAGGATAAGTTGCAGGAAGCCATCACATCTATGCAACGATTATCACGACACAACCTTGCCGCCGAGAAAACGTCCGTGGAGAACCA
TTCGGTCGATTGCTTCCCACGGAACGAGTGGACTGAAACTTAAGACTGCCCCTCTCTTTCCGCGGAATCGCTCATGCCGCGGTGCCA
CAACATTTTCAGATTTACTAAGACTCCGGTGAGTGTGGGCTCACCCGCGGGTCGTGATCACGATTCAGCACCCTTTGGACTCGTGAT
TCCGTGGAAAGGTCAGATCTGTATTCCTACTTATGGTAATGTGCTAAAAAAAGAGAAATGTCTCCGCGGAACTCTTCTACGCCGTGG
TACGATATCTGTTGAACGTAATCTGAGCAATACAAACAAGGCCTCTCCTATACATATATAATTGTGATGTGCATAACCTTATGGCTG
TTCGCTTTTATTACCATACCTTAGAATGAAATCCAAAAGAAAAAAGTCACGCAAAGTTGCAAACATATAACAACTGTGTTAGTTATC
ACTCGACTTTGTTATTCTAATTATAAATAAATTGGCAACTAGGAACTTTCGAAAAAGAAATTAAAGACCCTTTTAAGTTTTCGTATC
CGCTCGTTCGAAAGACTTAATTAAAAAATGGCACCAGTAGCCTTGGAACAGGAAAATCATCTCAGAGATGCAGAATTCAACAGAGCT
ATGCATGGTAAATCAGCACAATTTCGTGGTGGCTTCGCGGCACTGAGAGGAAAAGATTCCGCTGCTCAAAAAGCTGCAGTTGACGAA
TATTTCAAGCATTGGGACAATAAGCCAGCTGAAGATGAAACTGAAGAGACAAGAGCCGCCAGAAGGGCAGAATATGCCACATTAACC
AGACACTACTATAATCTGGCGACTGATCTTTACGAATACGGTTGGGGAACATCTTTCCACTTTTGTCGTTTTGCTCAGGGTGAACCA
TTCTACCAAGCCATTGCAAGACATGAACACTACTTAGCACACCAAATGGGCATTAAAGAGGGCATGAAAGTTCTTGATGTCGGTTGC
GGTGTTGGTGGACCAGCAAGAGAAATAGTTAAGTTCACAGATGCTAACGTCGTCGGATTGAACAACAACGATTACCAAATCGAGAGA
GCTACAAGATACGCCGAGAGAGAGGGACTCAGCCATAAGTTGAGTTTTGTTAAGGGTGATTTCATGCAGATGAAATTTCCTGATAAC
TCATTTGATGCAGTTTACGCTATCGAAGCAACAGTTCATGCACCAGACCTTGAGGGTGTGTACAAAGAGATTTTCAGGGTCCTAAAG
CCTGGTGGAGTGTTCGGGGTGTACGAATGGCTTATGACAGATGCTTACGATAACGATAACCCAGAACATCGTAGAATTAGACTGGGT
ATCGAATTGGGGGACGGGATCTCCAATATGGTAAAGGTATCAGAGGGCTTGACAGCTTTCAAAAACGCTGGTTTCGAACTACTACAT
AATGAAGATTTGGCGGATAGACCAGACGCTATACCATGGTACTACCCTTTAGCCGGATCTTTTAAGCACATGACTTCTCCATGGGAC
TTTTTCACCATCGCTAGAATGACCTGGTGGGGTAGAGGCATCGCACATAGATTCTGCGGCGCAATGGAAACTATTGGCTTATTCCCT
AAGGGTACTCAAAAGACTGCTGACTCTTTAGCAATTGCGGGTGATTGTTTAGTTGCCGGTGGGGAGAAAAAGTTGTTCACGCCAATG
TACTTGATGGTTGGTAGAAAGCCTGAGGGCggccgcGACTACAAAGACGATGACGACAAGTAATGGGTGGTATATATATATATATAT
ATATATATAACTGTCTAGAAATAAAGAGTATCATCTTTCAAAGAAGCTTCGTACGCTGCAGGTCGACAACCCTTAATATAACTTCGT
ATAatgtatgcTATACGAAGTTATTAGGTCTAGAGATCTGTTTAGCTTGCCTCGTCCCCGCCGGGTCACCCGGCCAGCGACATGGAG
GCCCAGAATACCCTCCTTGACAGTCTTGACGTGCGCAGCTCAGGGGCATGATGTGACTGTCGCCCGTACATTTAGCCCATACATCCC
CATGTATAATCATTTGCATCCATACATTTTGATGGCCGCACGGCGCGAAGCAAAAATTACGGCTCCTCGCTGCAGACCTGCGAGCAG
GGAAACGCTCCCCTCACAGACGCGTTGAATTGTCCCCACGCCGCGCCCCTGTAGAGAAATATAAAAGGTTAGGATTTGCCACTGAGG
TTCTTCTTTCATATACTTCCTTTTAAAATCTTGCTAGGATACAGTTCTCACATCACATCCGAACATAAACAACC**ATGGATTTGGTGA**
ACCATCTAACCGATAGACTACTGTTTGCAATCCCAAAGAAAGGTCGTTTATATTCTAAAAGTGTTTCTATTTTGAATGGTGCTGATA
TTACCTTTCACCGCTCTCAAAGATTAGACATTGCACTAAGCACAAGCTTACCTGTAGCGTTGGTCTTTCTGCCCGCTGCAGATATTC

**CAACTTTTGTTGGTGAAGGTAAATGTGATCTTGGTATAACTGGTGTTGACCAAGTTCGTGAATCTAACGTCGACGTAGACTTAGCAA
TCGATTTGCAATTTGGTAACTGTAAATTGCAGGTACAAGTCCCCGTAAATGGCGAGTATAAAAAGCCAGAACAGTTAATTGGCAAAA
CCATTGTTACCAGTTTCGTGAAACTTGCTGAAAAATACTTTGCCGATTTGGAAGGTACTACTGTTGAAAAAATGACCACAAGGATAA
AGTTTGTCAGTGGTTCCGTGGAGGCATCATGTGCTCTGGGAATTGGTGATGCTATTGTAGATCTTGTAGAGAGTGGTGAGACAATGA
GGGCAGCAGGTTTAGTTGATATTGCCACCGTCCTAAGCACAAGTGCCTACCTAATAGAATCAAAGAACCCAAAGAGCGATAAGAGTT
TGATTGCTACTATCAAATCAAGAATTGAAGGTGTCATGACCGCTCAAAGGTTCGTTTCATGTATTTATAACGCACCTGAAGACAAGC
TGCCTGAACTGTTGAAGGTGACGCCTGGCCGTAGAGCACCAACCATTTCCAAAATTGACGATGAAGGATGGGTTGCTGTTAGTTCCA
TGATTGAGAGAAAAACGAAGGGTGTTGTTTTAGATGAATTGAAAAGACTCGGCGCATCTGATATCATGGTTTTCGAAATTTCTAATT
GTCGTGTATAA**TCAGTACTGACAATAAAAAGATTCTTGTTTTCAAGAACTTGTCATTTGTATAGTTTTTTTATATTGTAGTTGTTCT
ATTTTAATCAAATGTTAGCGTGATTTATATTTTTTTTCGCCTCGACATCATCTGCCCAGATGCGAAGTTAAGTGCGCAGAAAGTAAT
ATCATGCGTCAATCGTATGTGAATGCTGGTCGCTATACTGCTGTCGATTCGATACTAACGCCGCCATCCAGTGTCGAAAACGAGCTC
TCGAGAACCCTTAATATAACTTCGTATAatgtatgcTATACGAAGTTATTAGGTGATATCAGATCCACTAGTGGCCTATGCGGCCGG
CCTTATCGCCACACTCATCCATCTCCAATGATTTATTTTATTTGCCAGTACGTTTGATATGACATCGAGTTATGTTCTTTAGTCACT
TTCAAACTTGGACAACCATCCCGCATTATTTATGAGTGGTATGGTTTCACTTCTTTATTTATAAATATTTATACTACGAAATTATAT
ATAGATTACATTTCTAAAACAACATCAAATATAAGGTTTATTTATGCAACATGCCTACTGGATTAGAAGAGAGAACACACAGCTCAC
AGGCCAAGCAGAAATAAAGCAGTCTTTTCGCAAACTTGTAAACCTTTTTGCAATTTAAATTAATCAAGAGAGGGCCCAGGAGGAGAG
GAGTTTTGGGGAGGGGGGGGTCACTGAGGAAGCTCTCTTAAATATGGATTCACTGAAATAAATGTATATAAGATTCATTACTGCTTT
TTATAGTGATAAATCCAAATGATGAGTGAATTGAACCAT

Sequence of *AfCyp51A-6×His* (mutation Y121F) cassette in *S. cerevisiae PDR5* locus:*PDR5::AfCyp51A-6×His-LoxP_His1* (mutation Y121F in AfCYP51A) (PDR5Fv3 / PDR5_186DS_R)

The *AfCyp51A-6×His* codon optimised open reading frame (ORF) is highlighted in yellow. The desire mutation TAC → TTC is highlighted in pink. The *His1* selective marker is in bold between LoxP sites highlighted in red.

TCGCATTCTGCGCCTTCGAGCACAGGATAAGTTGCAGGAAGCCATCACATCTATGCAACGATTATCACGACACAACCTTGCCGCCGA

GAAAACGTCCGTGGAGAACCATTCGGTCGATTGCTTCCCACGGAACGAGTGGACTGAAACTTAAGACTGCCCCTCTCTTTCCGCGGA

ATCGCTCATGCCGCGGTGCCACAACATTTTCAGATTTACTAAGACTCCGGTGAGTGTGGGCTCACCCGCGGGTCGTGATCACGATTC

AGCACCCTTTGGACTCGTGATTCCGTGGAAAGGTCAGATCTGTATTCCTACTTATGGTAATGTGCTAAAAAAAGAGAAATGTCTCCG

CGGAACTCTTCTACGCCGTGGTACGATATCTGTTGAACGTAATCTGAGCAATACAAACAAGGCCTCTCCTATACATATATAATTGTG

ATGTGCATAACCTTATGGCTGTTCGCTTTTATTACCATACCTTAGAATGAAATCCAAAAGAAAAAAGTCACGCAAAGTTGCAAACAT

ATAACAACTGTGTTAGTTATCACTCGACTTTGTTATTCTAATTATAAATAAATTGGCAACTAGGAACTTTCGAAAAAGAAATTAAAG

ACCCTTTTAAGTTTTCGTATCCGCTCGTTCGAAAGACTTAATTAAAAAATGGTCCCTATGCTCTGGTTGACCGCATACATGGCAGTT

GCAGTTCTCACAGCCATCTTACTAAACGTAGTTTATCAGTTGTTTTTCCGTTTGTGGAACAGAACAGAACCTCCAATGGTGTTCCAT

TGGGTGCCATACCTGGGCTCAACCATTTCATACGGTATTGATCCATACAAGTTCTTCTTCGCTTGTAGAGAGAAATACGGTGACATT

TTCACATTCATTCTACTGGGACAAAAGACAACAGTTTACCTGGGTGTTCAAGGTAATGAATTCATACTGAATGGCAAGTTAAAAGAC

GTCAATGCCGAAGAGGTTTATTCTCCATTGACAACACCAGTTTTCGGCTCCGATGTTGTT**TTC**GATTGCCCTAACTCTAAGCTTATG

GAACAGAAAAAGTTTATCAAGTACGGTCTTACCCAATCAGCCTTGGAATCACATGTCCCTTTAATCGAGAAGGAAGTGCTTGACTAC

CTAAGAGATAGCCCTAACTTTCAAGGTTCTAGTGGTAGAGTCGATATTTCTGCAGCAATGGCAGAAATCACAATCTTTACTGCGGCT

AGAGCTTTACAAGGTCAAGAGGTAAGATCTAAATTGACTGCAGAATTTGCTGACTTGTACCACGATTTGGATAAAGGTTTCACTCCA

ATCAACTTTATGCTACCATGGGCCCCATTACCACACAACAAAAAGAGAGATGCTGCTCATGCTAGAATGAGATCAATCTATGTTGAC

ATTATCACCCAGAGAAGATTAGACGGGGAAAAAGACAGCCAAAAGTCTGATATGATTTGGAATTTGATGAATTGTACATACAAAAAT

GGTCAACAGGTACCTGATAAGGAGATTGCACATATGATGATCACACTTTTGATGGCCGGACAACATTCTTCTTCCTCTATATCAGCA

TGGATTATGTTGAGATTAGCTTCTCAACCAAAGGTATTAGAAGAGTTGTACCAAGAGCAACTCGCGAACTTAGGTCCTGCGGGTCCA

GATGGATCATTACCACCATTGCAATACAAGGACCTTGATAAGCTTCCTTTTCATCAACATGTCATTAGAGAAACTTTGAGAATTCAT

AGTAGTATCCACTCTATAATGAGAAAGGTCAAATCCCCATTACCTGTCCCAGGTACTCCATACATGATACCACCAGGCAGAGTTCTT

CTAGCATCACCTGGTGTAACTGCCCTGTCAGATGAGCATTTCCCAAACGCTGGCTGTTGGGATCCACATAGATGGGAAAACCAGGCC

ACCAAGGAACAGGAAAATGATAAGGTGGTTGATTACGGATATGGAGCTGTGTCAAAGGGTACTTCCAGCCCATACCTCCCATTTGGG

GCAGGACGTCACAGATGCATCGGAGAAAAGTTCGCTTACGTCAACTTAGGTGTTATCTTGGCTACAATAGTGAGACATTTGCGTCTA

TTCAACGTTGATGGGAAAAAGGGTGTTCCAGAAACAGATTACTCTTCTTTGTTCAGTGGTCCTATGAAGCCTTCAATTATCGGCTGG

GAAAAGAGATCTAAAAACACTTCCAAAGGCGGCCGCCATCATCACCATCATCATTAAATTCGGCCGCTTCTTTGGAATTATTGGAAG

GTAAGGAATTGCCAGGTGTTGCTTTCTTATCCGAAAAGAAATAAATTGAATTGAATTGAAATCGATAGATCAATTTTTTTCTTTTCT

CTTTCCCCATCCTTTACGCTAAAATAATAGTTTATTTTATTTTTTGAATATTTTTTATTTATATACGTATATATAGACTATTATTTA

TCTTTTAATGATTATTAAGATTTTTATTAAAAAAAAATTCGCTCCTCTTTTAATGCCTTTATGCAGTTTTTTTTTCCCATTCGATAT

TTCTATGTTCGGGTTCAGCGTATTTTAAGTTTAATAACTCGAAAATTCTGCGTTCGTTGGAGAAGCTTCGTACGCTGCAGGTCGACA

ACCCTTAATATAACTTCGTATAatgtatgcTATACGAAGTTATTAGGTCTAGAGATCTGTTTAGCTTGCCTCGTCCCCGCCGGGTCA

CCCGGCCAGCGACATGGAGGCCCAGAATACCCTCCTTGACAGTCTTGACGTGCGCAGCTCAGGGGCATGATGTGACTGTCGCCCGTA

CATTTAGCCCATACATCCCCATGTATAATCATTTGCATCCATACATTTTGATGGCCGCACGGCGCGAAGCAAAAATTACGGCTCCTC

GCTGCAGACCTGCGAGCAGGGAAACGCTCCCCTCACAGACGCGTTGAATTGTCCCCACGCCGCGCCCCTGTAGAGAAATATAAAAGG
TTAGGATTTGCCACTGAGGTTCTTCTTTCATATACTTCCTTTTAAAATCTTGCTAGGATACAGTTCTCACATCACATCCGAACATAA
ACAACC**ATGGATTTGGTGAACCATCTAACCGATAGACTACTGTTTGCAATCCCAAAGAAAGGTCGTTTATATTCTAAAAGTGTTTCT**
ATTTTGAATGGTGCTGATATTACCTTTCACCGCTCTCAAAGATTAGACATTGCACTAAGCACAAGCTTACCTGTAGCGTTGGTCTTT
CTGCCCGCTGCAGATATTCCAACTTTTGTTGGTGAAGGTAAATGTGATCTTGGTATAACTGGTGTTGACCAAGTTCGTGAATCTAAC
GTCGACGTAGACTTAGCAATCGATTTGCAATTTGGTAACTGTAAATTGCAGGTACAAGTCCCCGTAAATGGCGAGTATAAAAAGCCA
GAACAGTTAATTGGCAAAACCATTGTTACCAGTTTCGTGAAACTTGCTGAAAAATACTTTGCCGATTTGGAAGGTACTACTGTTGAA
AAAATGACCACAAGGATAAAGTTTGTCAGTGGTTCCGTGGAGGCATCATGTGCTCTGGGAATTGGTGATGCTATTGTAGATCTTGTA
GAGAGTGGTGAGACAATGAGGGCAGCAGGTTTAGTTGATATTGCCACCGTCCTAAGCACAAGTGCCTACCTAATAGAATCAAAGAAC
CCAAAGAGCGATAAGAGTTTGATTGCTACTATCAAATCAAGAATTGAAGGTGTCATGACCGCTCAAAGGTTCGTTTCATGTATTTAT
AACGCACCTGAAGACAAGCTGCCTGAACTGTTGAAGGTGACGCCTGGCCGTAGAGCACCAACCATTTCCAAAATTGACGATGAAGGA
TGGGTTGCTGTTAGTTCCATGATTGAGAGAAAAACGAAGGGTGTTGTTTTAGATGAATTGAAAAGACTCGGCGCATCTGATATCATG
GTTTTCGAAATTTCTAATTGTCGTGTATAATCAGTACTGACAATAAAAAGATTCTTGTTTTCAAGAACTTGTCATTTGTATAGTTTT
TTTATATTGTAGTTGTTCTATTTTAATCAAATGTTAGCGTGATTTATATTTTTTTTCGCCTCGACATCATCTGCCCAGATGCGAAGT
TAAGTGCGCAGAAAGTAATATCATGCGTCAATCGTATGTGAATGCTGGTCGCTATACTGCTGTCGATTCGATACTAACGCCGCCATC
CAGTGTCGAAAACGAGCTCTCGAGAACCCTTAATATAACTTCGTATAatgtatgcTATACGAAGTTATTAGAATTTTGAATTTGGTT
AAGAAAAGAAACTTACCAAGATGGACTTTTTTAATAAACATACATAATCACTACATATAGGTGCGTAATAATAAGTTTTTATTTTTT
TTTTCTTAATTCAGCGAGCTTTCTACATTTCAATTTTCTTGAATTTACCAGCTCTGATAAATCAAAGTTCAATGTCCGAA

Sequence of *AfCyp51A-6×His* (mutation T289A) cassette in *S. cerevisiae PDR5* locus: *PDR5::AfCyp51A-6×His-LoxP_His1* (mutation T289A in AfCYP51A) (PDR5Fv3 / PDR5_186DS_R)

The codon optimised *AfCyp51A-6×His* open reading frame (ORF) is highlighted in yellow. The desired mutation ACA → GCT is highlighted in pink. The *His1* selective marker is in bold between LoxP sites highlighted in red.

TCGCATTCTGCGCCTTCGAGCACAGGATAAGTTGCAGGAAGCCATCACATCTATGCAACGATTATCACGACACAACCTTGCCGCCGA
GAAAACGTCCGTGGAGAACCATTCGGTCGATTGCTTCCCACGGAACGAGTGGACTGAAACTTAAGACTGCCCCTCTCTTTCCGCGGA
ATCGCTCATGCCGCGGTGCCACAACATTTTCAGATTTACTAAGACTCCGGTGAGTGTGGGCTCACCCGCGGGTCGTGATCACGATTC
AGCACCCTTTGGACTCGTGATTCCGTGGAAAGGTCAGATCTGTATTCCTACTTATGGTAATGTGCTAAAAAAAGAGAAATGTCTCCG
CGGAACTCTTCTACGCCGTGGTACGATATCTGTTGAACGTAATCTGAGCAATACAAACAAGGCCTCTCCTATACATATATAATTGTG
ATGTGCATAACCTTATGGCTGTTCGCTTTTATTACCATACCTTAGAATGAAATCCAAAAGAAAAAAGTCACGCAAAGTTGCAAACAT
ATAACAACTGTGTTAGTTATCACTCGACTTTGTTATTCTAATTATAAATAAATTGGCAACTAGGAACTTTCGAAAAAGAAATTAAAG
ACCCTTTTAAGTTTTCGTATCCGCTCGTTCGAAAGACTTAATTAAAAAATGGTCCCTATGCTCTGGTTGACCGCATACATGGCAGTT
GCAGTTCTCACAGCCATCTTACTAAACGTAGTTTATCAGTTGTTTTTCCGTTTGTGGAACAGAACAGAACCTCCAATGGTGTTCCAT
TGGGTGCCATACCTGGGCTCAACCATTTCATACGGTATTGATCCATACAAGTTCTTCTTCGCTTGTAGAGAGAAATACGGTGACATT
TTCACATTCATTCTACTGGGACAAAAGACAACAGTTTACCTGGGTGTTCAAGGTAATGAATTCATACTGAATGGCAAGTTAAAAGAC
GTCAATGCCGAAGAGGTTTATTCTCCATTGACAACACCAGTTTTCGGCTCCGATGTTGTTTACGATTGCCCTAACTCTAAGCTTATG
GAACAGAAAAAGTTTATCAAGTACGGTCTTACCCAATCAGCCTTGGAATCACATGTCCCTTTAATCGAGAAGGAAGTGCTTGACTAC
CTAAGAGATAGCCCTAACTTTCAAGGTTCTAGTGGTAGAGTCGATATTTCTGCAGCAATGGCAGAAATCACAATCTTTACTGCGGCT
AGAGCTTTACAAGGTCAAGAGGTAAGATCTAAATTGACTGCAGAATTTGCTGACTTGTACCACGATTTGGATAAAGGTTTCACTCCA
ATCAACTTTATGCTACCATGGGCCCCATTACCACACAACAAAAAGAGAGATGCTGCTCATGCTAGAATGAGATCAATCTATGTTGAC
ATTATCACCCAGAGAAGATTAGACGGGGAAAAAGACAGCCAAAAGTCTGATATGATTTGGAATTTGATGAATTGTACATACAAAAAT
GGTCAACAGGTACCTGATAAGGAGATTGCACATATGATGATCGCTCTTTTGATGGCCGGACAACATTCTTCTTCCTCTATATCAGCA
TGGATTATGTTGAGATTAGCTTCTCAACCAAAGGTATTAGAAGAGTTGTACCAAGAGCAACTCGCGAACTTAGGTCCTGCGGGTCCA
GATGGATCATTACCACCATTGCAATACAAGGACCTTGATAAGCTTCCTTTTCATCAACATGTCATTAGAGAAACTTTGAGAATTCAT
AGTAGTATCCACTCTATAATGAGAAAGGTCAAATCCCCATTACCTGTCCCAGGTACTCCATACATGATACCACCAGGCAGAGTTCTT
CTAGCATCACCTGGTGTAACTGCCCTGTCAGATGAGCATTTCCCAAACGCTGGCTGTTGGGATCCACATAGATGGGAAAACCAGGCC
ACCAAGGAACAGGAAAATGATAAGGTGGTTGATTACGGATATGGAGCTGTGTCAAAGGGTACTTCCAGCCCATACCTCCCATTTGGG
GCAGGACGTCACAGATGCATCGGAGAAAAGTTCGCTTACGTCAACTTAGGTGTTATCTTGGCTACAATAGTGAGACATTTGCGTCTA
TTCAACGTTGATGGGAAAAAGGGTGTTCCAGAAACAGATTACTCTTCTTTGTTCAGTGGTCCTATGAAGCCTTCAATTATCGGCTGG
GAAAAGAGATCTAAAAACACTTCCAAAGGCGGCCGCCATCATCACCATCATCATTAAATTCGGCCGCTTCTTTGGAATTATTGGAAG
GTAAGGAATTGCCAGGTGTTGCTTTCTTATCCGAAAAGAAATAAATTGAATTGAATTGAAATCGATAGATCAATTTTTTTCTTTTCT
CTTTCCCCATCCTTTACGCTAAAATAATAGTTTATTTTATTTTTTGAATATTTTTTATTTATATACGTATATATAGACTATTATTTA
TCTTTTAATGATTATTAAGATTTTTATTAAAAAAAAATTCGCTCCTCTTTTAATGCCTTTATGCAGTTTTTTTTTCCCATTCGATAT
TTCTATGTTCGGGTTCAGCGTATTTTAAGTTTAATAACTCGAAAATTCTGCGTTCGTTGGAGAAGCTTCGTACGCTGCAGGTCGACA
ACCCTTAATATAACTTCGTATAatgtatgcTATACGAAGTTATTAGGTCTAGAGATCTGTTTAGCTTGCCTCGTCCCCGCCGGGTCA
CCCGGCCAGCGACATGGAGGCCCAGAATACCCTCCTTGACAGTCTTGACGTGCGCAGCTCAGGGGCATGATGTGACTGTCGCCCGTA
CATTTAGCCCATACATCCCCATGTATAATCATTTGCATCCATACATTTTGATGGCCGCACGGCGCGAAGCAAAAATTACGGCTCCTC

GCTGCAGACCTGCGAGCAGGGAAACGCTCCCCTCACAGACGCGTTGAATTGTCCCCACGCCGCGCCCCTGTAGAGAAATATAAAAGG
TTAGGATTTGCCACTGAGGTTCTTCTTTCATATACTTCCTTTTAAAATCTTGCTAGGATACAGTTCTCACATCACATCCGAACATAA
ACAACC**ATGGATTTGGTGAACCATCTAACCGATAGACTACTGTTTGCAATCCCAAAGAAAGGTCGTTTATATTCTAAAAGTGTTTCT**
ATTTTGAATGGTGCTGATATTACCTTTCACCGCTCTCAAAGATTAGACATTGCACTAAGCACAAGCTTACCTGTAGCGTTGGTCTTT
CTGCCCGCTGCAGATATTCCAACTTTTGTTGGTGAAGGTAAATGTGATCTTGGTATAACTGGTGTTGACCAAGTTCGTGAATCTAAC
GTCGACGTAGACTTAGCAATCGATTTGCAATTTGGTAACTGTAAATTGCAGGTACAAGTCCCCGTAAATGGCGAGTATAAAAAGCCA
GAACAGTTAATTGGCAAAACCATTGTTACCAGTTTCGTGAAACTTGCTGAAAAATACTTTGCCGATTTGGAAGGTACTACTGTTGAA
AAAATGACCACAAGGATAAAGTTTGTCAGTGGTTCCGTGGAGGCATCATGTGCTCTGGGAATTGGTGATGCTATTGTAGATCTTGTA
GAGAGTGGTGAGACAATGAGGGCAGCAGGTTTAGTTGATATTGCCACCGTCCTAAGCACAAGTGCCTACCTAATAGAATCAAAGAAC
CCAAAGAGCGATAAGAGTTTGATTGCTACTATCAAATCAAGAATTGAAGGTGTCATGACCGCTCAAAGGTTCGTTTCATGTATTTAT
AACGCACCTGAAGACAAGCTGCCTGAACTGTTGAAGGTGACGCCTGGCCGTAGAGCACCAACCATTTCCAAAATTGACGATGAAGGA
TGGGTTGCTGTTAGTTCCATGATTGAGAGAAAAACGAAGGGTGTTGTTTTAGATGAATTGAAAAGACTCGGCGCATCTGATATCATG
GTTTTCGAAATTTCTAATTGTCGTGTATAATCAGTACTGACAATAAAAAGATTCTTGTTTTCAAGAACTTGTCATTTGTATAGTTTT
TTTATATTGTAGTTGTTCTATTTTAATCAAATGTTAGCGTGATTTATATTTTTTTTCGCCTCGACATCATCTGCCCAGATGCGAAGT
TAAGTGCGCAGAAAGTAATATCATGCGTCAATCGTATGTGAATGCTGGTCGCTATACTGCTGTCGATTCGATACTAACGCCGCCATC
CAGTGTCGAAAACGAGCTCTCGAGAACCCTTAATATAACTTCGTATAatgtatgcTATACGAAGTTATTAGAATTTTGAATTTGGTT
AAGAAAAGAAACTTACCAAGATGGACTTTTTTAATAAACATACATAATCACTACATATAGGTGCGTAATAATAAGTTTTTATTTTTT
TTTTCTTAATTCAGCGAGCTTTCTACATTTCAATTTTCTTGAATTTACCAGCTCTGATAAATCAAAGTTCAATGTCCGAA

Sequence of *AfCyp51A-6×His* (mutation Y121F T289A) cassette in *S. cerevisiae PDR5* locus:*PDR5::AfCyp51A-6×His-LoxP_His1* (mutation Y121F T289A in AfCYP51A) (PDR5Fv3 / PDR5_186DS_R)

The codon optimised *AfCyp51A-6×His* open reading frame (ORF) is highlighted in yellow. The desired mutations TAC→TTC and ACA → GCT are highlighted in pink. The *His1* selective marker is in bold between LoxP sites highlighted in red.

TCGCATTCTGCGCCTTCGAGCACAGGATAAGTTGCAGGAAGCCATCACATCTATGCAACGATTATCACGACACAACCTTGCCGCCGA
GAAAACGTCCGTGGAGAACCATTCGGTCGATTGCTTCCCACGGAACGAGTGGACTGAAACTTAAGACTGCCCCTCTCTTTCCGCGGA
ATCGCTCATGCCGCGGTGCCACAACATTTTCAGATTTACTAAGACTCCGGTGAGTGTGGGCTCACCCGCGGGTCGTGATCACGATTC
AGCACCCTTTGGACTCGTGATTCCGTGGAAAGGTCAGATCTGTATTCCTACTTATGGTAATGTGCTAAAAAAAGAGAAATGTCTCCG
CGGAACTCTTCTACGCCGTGGTACGATATCTGTTGAACGTAATCTGAGCAATACAAACAAGGCCTCTCCTATACATATATAATTGTG
ATGTGCATAACCTTATGGCTGTTCGCTTTTATTACCATACCTTAGAATGAAATCCAAAAGAAAAAGTCACGCAAAGTTGCAAACAT
ATAACAACTGTGTTAGTTATCACTCGACTTTGTTATTCTAATTATAAATAAATTGGCAACTAGGAACTTTCGAAAAAGAAATTAAAG
ACCCTTTTAAGTTTTCGTATCCGCTCGTTCGAAAGACTTAATTAAAAAATGGTCCCTATGCTCTGGTTGACCGCATACATGGCAGTT
GCAGTTCTCACAGCCATCTTACTAAACGTAGTTTATCAGTTGTTTTTCCGTTTGTGGAACAGAACAGAACCTCCAATGGTGTTCCAT
TGGGTGCCATACCTGGGCTCAACCATTTCATACGGTATTGATCCATACAAGTTCTTCTTCGCTTGTAGAGAGAAATACGGTGACATT
TTCACATTCATTCTACTGGGACAAAAGACAACAGTTTACCTGGGTGTTCAAGGTAATGAATTCATACTGAATGGCAAGTTAAAAGAC
GTCAATGCCGAAGAGGTTTATTCTCCATTGACAACACCAGTTTTCGGCTCCGATGTTGTT**TTC**GATTGCCCTAACTCTAAGCTTATG
GAACAGAAAAAGTTTATCAAGTACGGTCTTACCCAATCAGCCTTGGAATCACATGTCCCTTTAATCGAGAAGGAAGTGCTTGACTAC
CTAAGAGATAGCCCTAACTTTCAAGGTTCTAGTGGTAGAGTCGATATTTCTGCAGCAATGGCAGAAATCACAATCTTTACTGCGGCT
AGAGCTTTACAAGGTCAAGAGGTAAGATCTAAATTGACTGCAGAATTTGCTGACTTGTACCACGATTTGGATAAAGGTTTCACTCCA
ATCAACTTTATGCTACCATGGGCCCCATTACCACACAACAAAAAGAGAGATGCTGCTCATGCTAGAATGAGATCAATCTATGTTGAC
ATTATCACCCAGAGAAGATTAGACGGGGAAAAAGACAGCCAAAAGTCTGATATGATTTGGAATTTGATGAATTGTACATACAAAAAT
GGTCAACAGGTACCTGATAAGGAGATTGCACATATGATGATC**GCT**CTTTTGATGGCCGGACAACATTCTTCTTCCTCTATATCAGCA
TGGATTATGTTGAGATTAGCTTCTCAACCAAAGGTATTAGAAGAGTTGTACCAAGAGCAACTCGCGAACTTAGGTCCTGCGGGTCCA
GATGGATCATTACCACCATTGCAATACAAGGACCTTGATAAGCTTCCTTTTCATCAACATGTCATTAGAGAAACTTTGAGAATTCAT
AGTAGTATCCACTCTATAATGAGAAAGGTCAAATCCCCATTACCTGTCCCAGGTACTCCATACATGATACCACCAGGCAGAGTTCTT
CTAGCATCACCTGGTGTAACTGCCCTGTCAGATGAGCATTTCCCAAACGCTGGCTGTTGGGATCCACATAGATGGGAAAACCAGGCC
ACCAAGGAACAGGAAAATGATAAGGTGGTTGATTACGGATATGGAGCTGTGTCAAAGGGTACTTCCAGCCCATACCTCCCATTTGGG
GCAGGACGTCACAGATGCATCGGAGAAAAGTTCGCTTACGTCAACTTAGGTGTTATCTTGGCTACAATAGTGAGACATTTGCGTCTA
TTCAACGTTGATGGGAAAAAGGGTGTTCCAGAAACAGATTACTCTTCTTTGTTCAGTGGTCCTATGAAGCCTTCAATTATCGGCTGG
GAAAAGAGATCTAAAAACACTTCCAAAGGCGGCCGCCATCATCACCATCATCATTAAATTCGGCCGCTTCTTTGGAATTATTGGAAG
GTAAGGAATTGCCAGGTGTTGCTTTCTTATCCGAAAAGAAATAAATTGAATTGAATTGAAATCGATAGATCAATTTTTTTCTTTTCT
CTTTCCCCATCCTTTACGCTAAAATAATAGTTTATTTTATTTTTTGAATATTTTTTATTTATATACGTATATATAGACTATTATTTA
TCTTTTAATGATTATTAAGATTTTTATTAAAAAAAAATTCGCTCCTCTTTTAATGCCTTTATGCAGTTTTTTTTTCCCATTCGATAT
TTCTATGTTCGGGTTCAGCGTATTTTAAGTTTAATAACTCGAAAATTCTGCGTTCGTTGGAGAAGCTTCGTACGCTGCAGGTCGACA
ACCCTTAATATAACTTCGTATAatgtatgcTATACGAAGTTATTAGGTCTAGAGATCTGTTTAGCTTGCCTCGTCCCCGCCGGGTCA
CCCGGCCAGCGACATGGAGGCCCAGAATACCCTCCTTGACAGTCTTGACGTGCGCAGCTCAGGGGCATGATGTGACTGTCGCCCGTA

CATTTAGCCCATACATCCCCATGTATAATCATTTGCATCCATACATTTTGATGGCCGCACGGCGCGAAGCAAAAATTACGGCTCCTC
GCTGCAGACCTGCGAGCAGGGAAACGCTCCCCTCACAGACGCGTTGAATTGTCCCCACGCCGCGCCCCTGTAGAGAAATATAAAAGG
TTAGGATTTGCCACTGAGGTTCTTCTTTCATATACTTCCTTTTAAAATCTTGCTAGGATACAGTTCTCACATCACATCCGAACATAA
ACAACC**ATGGATTTGGTGAACCATCTAACCGATAGACTACTGTTTGCAATCCCAAAGAAAGGTCGTTTATATTCTAAAAGTGTTTCT
ATTTTGAATGGTGCTGATATTACCTTTCACCGCTCTCAAAGATTAGACATTGCACTAAGCACAAGCTTACCTGTAGCGTTGGTCTTT
CTGCCCGCTGCAGATATTCCAACTTTTGTTGGTGAAGGTAAATGTGATCTTGGTATAACTGGTGTTGACCAAGTTCGTGAATCTAAC
GTCGACGTAGACTTAGCAATCGATTTGCAATTTGGTAACTGTAAATTGCAGGTACAAGTCCCCGTAAATGGCGAGTATAAAAAGCCA
GAACAGTTAATTGGCAAAACCATTGTTACCAGTTTCGTGAAACTTGCTGAAAAATACTTTGCCGATTTGGAAGGTACTACTGTTGAA
AAAATGACCACAAGGATAAAGTTTGTCAGTGGTTCCGTGGAGGCATCATGTGCTCTGGGAATTGGTGATGCTATTGTAGATCTTGTA
GAGAGTGGTGAGACAATGAGGGCAGCAGGTTTAGTTGATATTGCCACCGTCCTAAGCACAAGTGCCTACCTAATAGAATCAAAGAAC
CCAAAGAGCGATAAGAGTTTGATTGCTACTATCAAATCAAGAATTGAAGGTGTCATGACCGCTCAAAGGTTCGTTTCATGTATTTAT
AACGCACCTGAAGACAAGCTGCCTGAACTGTTGAAGGTGACGCCTGGCCGTAGAGCACCAACCATTTCCAAAATTGACGATGAAGGA
TGGGTTGCTGTTAGTTCCATGATTGAGAGAAAAACGAAGGGTGTTGTTTTAGATGAATTGAAAAGACTCGGCGCATCTGATATCATG
GTTTTCGAAATTTCTAATTGTCGTGTATAA**TCAGTACTGACAATAAAAAGATTCTTGTTTTCAAGAACTTGTCATTTGTATAGTTTT
TTTATATTGTAGTTGTTCTATTTTAATCAAATGTTAGCGTGATTTATATTTTTTTTCGCCTCGACATCATCTGCCCAGATGCGAAGT
TAAGTGCGCAGAAAGTAATATCATGCGTCAATCGTATGTGAATGCTGGTCGCTATACTGCTGTCGATTCGATACTAACGCCGCCATC
CAGTGTCGAAAACGAGCTCTCGAGAACCCTTAATATAACTTCGTATAatgtatgcTATACGAAGTTATTAGAATTTTGAATTTGGTT
AAGAAAAGAAACTTACCAAGATGGACTTTTTTAATAAACATACATAATCACTACATATAGGTGCGTAATAATAAGTTTTTATTTTTT
TTTTCTTAATTCAGCGAGCTTTCTACATTTCAATTTTCTTGAATTTACCAGCTCTGATAAATCAAAGTTCAATGTCCGAA

Sequence of *AfCyp51A-6×His* (mutation I301T) cassette in *S. cerevisiae PDR5* locus:*PDR5::AfCyp51A-6×His-LoxP_His1* (mutation I301T in AfCYP51A) (PDR5Fv3 / PDR5_186DS_R)

The codon optimised *AfCyp51A-6×His* open reading frame (ORF) is highlighted in yellow. The desired mutation ATA → ACA is highlighted in pink. The *His1* selective marker is in bold between LoxP sites highlighted in red.

TCGCATTCTGCGCCTTCGAGCACAGGATAAGTTGCAGGAAGCCATCACATCTATGCAACGATTATCACGACACAACCTTGCCGCCGA
GAAAACGTCCGTGGAGAACCATTCGGTCGATTGCTTCCCACGGAACGAGTGGACTGAAACTTAAGACTGCCCCTCTCTTTCCGCGGA
ATCGCTCATGCCGCGGTGCCACAACATTTTCAGATTTACTAAGACTCCGGTGAGTGTGGGCTCACCCGCGGGTCGTGATCACGATTC
AGCACCCTTTGGACTCGTGATTCCGTGGAAAGGTCAGATCTGTATTCCTACTTATGGTAATGTGCTAAAAAAAGAGAAATGTCTCCG
CGGAACTCTTCTACGCCGTGGTACGATATCTGTTGAACGTAATCTGAGCAATACAAACAAGGCCTCTCCTATACATATATAATTGTG
ATGTGCATAACCTTATGGCTGTTCGCTTTTATTACCATACCTTAGAATGAAATCCAAAAGAAAAAAGTCACGCAAAGTTGCAAACAT
ATAACAACTGTGTTAGTTATCACTCGACTTTGTTATTCTAATTATAAATAAATTGGCAACTAGGAACTTTCGAAAAAGAAATTAAAG
ACCCTTTTAAGTTTTCGTATCCGCTCGTTCGAAAGACTTAATTAAAAAATGGTCCCTATGCTCTGGTTGACCGCATACATGGCAGTT
GCAGTTCTCACAGCCATCTTACTAAACGTAGTTTATCAGTTGTTTTTCCGTTTGTGGAACAGAACAGAACCTCCAATGGTGTTCCAT
TGGGTGCCATACCTGGGCTCAACCATTTCATACGGTATTGATCCATACAAGTTCTTCTTCGCTTGTAGAGAGAAATACGGTGACATT
TTCACATTCATTCTACTGGGACAAAAGACAACAGTTTACCTGGGTGTTCAAGGTAATGAATTCATACTGAATGGCAAGTTAAAAGAC
GTCAATGCCGAAGAGGTTTATTCTCCATTGACAACACCAGTTTTCGGCTCCGATGTTGTTTACGATTGCCCTAACTCTAAGCTTATG
GAACAGAAAAAGTTTATCAAGTACGGTCTTACCCAATCAGCCTTGGAATCACATGTCCCTTTAATCGAGAAGGAAGTGCTTGACTAC
CTAAGAGATAGCCCTAACTTTCAAGGTTCTAGTGGTAGAGTCGATATTTCTGCAGCAATGGCAGAAATCACAATCTTTACTGCGGCT
AGAGCTTTACAAGGTCAAGAGGTAAGATCTAAATTGACTGCAGAATTTGCTGACTTGTACCACGATTTGGATAAAGGTTTCACTCCA
ATCAACTTTATGCTACCATGGGCCCCATTACCACACAACAAAAAGAGAGATGCTGCTCATGCTAGAATGAGATCAATCTATGTTGAC
ATTATCACCCAGAGAAGATTAGACGGGGAAAAAGACAGCCAAAAGTCTGATATGATTTGGAATTTGATGAATTGTACATACAAAAAT
GGTCAACAGGTACCTGATAAGGAGATTGCACATATGATGATCACACTTTTGATGGCCGGACAACATTCTTCTTCCTCTACATCAGCA
TGGATTATGTTGAGATTAGCTTCTCAACCAAAGGTATTAGAAGAGTTGTACCAAGAGCAACTCGCGAACTTAGGTCCTGCGGGTCCA
GATGGATCATTACCACCATTGCAATACAAGGACCTTGATAAGCTTCCTTTTCATCAACATGTCATTAGAGAAACTTTGAGAATTCAT
AGTAGTATCCACTCTATAATGAGAAAGGTCAAATCCCCATTACCTGTCCCAGGTACTCCATACATGATACCACCAGGCAGAGTTCTT
CTAGCATCACCTGGTGTAACTGCCCTGTCAGATGAGCATTTCCCAAACGCTGGCTGTTGGGATCCACATAGATGGGAAAACCAGGCC
ACCAAGGAACAGGAAAATGATAAGGTGGTTGATTACGGATATGGAGCTGTGTCAAAGGGTACTTCCAGCCCATACCTCCCATTTGGG
GCAGGACGTCACAGATGCATCGGAGAAAAGTTCGCTTACGTCAACTTAGGTGTTATCTTGGCTACAATAGTGAGACATTTGCGTCTA
TTCAACGTTGATGGGAAAAAGGGTGTTCCAGAAACAGATTACTCTTCTTTGTTCAGTGGTCCTATGAAGCCTTCAATTATCGGCTGG
GAAAAGAGATCTAAAAACACTTCCAAAGGCGGCCGCCATCATCACCATCATCATTAAATTCGGCCGCTTCTTTGGAATTATTGGAAG
GTAAGGAATTGCCAGGTGTTGCTTTCTTATCCGAAAAGAAATAAATTGAATTGAATTGAAATCGATAGATCAATTTTTTTCTTTTCT
CTTTCCCCATCCTTTACGCTAAAATAATAGTTTATTTTATTTTTTGAATATTTTTTATTTATATACGTATATATAGACTATTATTTA
TCTTTTAATGATTATTAAGATTTTTATTAAAAAAAAATTCGCTCCTCTTTTAATGCCTTTATGCAGTTTTTTTTTCCCATTCGATAT
TTCTATGTTCGGGTTCAGCGTATTTTAAGTTTAATAACTCGAAAATTCTGCGTTCGTTGGAGAAGCTTCGTACGCTGCAGGTCGACA
ACCCTTAATATAACTTCGTATAatgtatgcTATACGAAGTTATTAGGTCTAGAGATCTGTTTAGCTTGCCTCGTCCCCGCCGGGTCA
CCCGGCCAGCGACATGGAGGCCCAGAATACCCTCCTTGACAGTCTTGACGTGCGCAGCTCAGGGGCATGATGTGACTGTCGCCCGTA
CATTTAGCCCATACATCCCCATGTATAATCATTTGCATCCATACATTTTGATGGCCGCACGGCGCGAAGCAAAAATTACGGCTCCTC

GCTGCAGACCTGCGAGCAGGGAAACGCTCCCCTCACAGACGCGTTGAATTGTCCCCACGCCGCGCCCCTGTAGAGAAATATAAAAGG
TTAGGATTTGCCACTGAGGTTCTTCTTTCATATACTTCCTTTTAAAATCTTGCTAGGATACAGTTCTCACATCACATCCGAACATAA
ACAACCATGGATTTGGTGAACCATCTAACCGATAGACTACTGTTTGCAATCCCAAAGAAAGGTCGTTTATATTCTAAAAGTGTTTCT
ATTTTGAATGGTGCTGATATTACCTTTCACCGCTCTCAAAGATTAGACATTGCACTAAGCACAAGCTTACCTGTAGCGTTGGTCTTT
CTGCCCGCTGCAGATATTCCAACTTTTGTTGGTGAAGGTAAATGTGATCTTGGTATAACTGGTGTTGACCAAGTTCGTGAATCTAAC
GTCGACGTAGACTTAGCAATCGATTTGCAATTTGGTAACTGTAAATTGCAGGTACAAGTCCCCGTAAATGGCGAGTATAAAAAGCCA
GAACAGTTAATTGGCAAAACCATTGTTACCAGTTTCGTGAAACTTGCTGAAAAATACTTTGCCGATTTGGAAGGTACTACTGTTGAA
AAAATGACCACAAGGATAAAGTTTGTCAGTGGTTCCGTGGAGGCATCATGTGCTCTGGGAATTGGTGATGCTATTGTAGATCTTGTA
GAGAGTGGTGAGACAATGAGGGCAGCAGGTTTAGTTGATATTGCCACCGTCCTAAGCACAAGTGCCTACCTAATAGAATCAAAGAAC
CCAAAGAGCGATAAGAGTTTGATTGCTACTATCAAATCAAGAATTGAAGGTGTCATGACCGCTCAAAGGTTCGTTTCATGTATTTAT
AACGCACCTGAAGACAAGCTGCCTGAACTGTTGAAGGTGACGCCTGGCCGTAGAGCACCAACCATTTCCAAAATTGACGATGAAGGA
TGGGTTGCTGTTAGTTCCATGATTGAGAGAAAAACGAAGGGTGTTGTTTTAGATGAATTGAAAAGACTCGGCGCATCTGATATCATG
GTTTTCGAAATTTCTAATTGTCGTGTATAATCAGTACTGACAATAAAAAGATTCTTGTTTTCAAGAACTTGTCATTTGTATAGTTTT
TTTATATTGTAGTTGTTCTATTTTAATCAAATGTTAGCGTGATTTATATTTTTTTTCGCCTCGACATCATCTGCCCAGATGCGAAGT
TAAGTGCGCAGAAAGTAATATCATGCGTCAATCGTATGTGAATGCTGGTCGCTATACTGCTGTCGATTCGATACTAACGCCGCCATC
CAGTGTCGAAAACGAGCTCTCGAGAACCCTTAATATAACTTCGTATAatgtatgcTATACGAAGTTATTAGAATTTTGAATTTGGTT
AAGAAAAGAAACTTACCAAGATGGACTTTTTTAATAAACATACATAATCACTACATATAGGTGCGTAATAATAAGTTTTTATTTTTT
TTTTCTTAATTCAGCGAGCTTTCTACATTTCAATTTTCTTGAATTTACCAGCTCTGATAAATCAAAGTTCAATGTCCGAA

B. Mass spectrometry analysis of tryptic fragments from SDS-PAGE separated bands containing putative recombinant AfCYP51A or AfCYP51B

Sequences identified by mass spectrometry of a tryptic digest of the 59 kDa band containing putative AfCYP51A recovered from SDS-PAGE of crude membranes from strain A are shown in bold. Protein sequence coverage AfCYP51A is 21%.

MVPMLWLTAYMAVAVLTAILLNVVYQLFFRLWNRTEPPMVFHWVPYLGSTISYGIDPYKFFFACREKYGDIFTFILLGQKTTVYLGV
QGNEFILNGKLKDVNAEEVYSPLTTPVFGSDVVYDCPNSKLMEQKKFIK**YGLTQSALESHVPLIEKE**VLDYLR**DSPNFQGSSGR**VDI
SAAMAEITIFTAARALQGQEVRSK**LTAEFADLYHDLDK**GFTPINFMLPWAPLPHNKKRDAAHARMR**SIYVDIITQRRLDGEKDSQK**S
DMIWNLMNCTYKNGQQVPDKEIAHMMITLLMAGQHSSSSISAWIMLRLASQPKVLEELYQEQLANLGPAGPDGSLPPLQYKDLDKLP
FHQHVIRETLRIHSSIHSIMRK**VKSPLPVPGTPYMIPPGRV**LLASPGVTALSDEHFPNAGCWDPHRWENQATK**EQENDKVVDYGYGA**
VSKGTSSPYLPFGAGRHCIGEKFAYVNLGVILATIVRHLRLFNVDGKKGVPETDYSSLFSGPMKPSIIGWEKRSKNTSKGGRHHHHH
H

Sequences identified by mass spectrometry of a tryptic digest of the 60 kDa band of containing putative AfCYP51B recovered from SDS-PAGE of crude membranes from strain B are shown in bold. Protein sequence coverage is 32%.

MGLIAFILDGICKHCSTQSTWVLVGIGLLSILAVSVIINVLQQLLFKNPHEPPVVFHWFPFIGSTISYGIDPYKFFFDCRAKYGDIF
TFILLGKKTTVYLGTK**GNDFILNGK**LRDVCAEEVYSPLTTPVFGR**HVVYDCPNAKL**MEQKKFVK**YGLTSDALRSYVPLITDEVESFV**
KNSPAFQGHKGVFDVCK**TIAEITIYTASR**SLQGKEVRSKFDSTFAELYHNLDMGFAPINFMLPWAPLPHNRKRDAAQR**KLTETYMEI**
IKARRQAGSKKDSEDMVWNLMSCVYKNGTPVPDEEIAHMMIALLMAGQHSSSSTASWIVLR**LATRPDIMEELYQEQIRVLGSDLPPL**
TYDNLQKLDLH**AKVIKETLRLHAPIHSIIRAVKNPMAVDGTSYVIPTSHNVLSSPGVTARSEEHFPNPLEWNPHR**WDENIAASAEDD
EK**VDYGYGLVSKGTNSPYLPFGAGR**HRCIGEQFAYLQLGTITAVLVRLFRFRNLPGVDGIPDTDYSSLFSKPLGRSFVEFEKRESAT
KAGGRHHHHHH

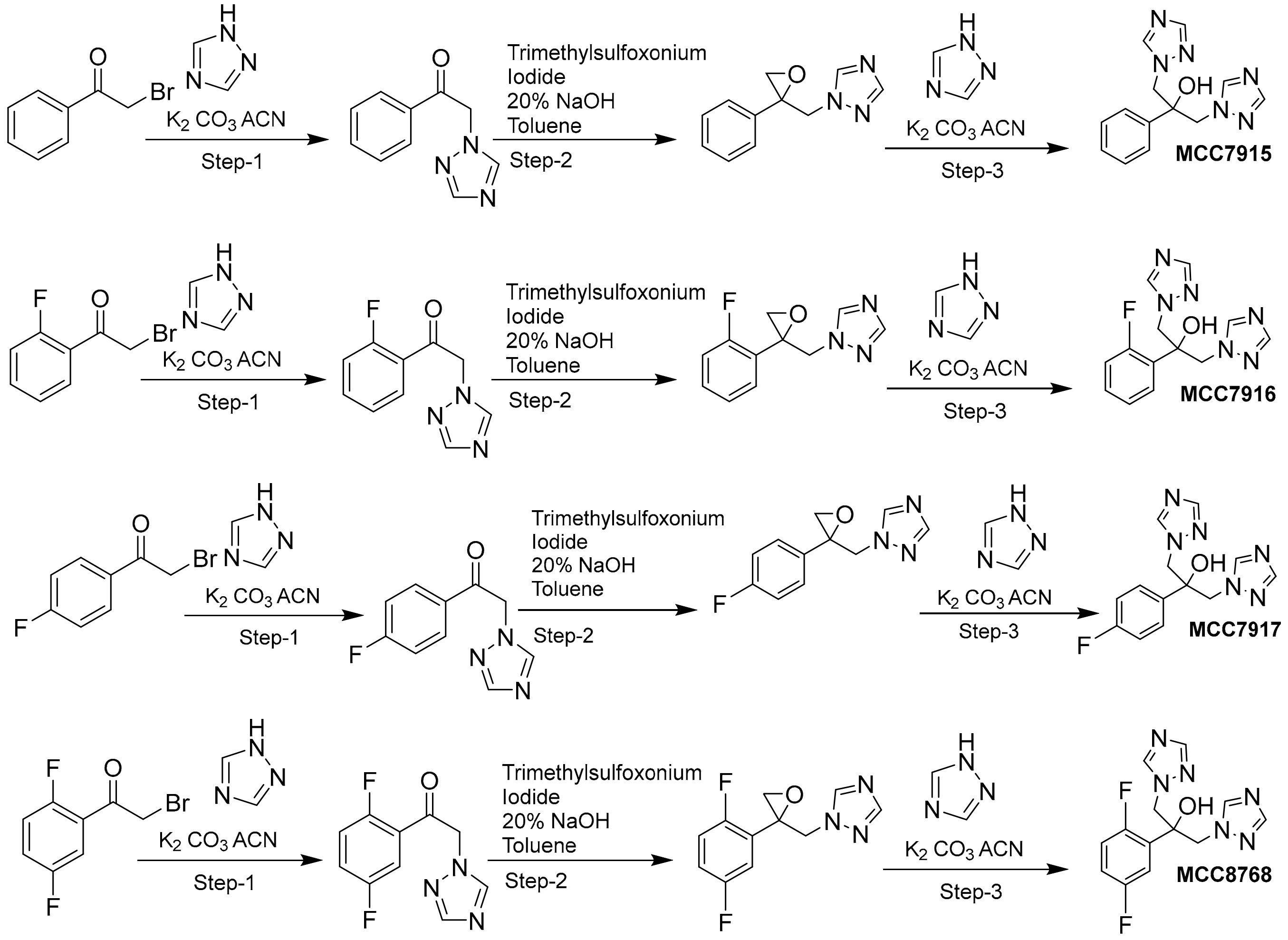

Figure S8. Synthesis schemes for congeners of fluconazole (FLC). Step-1: The starting material (SM), a suitable benzoylbromide (1g) dissolved in acetonitrile (ACN) and K_2CO3 (1.5 eq), followed by 1,2,4-triazole (1.1 eq), was heated to 45°C for 45min. Step-2: The product from step 1 (0.5g) dissolved in toluene and trimethylsulfoxonium iodide (2eq), followed by 20% NaOH (10 eq) solution, was heated to 80°C for 1hr. Step-3: The purified product from step 2 (0.15g) dissolved in ACN, together with 1,2,4-Triazole (1.2 eq) and K_2CO3 (2.0 eq), was heated to 60°C for 16hr. Material generated in each step was subjected to flash purification on silica gel, preparative HPLC (Waters Autopur-System), precipitation and reprecipitation, and crystallization to ensure purity of the required products. The purity and identity of products were determined by FPLC, mass spectrometry and NMR.

Figure S9. FPLC, mass spectrometry and NMR analysis of congeners of FLC.

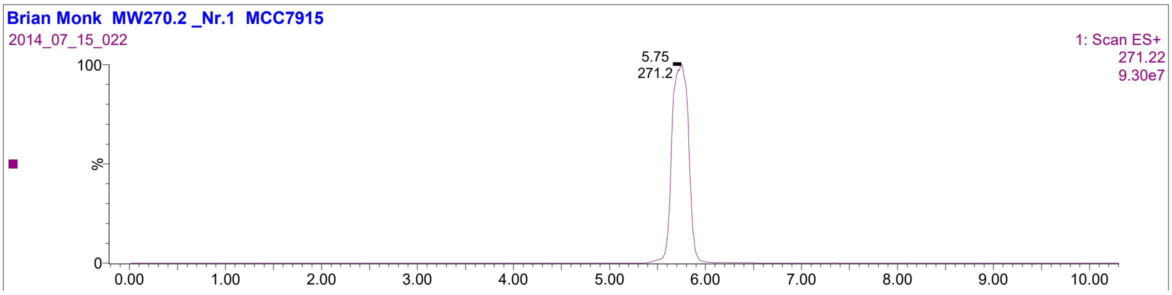

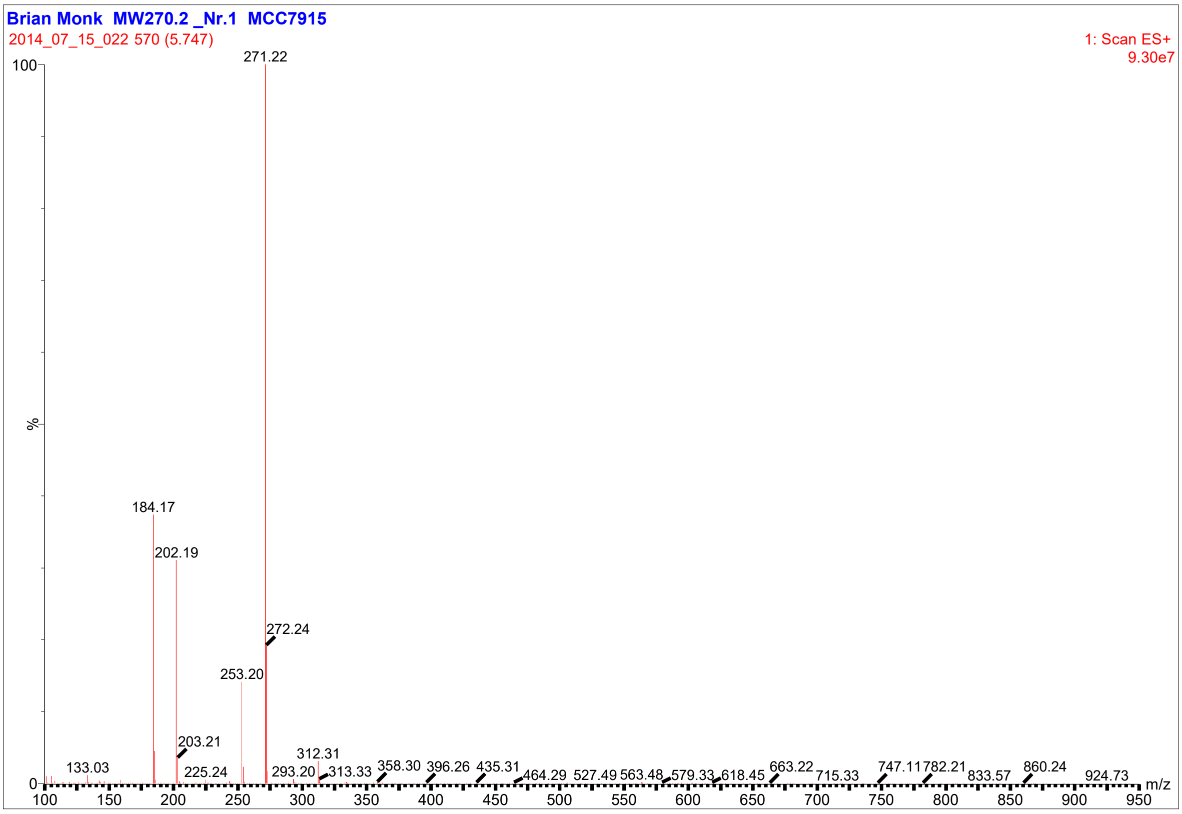

MCC7915: 2-phenyl-1,3-bis(1H-1,2,4-triazol-1-yl)propan-2-ol / $C_{13}H_{14}N_6O$

^{1}H NMR (300 MHz, CD_3CN) δ (ppm) = 7.94 (s, 2H, 3-H-triazol), 7.71 (s, 2H, 5-H-triazol), 7.15-7.28 (m, 5H, H-phenyl), 4.89 (s, 1H, OH), 4.61 (d, ^2J = 14.5 Hz, 2H, 1-H- and 3-H-propyl), 4.44 (d, ^2J = 14.5 Hz, 2H, 1-H- and 3-H-propyl).

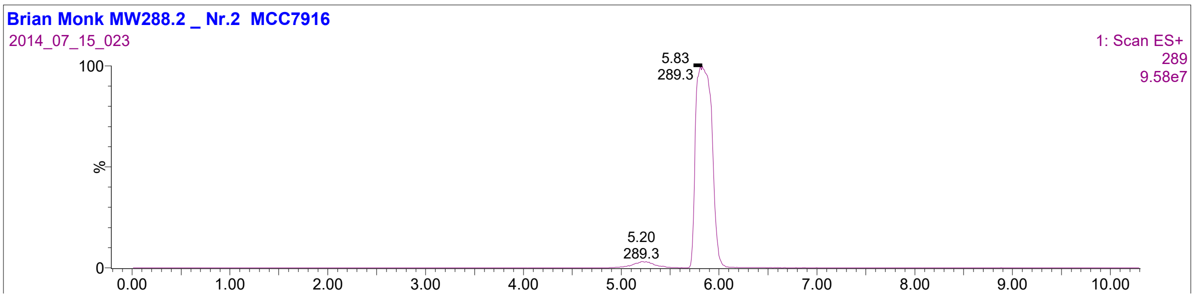

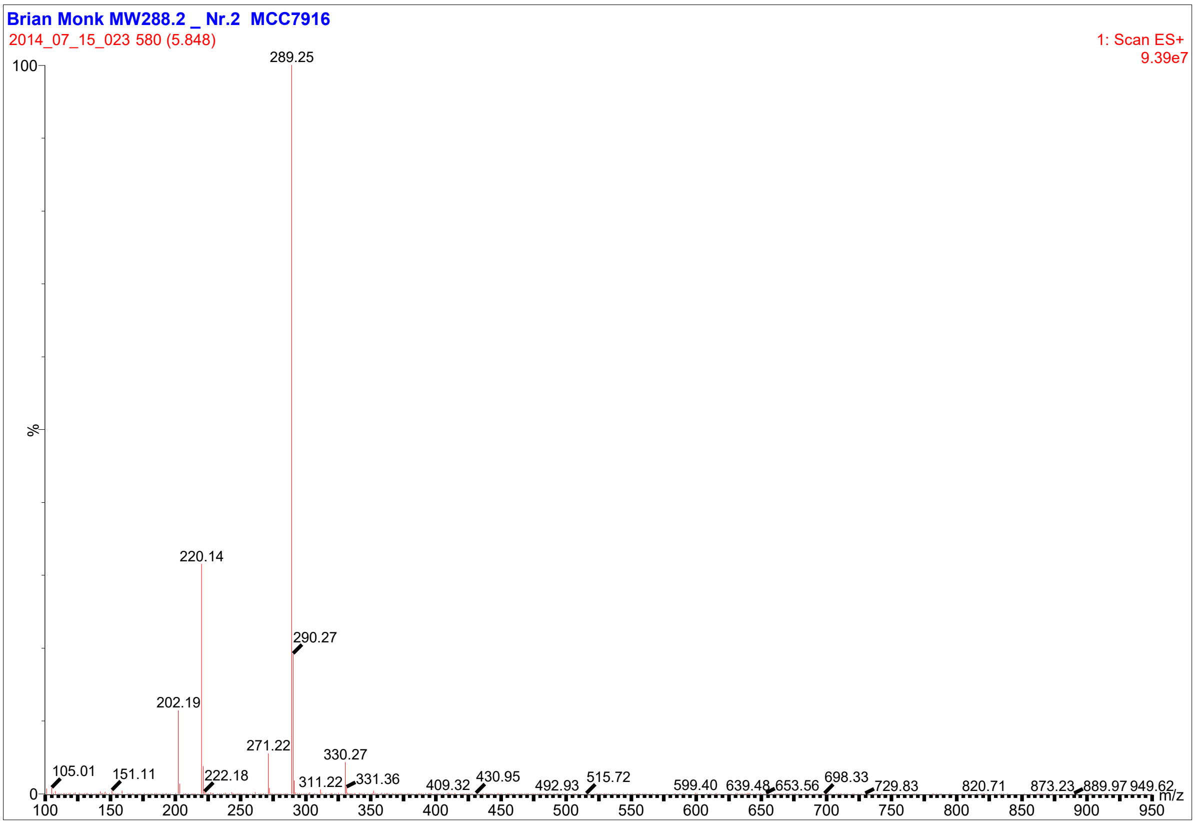

MCC7916: 2-(2-fluorophenyl)-1,3-bis(1H-1,2,4-triazol-1-yl)propan-2-ol / $C_{13}H_{13}FN_6O$

^{1}H NMR (300 MHz, CD_3CN) δ (ppm) = 8.04 (s, 2H, 3-H-triazol), 7.67 (s, 2H, 5-H-triazol), 7.28-7.18 (m, 2H, 4-H-6-H-phenyl), 7.08-6.94 (m, 2H, 3-H-5-H-phenyl), 5.02 (s, 1H, OH), 4.77 (d, ^2J = 14.5 Hz, 2H, 1-H- and 3-H-propyl), 4.50 (d, ^2J = 14.5 Hz, 2H, 1-H- and 3-H-propyl).

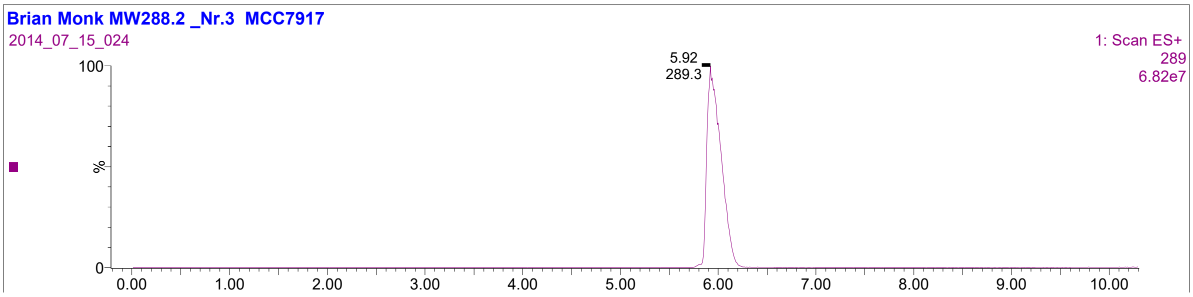

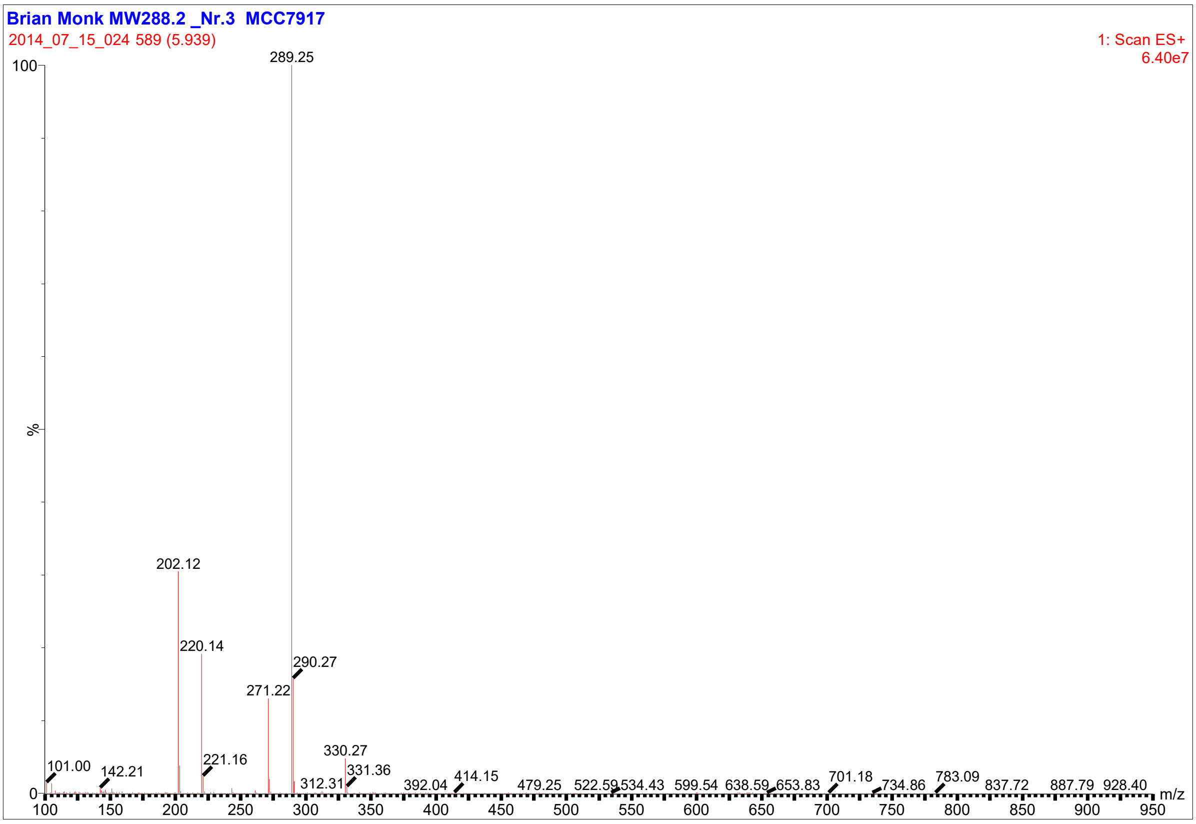

MCC7917: 2-(4-fluorophenyl)-1,3-bis(1H-1,2,4-triazol-1-yl)propan-2-ol / $C_{13}H_{13}FN_6O$

^{1}H NMR (300 MHz, CD_3CN) δ (ppm) = 7.97 (s, 2H, 3-H-triazol), 7.73 (s, 2H, 5-H-triazol), 7.30-7.26 (m, 2H, 2-H-6-H-phenyl), 6.99-6.93 (m, 2H, 3-H-5-H-phenyl), 4.95 (s, 1H, OH), 4.61 (d, ^2J = 14.5 Hz, 2H, 1-H- and 3-H-propyl), 4.45 (d, ^2J = 14.5 Hz, 2H, 1-H- and 3-H-propyl).

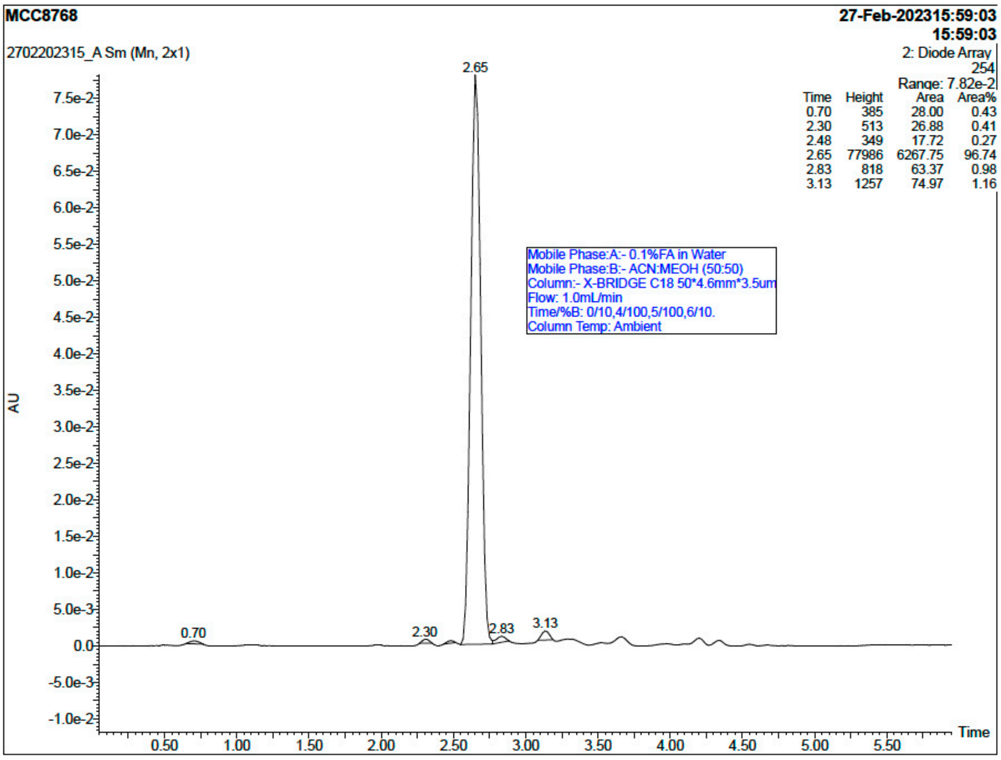

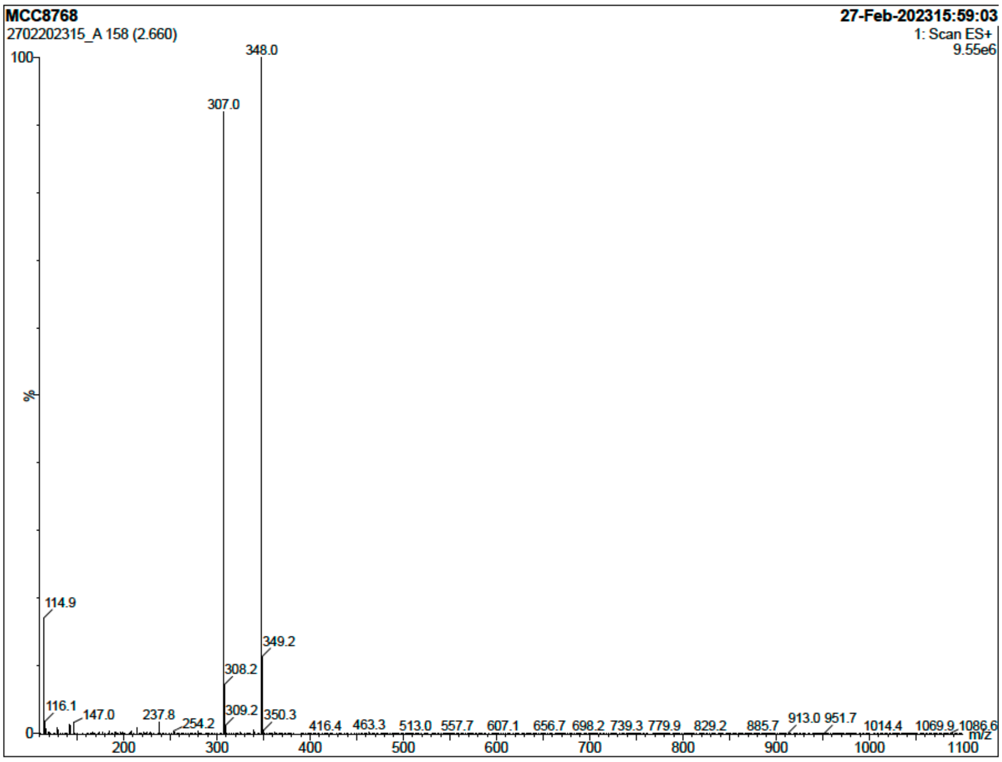

MCC8768: 2-(2,5-difluorophenyl)-1,3-bis(1,2,4-triazol-1-yl)propan-2-ol

$C_{13}H_{12}F_2N_6O$ ^{1}H NMR (600 MHz, D_6-DMSO) δ(ppm) = 8.30 (s, 2H), 7.76 (s, 2H), 7.20 (ddd, *J* = 11.0, 9.0, 4.5 Hz, 1H), 7.09 (ddt, *J* = 8.9, 7.1, 3.4 Hz, 1H), 6.84 (ddd, *J* = 9.6, 6.0, 3.3 Hz, 1H), 6.44 (s, 1H), 4.72 (d, *J* = 14.5 Hz, 2H), 4.54 (d, *J* = 14.4 Hz, 2H).

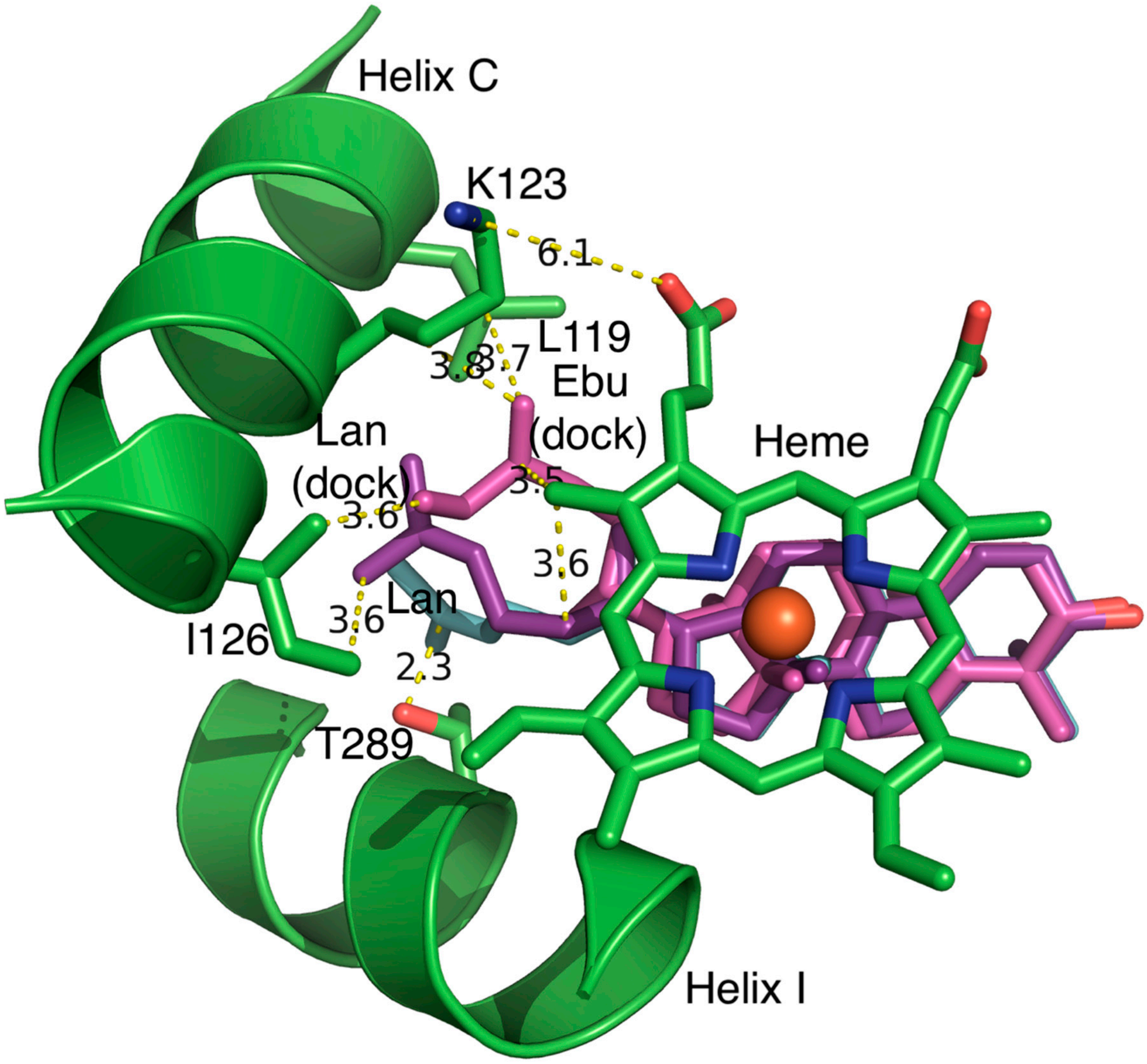

Figure S10. Superposition of lanosterol and docking of lanosterol and eburicol in a homology model of AfCYP51A obtained using HsCYP51 (PDB ID: 6UEZ) as template. The heme, helix I and helix C in green. Lanosterol is overlaid from the HsCYP51 crystal structure and top scoring docked eburicol (Ebu (dock)) and lanosterol (Lan (dock)) conformations are shown. Distances in the figures are given in Å.

A

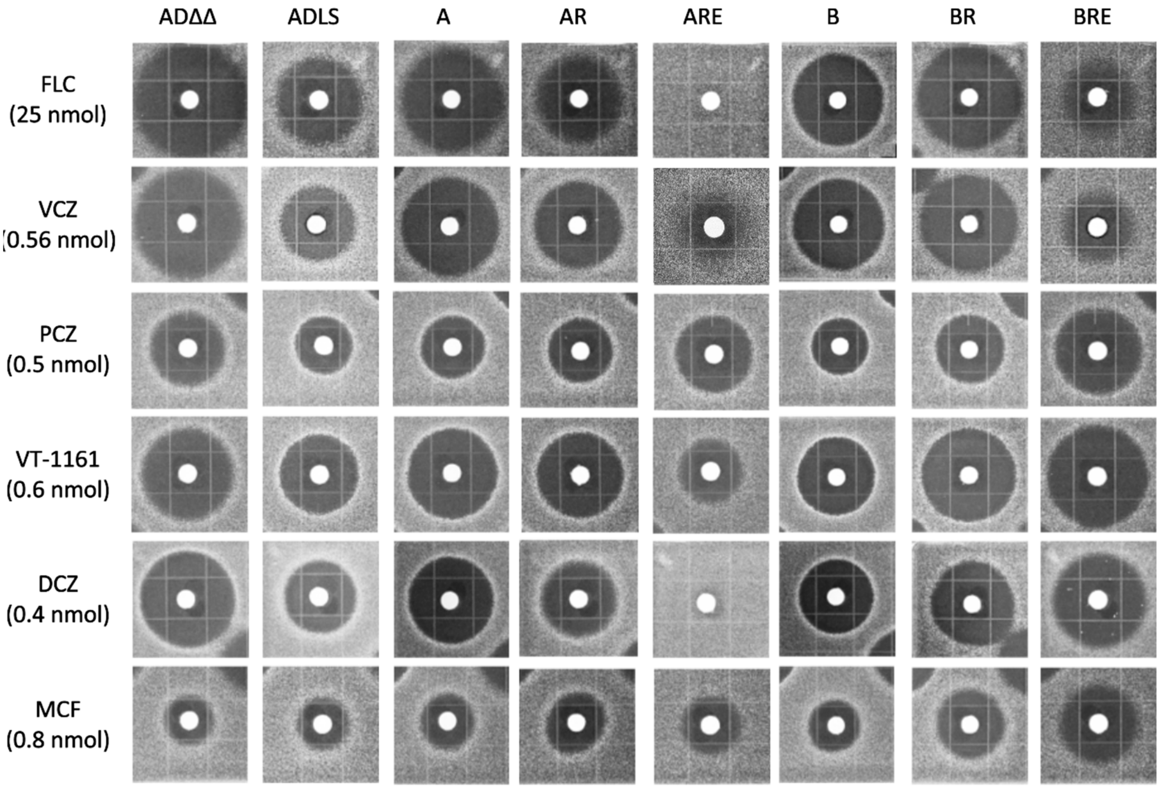
ADΔΔ
ADLS
A
AR
ARE
B
BR
BRE
FLC (25 nmol)
VCZ (0.56 nmol)
PCZ (0.5 nmol)
VT-1161 (0.6 nmol)
DCZ (0.4 nmol)
MCF (0.8 nmol)

B

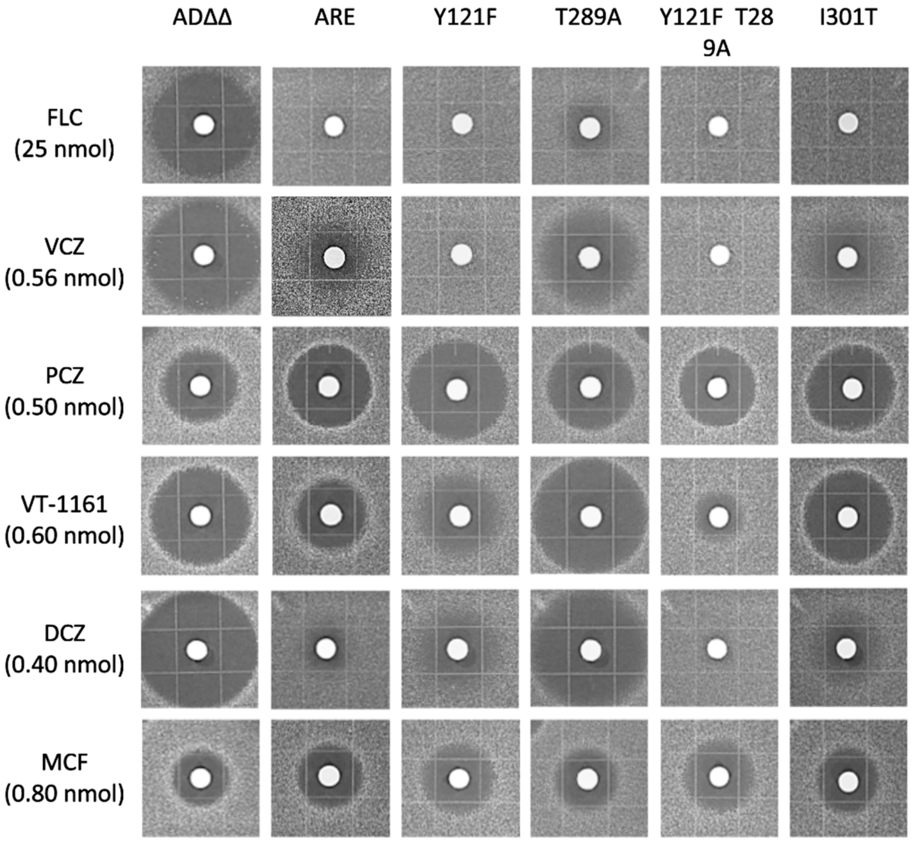
ADΔΔ
ARE
Y121F
T289A
Y121F T28 9A
I301T
FLC (25 nmol)
VCZ (0.56 nmol)
PCZ (0.50 nmol)
VT-1161 (0.60 nmol)
DCZ (0.40 nmol)
MCF (0.80 nmol)

Figure S11. Response of host and recombinant *S. cerevisiae* strains to azole drugs and MCF in agarose diffusion assays. (A) Strains expressing wild type ScCYP51, AfCYP51A or AfCYP51B, (B) Strains expressing mutant AfCYP51A.

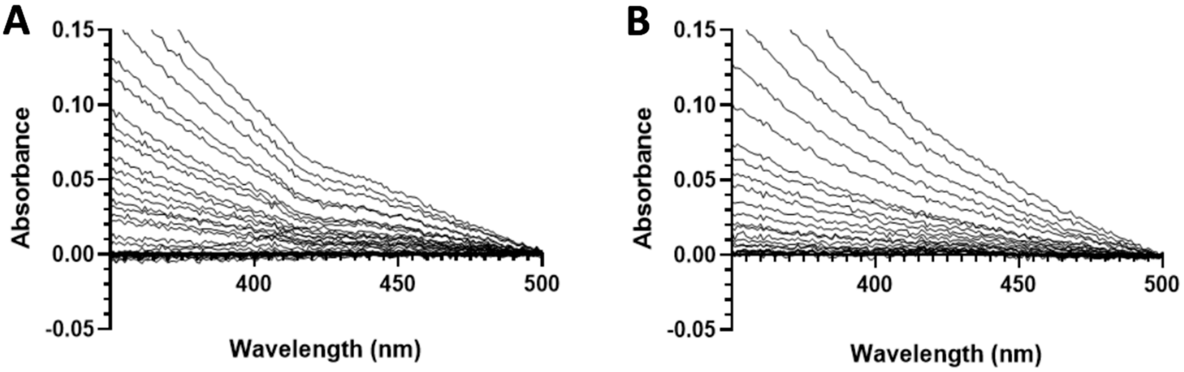

Figure S12. Type I difference spectra for the binding of eburicol (A) and lanosterol (B) to Ni-NTA affinity purified AfCYP51A-6×His. The AfCYP51A-6×His (5 μM) did not give the expected type I binding response for concentrations up to 300 μM of lanosterol or the substrate eburicol.
